# Cytosolic protein translation regulates cell asymmetry and function in early TCR activation of human CD8^+^ T lymphocytes

**DOI:** 10.3389/fimmu.2024.1411957

**Published:** 2024-07-24

**Authors:** Álvaro Gómez-Morón, Ilya Tsukalov, Camila Scagnetti, Clara Pertusa, Marta Lozano-Prieto, Pedro Martínez-Fleta, Silvia Requena, Pilar Martín, Aranzazu Alfranca, Enrique Martin-Gayo, Noa B Martin-Cofreces

**Affiliations:** ^1^ Immunology Service, Instituto de Investigación Sanitaria del Hospital Universitario La Princesa, IIS- Princesa, Universidad Autónoma de Madrid (UAM), Madrid, Spain; ^2^ Department of Immunology, Ophthalmology and ENT, School of Medicine, Universidad Complutense de Madrid and 12 de Octubre Health Research Institute (imas12), Madrid, Spain; ^3^ Medicine Faculty, Universidad Autónoma de Madrid (UAM), Madrid, Spain; ^4^ Videomicroscopy Unit, Instituto de Investigación Sanitaria del Hospital Universitario La Princesa, IIS-Princesa, Universidad Autónoma de Madrid (UAM), Madrid, Spain; ^5^ Centro de Investigación Biomédica en Red de Enfermedades Cardiovasculares (CIBERCV), Instituto de Salud Carlos III, Madrid, Spain; ^6^ Area of Vascular Pathophysiology, Laboratory of Regulatory Molecules of Inflammatory Processes, Fundación Centro Nacional de Investigaciones Cardiovasculares-Carlos III, Madrid, Spain; ^7^ Centro de Investigación Biomédica en Red Enfermedades Infecciosas (CIBERINFECC), Instituto de Salud Carlos III, Madrid, Spain; ^8^ Area of Vascular Pathophysiology, Laboratory of Intercellular Communication, Fundación Centro Nacional de Investigaciones Cardiovasculares-Carlos III, Madrid, Spain

**Keywords:** protein translation, cell asymmetry, cytotoxic CD8^+^ T lymphocytes, cytoskeleton, metabolism, mitochondria, T cell activation, immunological synapse

## Abstract

**Introduction:**

CD8^+^ cytotoxic T lymphocytes (CTLs) are highly effective in defending against viral infections and tumours. They are activated through the recognition of peptide–MHC-I complex by the T-cell receptor (TCR) and co-stimulation. This cognate interaction promotes the organisation of intimate cell–cell connections that involve cytoskeleton rearrangement to enable effector function and clearance of the target cell. This is key for the asymmetric transport and mobilisation of lytic granules to the cell–cell contact, promoting directed secretion of lytic mediators such as granzymes and perforin. Mitochondria play a role in regulating CTL function by controlling processes such as calcium flux, providing the necessary energy through oxidative phosphorylation, and its own protein translation on 55S ribosomes. However, the effect of acute inhibition of cytosolic translation in the rapid response after TCR has not been studied in mature CTLs.

**Methods:**

Here, we investigated the importance of cytosolic protein synthesis in human CTLs after early TCR activation and CD28 co-stimulation for the dynamic reorganisation of the cytoskeleton, mitochondria, and lytic granules through short-term chemical inhibition of 80S ribosomes by cycloheximide and 80S and 55S by puromycin.

**Results:**

We observed that eukaryotic ribosome function is required to allow proper asymmetric reorganisation of the tubulin cytoskeleton and mitochondria and mTOR pathway activation early upon TCR activation in human primary CTLs.

**Discussion:**

Cytosolic protein translation is required to increase glucose metabolism and degranulation capacity upon TCR activation and thus to regulate the full effector function of human CTLs.

## Introduction

CD8^+^ cytotoxic T lymphocytes (CTLs) are recognised as the primary adaptive immune cells responsible for eliminating virally infected cells or tumour cells (1, 2). To achieve this, CTLs have developed three mechanisms that enable them to serially kill target cells. The first mechanism involves the secretion of perforin and granzyme-enriched lytic granules (3–5). Perforin is released into the interface between CTLs and target cells, permeabilising the target cell membrane and allowing the entry of potent serine proteases, known as granzymes. These granzymes induce apoptosis, resulting in the elimination of the target cell (6). The second mechanism involves the interaction of the death receptor FasL in the plasma membrane of CTLs in trans with Fas, its cognate receptor on the target cell. This binding triggers extrinsic apoptosis through the activation of caspase-8 and direct proteolysis of intracellular substrates (7, 8). In addition, a third mechanism involving the release of supramolecular attack particles (SMAPs) has recently been described (9, 10). These particles are encapsulated by a thrombospondin-1 (TSP-1)-rich glycoprotein shell and release aggregates of lytic molecules. SMAPs are stored in multicore lytic granules within CTLs and remain active for hours after their release, acting as autonomous killing units (9, 10). The mechanism by which SMAPs damage target cells is still under investigation.

CTLs are activated when their T-cell receptors (TCRs) bind to complexes formed between antigenic peptides and MHC class I molecules (pMHC) on the surface of target cells. Cognate interaction with pMHC complexes induces the formation of intimate contacts by organising a specialised signalling and secretory platform at the cell–target interface known as immunological synapse (IS) (11–13). This process regulates the dynamics and organisation of F-actin and microtubules (MTs) to ensure the proper propagation of the activating signal. These changes are necessary for sustained T-cell activation and effector function (14–18). The tubulin and actin cytoskeletons work together to coordinate the delivery of secretory granules at the site of CTL–target cell contact (14–19). MTs emanating from the translocated centrosome, which acts as a microtubule-organising centre (MTOC), serve as tracks for the transport of organelles, such as mitochondria and vesicles. These organelles provide energy and allow for the renewal of molecules at the cell contact (20–22). Mitochondria regulate the organisation of the actin cytoskeleton at contact with the antigen-presenting or target cell (23) and depend on the tubulin cytoskeleton to move there and to achieve maximum functionality (24, 25). Mitochondrial polarisation requires the plus-end MT protein end binding protein-1 (EB1). Upon T-cell activation, mitochondria from EB1-deficient cells exhibit reduced capacity to produce ATP from glucose (25).

Cytosolic protein synthesis is a highly energy-demanding process for T cells, particularly following activation (26). Proteostasis, or protein turnover in the cell, is highly regulated during TCR activation and depends on the mTOR complex to activate translation and regulate autophagy. The regulation of proteostasis during activation of T cells may be relevant to understanding changes in immune responses over time (24, 26–31). mTORC1 promotes translation by increasing the activity of eukaryotic translation initiation factor 4E (eIF4E) binding proteins (4E-BP1, 2, and 3) and p70 ribosomal S6 kinase (S6K), which phosphorylates the ribosomal S6 subunit, promoting eukaryotic translation (32). Reduced TCR expression in CD4 T cells results in reduced mTOR and S6K activity in short-term activated human CD4^+^ T cells (27), which supports TCR-dependent activation of the mTOR pathway. Furthermore, in murine T cells, mTORC1 increases TCR-dependent cell growth by controlling the expression of >2,300 proteins in CD8^+^ T cells (33). In addition to protein translation, mTOR regulates metabolism, differentiation, and exhaustion in T cells (34–37). Indeed, for optimal CTL effector function, mitochondria are critical since they produce the required ATP and support other CTL functions such as migration, differentiation, and TCR signalling by regulating calcium flux (31, 33–38). CTLs require correct translation at mitochondrial 55S ribosomes to help CD8^+^ cytotoxic T cell-mediated prolonged killing (39). Previously, the folding of *de novo* proteins was correlated with tubulin polymerisation and reorganisation of the centrosome and lysosomes in activated CD4^+^ T cells (24). However, the specific involvement of cytosolic protein translation in regulating the short-term dynamics and function of mitochondria and lytic granules in TCR-activated human CTLs has not been widely studied. To ascertain whether translation at 80S eukaryote ribosomes is involved in early TCR activation to promote CTL effector function, we herein analysed the role of cytosolic translation in the reorganisation of human primary CD8^+^ CTLs to adopt cell asymmetry through chemical inhibition of *de novo* protein synthesis. We observed that inhibition of translation shortly before TCR activation prevents proper reorganisation of the tubulin cytoskeleton and asymmetric distribution of mitochondria. mTOR pathways are defectively activated in these cells, and glucose metabolism through glycolysis and oxidative phosphorylation (OXPHOS) is affected. In this context, the movement of lytic granules at the cell contact area with stimulating surfaces and degranulation of CTLs is prevented. We therefore linked the activity of 80S ribosomes and mitochondria with the ability of human CTLs to exert their effector functions *in vitro*.

## Materials and methods

### Antibodies and reagents

Antibodies used include pS2448 mTOR, mTOR, pS235/S236 S6, S6 ribosomal subunit, pS473 Akt, Akt, pY783 PLCγ1, PLCγ1, pS402/Y404 Erk1/2, Erk1/2, and PCM1 (all 1:1,000 WB, 1:100 IF; Cell Signaling Technology, Beverly, MA, USA). Anti-β-actin (clone AC-15; 1:2,000 WB), anti-α-tubulin (clone DM1A; 1:2,000 WB), anti-α-tubulin fluorescein isothiocyanate (FITC)-conjugated (clone DM1A; 1:100 IF), and anti-K40-α-tubulin (clone 6–11B-1; 1:2,000 WB) were from Sigma-Aldrich (St. Louis, MO, USA). Anti-Kinesin Heavy Chain (clone H1; 1:500 WB), anti-Kinesin Light Chain (clone L1; 1:1,000 WB), and anti-Dynein Intermediate Chains (clone 74.1; 1:500 WB) were from Merck Millipore (Darmstadt, Germany). Anti-Tom20 (clone FL-145; 1:50 IF) was from Santa Cruz Biotechnology (Dallas, TX, USA). Anti-VDAC (1:500 WB) antibody was from Thermo Fisher Scientific (Waltham, MA, USA). αCD3/αCD28 tetramers (ImmunoCult human T cell activator) were from StemCell Technologies (Vancouver, BC, Canada). Anti-CD3ϵ (clone HIT3a), anti-CD28 (clone CD28.2), anti-p150-Glued [1/p150Glued (RUO); 1:1,000 WB], anti-CD8-V500 (1:100 FACS), and anti-CD14-PE (1:100 FACS) were from BD Biosciences (San Jose, CA, USA). Anti-CD8-PE (1:100 FACS), anti-CD69-V450 (1:200 FACS), anti-CD3-PerCP/Cy5.5 (1:200 FACS), anti-CD8-PerCP/Cy5.5 (1:100 FACS), anti-CD107a-APC (1:200 FACS), anti-Perforin-PE/Cy7 (1:100 FACS), anti-Granzyme B-V450 (1:100 FACS), anti-IFN-γ-FITC (1:100 FACS), Alexa Fluor 488 anti-puromycin antibody (clone 2A4, 1:20 FACS), and anti-TNFα-PerCP (1:100 FACS) were from BioLegend (San Diego, CA, USA). Ghost dye Red 780 and Ghost dye Violet 510 Viability Dyes (all 1:1,000 FACS) were purchased from Tonbo Biosciences (San Diego, CA, USA). The following secondary reagents were used: goat anti-rabbit and goat anti-mouse highly cross-adsorbed secondary antibodies conjugated to Alexa Fluor 568 (A11036 and A11031, respectively; 1:500 IF) or 647 (A-21443 and A-21236, respectively; 1:500 IF) were purchased from Thermo Fisher Scientific. Horseradish peroxidase (HRP)-conjugated secondary antibodies for WB (anti-rabbit 31460, mouse 31430; all 1:10,000) were purchased from Thermo Fisher Scientific.

Reagents and probes were as follows: MitoTracker Orange CMTMRos was from Life Technologies (Invitrogen, Carlsbad, CA, USA). Nonyl acridine orange was from Sigma-Aldrich. LysoTracker Red DND-99 was from Thermo Fisher Scientific.

### Cell isolation and culture

For the isolation of CD8^+^ T lymphocytes, human peripheral blood mononuclear cells (PBMCs) were isolated from buffy coats of healthy donors provided by the *Centro de Transfusiones de la Comunidad de Madrid* under an agreement with the *Hospital Princesa* (Madrid) and approved by the CEIm of the *Hospital Princesa*, according to government ethical approval. After separation on a BioColl gradient (Biochrom, Berlin, Germany), cells were purified using the negative selection EasySep kit (StemCell Technologies) for human CD8^+^ T cells (purity > 95%). For CTL differentiation, cells were then cultured for 24 h at 2 M/mL onto P6 wells on RPMI 1640 medium (Gibco-Invitrogen) supplemented with 10% fetal bovine serum (FBS; Invitrogen) in the presence of 20 μL/mL ImmunoCult Human CD3/CD28 T cell Activator (StemCell Technologies), and IL-2 (50 U/mL, StemCell Technologies) was added to the culture medium 24 h after stimulation and every 2 days to induce lymphocyte proliferation. Experiments were performed on days 4–6 of differentiation when CTLs were mature.

### Immunofluorescence and microscopy

For imaging through epifluorescence combined with deconvolution, CTLs were treated with cycloheximide (CHX; 20 μg/mL, Sigma-Aldrich), puromycin (PURO; 50 µg/mL, Invivogen, San Diego, CA, USA), or vehicle (CTRL) for 1 h at 37°C. Cells were then activated with anti-CD3ϵ mAb (10 μg/mL; HIT3a clone) and anti-CD28 mAb (3 μg/mL; CD28.2 clone)-coated latex microbeads (6.4 μm in diameter) for 30 min at 37°C and were allowed to spread over poly-l-Lys-coated coverslips. Controls were CTLs conjugated with human γ-globulin (100 μg/mL)-coated beads. For confocal microscopy imaging, CTLs were seeded for 30 min, allowing them to spread over stimulatory surfaces coated with anti-CD3ϵ (3 μg/mL) and anti-CD28 (1 μg/mL) monoclonal antibodies previously diluted in bicarbonate buffer (0.1 M NaHCO_3_ and 0.32 M Na_2_CO_3_).

Samples were processed as described (24, 40). In brief, cells were fixed with 4% paraformaldehyde in PHEM (PIPES 30 mM, HEPES 20 mM, EGTA 2 mM, and MgCl_2_ 1 mM, pH 6.9) containing 0.12 M sucrose for 10 min [room temperature (R/T)], permeabilised with TX-100 (0.2%) in PHEM for 5 min at R/T, and blocked with PHEM containing 100 μg/mL γ-globulin, 3% bovine serum albumin (BSA), and 0.2% azide for 30 min at R/T. Cells were sequentially stained with the indicated primary antibodies (1–10 μg/mL) followed by Alexa Fluor 488-, 568-, or 647-conjugated secondary antibodies (4 μg/mL) or FITC-conjugated anti-α-tubulin (0.1 μg/mL). Samples were mounted on ProLong gold or ProLong gold-DAPI (Invitrogen).

Confocal images were a series of fluorescence and brightfield images acquired using a TCS SP5 confocal laser scanning unit (Leica Microsystems, Wetzlar, Germany) attached to an inverted epifluorescence microscope (DMI6000) fitted with an HCX PL APO 63×/1.40–0.6 oil objective. Epifluorescence images were acquired as a Z-series of fluorescence and brightfield images under a THUNDER Imager Live Cell & 3D Cell Culture & 3D Assay and processed with the accompanying thunder algorithm for deconvolution (Leica Microsystems). A 100× objective was used. Images were acquired and processed with the accompanying confocal software (LCS; Leica) and assembled using ImageJ software (http://rsbweb.nih.gov/ij/). Polarisation of mitochondria to the IS was calculated using the Fiji plugin “*Synapse measure*” (http://rsbweb.nih.gov/ij/) as described (41). This program provides a polarisation ratio from accurate measurements of localised immunofluorescence by comparing fluorescence signals from multiple regions of the T cell and T cell-IS and after subtraction of background fluorescence. The distance of the centrosome to the IS and from mitochondria to PCM-1 was calculated using IMARIS 8.4 software (https://imaris.oxinst.com) as described (42). Brightfield channel was used to set the latex bead volume, and the PCM-1 channel was used to generate the MTOC mask. The distance of the MTOC mask to the closer point of the bead volume was measured using the tools “*Intensity Mean*” and “*Distance Channel*”. Quantification of mitochondrial membrane potential (Ψ_m_) was performed using Fiji and ImageJ software to obtain the mean fluorescence intensity (MFI) relative to MitoTracker Orange and Tom-20 at the IS single plane. To obtain the Ψ_m_, the ratio of MitoTracker Orange to Tom-20 was calculated and normalised to the cell area.

### Preparation of glass plates coated with anti-CD3/anti-CD28 antibodies for TIRF microscopy

Plates 35 mm in diameter (glass bottom, 10 mm diameter; No. 1.5 MatTek Corporation, Ashland, MA, USA) were coated with anti-CD3ϵ (10 μg/mL; HIT3a clone) and anti-CD28 (3 μg/mL; CD28.2 clone) monoclonal antibodies previously diluted in bicarbonate buffer (0.1 M NaHCO_3_ and 0.32 M Na_2_CO_3_). One hundred microliters of solution was used per chamber. Before imaging, the plates were washed three times with Hanks’ Balanced Salt Solution (HBSS) and covered with 2 mL of imaging medium (HBSS supplemented with 1.5% FBS and 25 mM HEPES).

### Analysis of CTL degranulation capacity through TIRF microscopy

For CTL degranulation capacity study through total internal reflection fluorescence microscopy (TIRFm), cells were stained with LysoTracker Red DND-99 (1 μM) probe for 1 h at 37°C, washed, and allowed to adhere onto glass-bottom dishes coated with anti-CD3ϵ (10 μg/mL; HIT3a clone) and anti-CD28 (3 μg/mL; CD28.2 clone) antibodies in 2 mL imaging medium. For cells pre-treated with cycloheximide (20 μg/mL for 1 h) or puromycin (50 μg/mL for 1 h), the drug was maintained in the imaging medium during acquisition. The recording was initiated 3 min after cells were plated, and cells were visualised using a Leica AM TIRF MC M system mounted on a Leica DMI6000B microscope coupled to an Andor-DU8285 VP-4094 camera fitted with an HCX PL Apo 100.0×/1.46 oil objective. Images were acquired every 90 ms for 5 min, and the laser penetrance used was 200 nm for the red laser channel (561 nm). Synchronisation was performed using the accompanying Leica software, and images were processed and analysed using IMARIS 8.4 software (https://imaris.oxinst.com) for the number of granules per cell, displacement of granules, and duration of granule tracking (43).

### Mitochondrial mass and lytic granules analysis by flow cytometry

Mitochondrial mass was assessed in cells labelled with nonyl acridine orange (NAO; 20 nM) for 20 min at 37°C. Content in lytic granules was determined in cells by staining with LysoTracker Red DND-99 (1 μM) probe for 1 h at 37°C. Cells were then washed with complete media and resuspended in 200 µL of FACS buffer [HBSS, human γ-globulin (50 µg/mL), 2% BSA, and 1 mM EDTA] for flow cytometry acquisition. Mean fluorescence intensity and geometric mean of staining were acquired using an LSRFortessa flow cytometer (BD Biosciences) and analysed using FlowJo Software (v10.7). The gating strategies are shown in **Supplementary Figure 3**.

### TCR activation and CD28 co-stimulation for Western blotting analysis

For T-cell activation analysis, 1 × 10^6^ CTLs were resuspended in 100 μL of complete media; treated with cycloheximide (20 μg/mL), puromycin (50 μg/mL), or vehicle for 1 h at 37°C; and then stimulated with 10 μL αCD3/αCD28 antibodies (ImmunoCult Human CD3/CD28 T Cell Activator) for the times indicated. For sodium dodecyl sulfate–polyacrylamide gel electrophoresis (SDS-PAGE), 1 × 10^6^ CTLs were lysed in 50 μL of 20 mM Tris-HCl (pH 7.5) containing 1% NP-40, 0.2% Triton X-100, 150 mM NaCl, 2 mM EDTA, and 1.5 mM MgCl_2_ with phosphatase and protease inhibitor cocktails (Sigma-Aldrich) for 30 min at 4°C. Then, samples were processed for electrophoresis in 8% polyacrylamide gels, transferred to nitrocellulose membranes, and subjected to Western blotting. Membranes were incubated with primary antibodies overnight and with peroxidase-labelled secondary antibodies for 1 h at R/T. Signal detection was performed using a chemiluminescence imaging system Amersham 880 (40). Densitometry analysis and quantification of the detected bands in blots were performed using the accompanying software Image Gauge (Fujifilm Inc., Tokyo, Japan). The background was subtracted, and arbitrary unit per pixel was normalised to control samples, not stimulated. For fluorescent Western blotting, IRDye 680 goat anti-rabbit and IRDye 800 goat anti-mouse (Li-Cor Biosciences, Lincoln, NE, USA) were also incubated for 1 h at R/T (44). The fluorescence signal was quantified using an Odyssey Infrared Imager (Li-Cor Biosciences), and densitometry analysis was performed using the Image Studio software (v5.2). Values were analysed using GraphPad software (v8.0) for statistical significance.

### *In vitro* analysis of CD8^+^ T cells functionality by flow cytometry

In 100 μL of complete media, 3 × 10^5^ CTLs per condition were stimulated with 10 μL ImmunoCult Human CD3/CD28 T Cell Activator (StemCell Technologies) and treated with cycloheximide (20 μg/mL), puromycin (50 µg/mL), or vehicle for 1 h at 37°C and 5% CO_2_. Cells unstimulated or stimulated with 0.05 µg/mL phorbol myristate acetate (PMA) (Merck, Darmstadt, Germany) and 0.25 µg/mL ionomycin (PeproTech, Cranbury, NJ, USA) were prepared in parallel as controls (**Supplementary Figure 4B**). Then, 5 µg/mL monensin (Sigma) and CD107a-APC (1:200; BioLegend) antibodies were added in 100 μL of complete media for 4 h at 37°C and 5% CO_2_. Cells were then stained with Ghost dye Red 780 Viability Dye (1:1,000, Tonbo Biosciences) and surface fluorophore-labelled primary antibodies for 20 min on ice. Cells were fixed and permeabilised with fixation and permeabilisation buffer (BioLegend) for 30 min, stained with intracellular fluorophore-labelled primary antibodies for 20 min on ice, and then washed with permeabilisation wash buffer (BioLegend). Finally, cells were resuspended in 200 µL of FACS buffer [HBSS, human γ-globulin (50 µg/mL), 2% BSA, and 1 mM EDTA] for flow cytometry acquisition in a FACSCanto II Analyser Cytometer (405-nm violet laser, 488-nm solid-state blue laser, and 633-nm He-Ne) (BD Biosciences) and analysed using FlowJo Software (v10.7). The gating strategies are shown in **Supplementary Figure 4A**.

### CD8^+^ T-cell viability determination by flow cytometry

To study the impact of cycloheximide and puromycin treatment on the viability of CTLs, 2 × 10^5^ cells were treated with CTRL, CHX (20 μg/mL), or PURO (50 µg/mL) for 1 h. Subsequently, CTLs were activated or not with 20 µL of ImmunoCult Human CD3/CD28 T Cell Activator for 30 min. Then, cells were stained with Ghost dye Violet 510 Viability Dye (1:1,000, Tonbo Biosciences) in 100 µL of phosphate-buffered saline (PBS) for 30 min at 4°C, with anti-CD8-PerCP/Cy5.5 (1:200) in 50 µL of FACS buffer for 20 min at 4°C and with APC-conjugated Annexin V (1:50, BioLegend) in 100 µL of Annexin V Binding Buffer (BioLegend) for 15 min at R/T, in accordance with the manufacturer’s instructions. Data were acquired using a FACSCanto II Analyser Cytometer (405-nm violet laser, 488-nm solid-state blue laser, and 633-nm He-Ne) (BD Biosciences) and analysed using FlowJo Software (v10.7). The gating strategy is shown in **Supplementary Figure 1A**.

### Protein translation monitored by ^35^S Met/Cys metabolic labelling

For ^35^S Met/Cys labelling, 1 × 10^6^ CTLs per condition were incubated in cysteine/methionine-free medium (Sigma-Aldrich) for 30 min at 37°C, followed by incubation in cysteine/methionine-free medium + dialysed FBS containing 50 μCi/mL ^35^S-labelled cysteine/methionine (Revvity) for 1 h. For the puromycin/cycloheximide assay, cells were pre-treated with puromycin (50 μg/mL) or cycloheximide (20 μg/mL) in parallel to the radioactive labelling, and CTLs were activated for the indicated times. Cells were then harvested and washed with cysteine/methionine-free medium, and total lysates were obtained (Tris-HCl, pH 7.5, 1% NP-40, 0.2% Tx-100, 140 mM NaCl, 1 mM EDTA, 1.5 mM MgCl_2_, and inhibitors of proteases and phosphatases). Cell lysates were analysed using SDS-PAGE and autoradiography.

### CMV-specific CD8^+^ T-cell killing of monocytes

To demonstrate the killing capacity of CD8^+^ T cells, an assay of monocyte killing by CD8^+^ T cells was performed using a cytomegalovirus (CMV)-specific model. For this, PBMCs were isolated from healthy donors phenotyped for CMV and treated with CHX (20 μg/mL), PURO (50 µg/mL), or CTRL. Subsequently, human CMV peptide pools (1.25 µg/mL, PepMix, JPT Peptide Technologies, Berlin, Germany) were added for 18 h. A pool of peptides spanning actin was used as a negative control. Stimulating αCD3/αCD28 antibodies (20 microliters; ImmunoCult Human CD3/CD28 T Cell Activator) were used as a positive control. Subsequently, cells were stained with Ghost dye Red 780 Viability Dye (1:1,000, Tonbo Biosciences) in 100 µL of PBS for 30 min at 4°C. Then, cells were stained with surface fluorophore-labelled primary antibodies for 20 min at 4°C for CMV-specific activation (CD69-V450, CD3-PerCP/Cy5.5, and CD8-V500) or killing (CD3-PerCP/Cy5.5, CD8-V500, and CD14-PE) flow cytometry panels and analysed using FlowJo Software (v10.7). The gating strategy is shown in **Supplementary Figure 6**.

### IFN-γ detection by ELISPOT

To determine the effect of protein translation inhibition in IFN-γ production by PBMCs in response to CMV peptides, cells were resuspended in RPMI 1640 medium supplemented with 10% FBS; treated with CHX (20 μg/mL), PURO (50 µg/mL), or CTRL; and plated at a final density of 1 × 10^5^ cells/well for 16 h at 37°C and 5% CO_2_. The cellular response was assessed using CMV peptide pools (1.25 µg/mL, PepMix, JPT Peptide Technologies). As a negative control, a pool of peptides spanning actin was used. To help with cell stimulation, 1 µg/mL of anti-CD28 antibody (clone 15E8, Miltenyi Biotec, Bergisch Gladbach, Germany) was added to the culture medium. As a positive control, 1 × 10^5^ cells/well were plated in the presence of anti-CD3 (clone CD3–2) monoclonal antibody at 1 µg/mL. Spots were revealed following the manufacturer’s protocol (Mabtech, Stockholm, Sweden). Enzyme-linked immunosorbent spot (ELISPOT) plates were analysed using an AID classic ELISPOT reader (AID Autoimmun Diagnostika GmbH).

### IFN-γ detection by ELISA

To study the capacity of CTLs to produce IFN-γ after TCR and CD28 co-stimulation, 0.5 × 10^6^ cells per condition were treated with CHX (20 μg/mL), PURO (50 µg/mL), or CTRL for 1 h and then stimulated or not with 10 μL of αCD3/αCD28 antibodies (ImmunoCult Human CD3/CD28 T Cell Activator) for 30 min. Then, supernatant cultures were collected, and cells were lysed in 50 μL of 20 mM Tris-HCl (pH 7.5) containing 1% NP-40, 0.2% Triton X-100, 150 mM NaCl, 2 mM EDTA, and 1.5 mM MgCl_2_ with phosphatase and protease inhibitor cocktails (Sigma-Aldrich) for 30 min at 4°C. IFN-γ production was determined in cell lysates and supernatant cultures using the Human IFN-γ ELISA Set (Diaclone, Besançon, France) following the manufacturer’s instructions.

### Mitochondrial respiration and glycolysis assays

An XF96 extracellular flux analyser (Seahorse Bioscience, North Billerica, MA, USA; XF96 FluxPak Agilent Technologies) was used to measure the oxygen consumption rate (OCR) and extracellular acidification rate (ECAR) (25). For the glucose-based mitostress test, the use of glucose was measured in cells pre-treated with vehicle, cycloheximide (20 μg/mL), and puromycin (50 µg/mL) for 1 h in basal conditions or after stimulation with 10 μL ImmunoCult Human CD3/CD28 T Cell Activator (StemCell Technologies). Cells were cultured with Dulbecco’s modified Eagle medium (DMEM) (D5030, Sigma Aldrich) supplemented with 1 mM sodium pyruvate, 1 mM l-glutamine, and 25 mM glucose and were seeded at 0.5 × 10^6^ per well in culture plates pre-coated with poly-l-Lys. Each plate included three independent donors and five technical replicates. Drugs were injected as follows: oligomycin (1.8 μM), CCCP (2 μM), rotenone (1 μM), and antimycin A (1 μM). Three consecutive mix and measure steps were performed for resting conditions and after each injection (3 min each). For the glycolysis stress assay, cells were cultured with DMEM supplemented with 2 mM l-glutamine and seeded as before (three biological and five technical replicates per plate). Injections were as follows: glucose (10 mM), oligomycin (1.8 μM), and 2-deoxyglucose (2-DG; 50 mM). Mix and measure steps were as before. A linear mixed model performed in R (v4.2.1) (https://cran.r-project.org) was used for statistical analysis of OCR and ECAR (24, 25).

### Isolation of mitochondria: Western blot and flow cytometry analysis

For analysis, 5 × 10^6^ CTLs per condition were pre-treated with vehicle, cycloheximide (20 μg/mL), and puromycin (50 µg/mL) for 1 h at 37°C and 5% CO_2_ and then stimulated or not for 15 min with 20 μL αCD3/αCD28 antibodies (ImmunoCult Human CD3/CD28 T cell Activator). Mitochondria were isolated with a Miltenyi isolation kit for human mitochondria following the manufacturer’s instructions. Isolated mitochondria were resuspended in 25 μL radioimmunoprecipitation assay buffer with phosphatase and protease inhibitors (Sigma-Aldrich), then sonicated for 1 h and processed for electrophoresis in 10% polyacrylamide gels, transferred to nitrocellulose membranes, and subjected to Western blotting. For puromycin determination in mitochondria by flow cytometry, isolated mitochondria were stained with Alexa Fluor 488 anti-puromycin antibody (clone 2A4, BioLegend) for 1 h at 4°C and then resuspended in FACS buffer for flow cytometry acquisition using a FACSCanto II Analyser Cytometer (405-nm violet laser, 488-nm solid-state blue laser, and 633-nm He-Ne) (BD Biosciences) and analysed using FlowJo Software (v10.7). The gating strategy is shown in **Supplementary Figure 2A**.

### Statistical analysis

Statistical analyses were performed using PRISM8 (GraphPad software). Normality tests were performed, and when comparing two samples, Student’s t-test or the Mann–Whitney test was performed according to normality or not, respectively. When comparing three or more samples, one-way or two-way ANOVA was used. Specific details of each analysis are described in the figure legends. Significant differences were considered when p < 0.05 (*). ** indicates p < 0.01, *** indicates p < 0.001, and **** indicates p < 0.0001.

## Results

### Eukaryotic protein translation inhibition affects tubulin cytoskeleton dynamics and TCR signalling of human cytotoxic T lymphocytes

To investigate the potential impact of cytosolic protein translation on the tubulin cytoskeleton, human CTLs were exposed to CTRL, CHX (20 μg/mL), or PURO (50 μg/mL) for 1 h at 37°C. These are inhibitors of the elongation of polypeptides during translation in 80S ribosomes, with a different site of action in the 60S large subunit, blocking translation (CHX) (45) or interrupting the polypeptide synthesis with production of puromycylated peptides (PURO) (46). Indeed, PURO has also been used to inhibit mitochondrial 55S ribosomes *in vitro* (47). To test the potential effect on the viability of CTLs of the compounds, CTLs were untreated or pre-treated with CHX or PURO for 1 h, and cells were left unstimulated or activated with anti-CD3 and anti-CD28 antibodies. Activation via TCR and co-stimulation for 1 h decreased the percentage of live cells in (CTRL, 76.09% vs. 66.88%; CHX, 73.80% vs. 62.64%; and PURO, 73.4% vs. 63.39%, unstimulated vs. stimulated, respectively) (**Supplementary Figure 1A**), as previously described (48). Cell viability was found to be similar across all treatments (**Supplementary Figure 1A**). Additionally, to test the inhibitory effect of CHX and PURO in TCR-induced early *de novo* protein synthesis in CTLs, their activity was monitored through metabolic labelling with ^35^S Met/Cys. The incorporation of ^35^S in total proteins increased at 15 and 30 min after TCR activation and co-stimulation in CTRL. PURO completely abrogated ^35^S Met/Cys incorporation in resting and stimulated cells, whereas CHX showed a more moderate action, with maximal effect at 30 min of stimulation (**Supplementary Figure 1B**).

**Figure 1:**
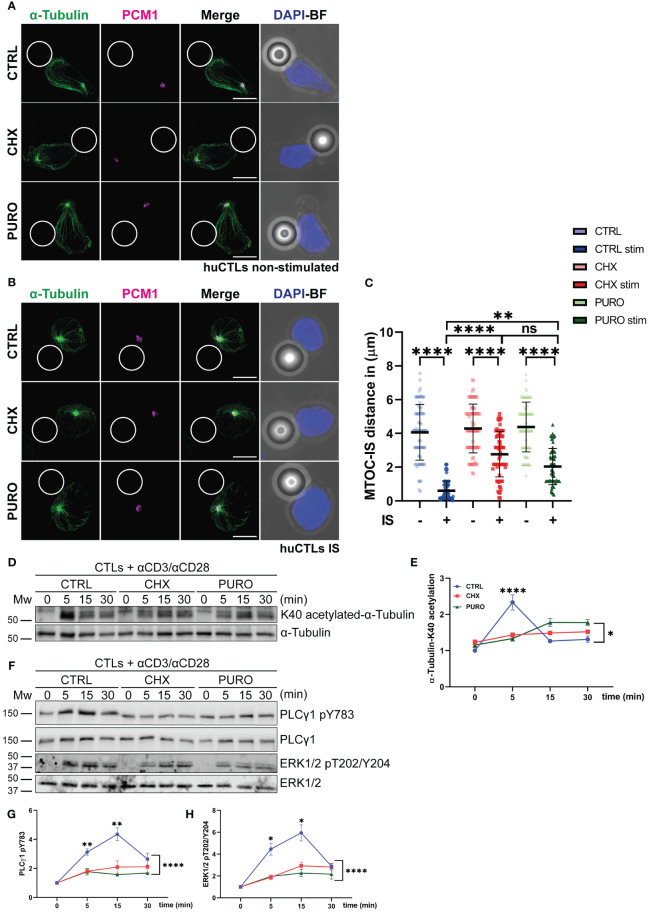
Cytosolic protein translation supports tubulin cytoskeleton reorganisation in human CD8^+^ cytotoxic T lymphocytes during T-cell receptor (TCR) and CD28 activation. **(A, B)** Fluorescence images of human cytotoxic T lymphocytes (CTLs) treated with vehicle (CTRL), cycloheximide (CHX; 20 μg/mL), or puromycin (PURO; 50 μg/mL) for 1 h and conjugated with **(A)** control or **(B)** stimulating beads. Green, α-tubulin; magenta, PCM1; blue, DAPI; BF, brightfield. Maximal projections are shown. Bar, 5 μm. **(C)** Graph showing quantification of microtubule-organising centre (MTOC) distance to the immunological synapse (IS). Data are mean ± SD; CTRL(−), n = 74; CTRL(+), n = 85; CHX(−), n = 74; CHX(+), n = 90; PURO(−), n = 74; and PURO(+), n = 80, cells analysed from six healthy donors; one-way ANOVA. **, p < 0.01; ****, p < 0.0001; ns, non-significant. **(D)** K40-α-tubulin acetylation in CTLs pre-treated as in panel A were stimulated or not with αCD3/αCD28 tetramers at indicated times. **(E)** Graph showing densitometric quantification of acetylated K40-α-tubulin normalised to total α-tubulin and referenced to non-stimulated CTRL. Data are mean ± SEM; n = 3; two-way ANOVA. *, p < 0.05. **(F)** Phosphorylation of PLCγ1 (pY783) and Erk1/2 (pT202/Y204) in CTLs pre-treated as in panel A and stimulated or not with αCD3/αCD28 tetramers at indicated times. **(G, H)** Graphs showing quantification of densitometries of **(G)** PLCγ1 (pY783) and **(H)** Erk1/2 (pT202/Y204) normalised to PLCγ1 and Erk1/2 and referenced to non-stimulated CTRL. Data are mean ± SEM; **(G)**, n = 7; **(H)**, n = 4; two-way ANOVA. *, p < 0.05; **, p < 0.01; ****, p < 0.0001. See also **Supplementary Figure 1**.

CTLs were conjugated with control (IgG-coated) or activating beads coated with anti-CD3 and anti-CD28 monoclonal antibodies to simulate non-productive and synaptic contacts, respectively. Cells were then stained for α-tubulin and pericentrin-associated protein-1 (PCM-1) to observe the tubulin cytoskeleton and the centrosomal area (**Figure 1**). PCM-1 is required for the assembly of several centrosomal proteins such as pericentrin (49). In unstimulated CTLs, the centrosome showed a heterogeneous distribution in control cells and those treated with CHX or PURO (**Figure 1A**). Upon contact with activating beads, control cells successfully translocated the centrosome to the bead contact zone (**Figures 1B, C**). In contrast, cells treated with CHX or PURO exhibited an altered polarisation of the centrosome towards the synaptic area, showing an increased distance to the centrosome than control cells (**Figures 1B, C**). To further investigate the effect of protein synthesis inhibition on tubulin cytoskeleton dynamics, CTRL, CHX, or PURO CTLs were activated with anti-CD3 and anti-CD28 antibodies for 5 min, 15 min, and 30 min, and tubulin acetylation (K40-acetylation-α-tubulin), a marker of MT stability (50), was determined by Western blotting (**Figure 1D**). After 5 min of TCR activation, CTRL exhibited an increase in tubulin acetylation corresponding to the timing of established centrosome translocation (51) and organisation of the tubulin network (52), while cells treated with CHX or PURO did not respond to activation by increasing tubulin acetylation as control cells (**Figure 1E**). These results point to an altered tubulin dynamics in CTLs upon inhibition of protein translation on 80S ribosomes. Due to the significance of tubulin cytoskeleton dynamics and rearrangement in T-cell activation, we investigated early downstream TCR signalling by Western blotting to determine the phosphorylation of PLCγ1 (pY783) and Erk1/2 (pT202/Y204) following activation with anti-CD3 and anti-CD28 antibodies of CTRL-, CHX-, or PURO-treated CTLs (**Figure 1F**). Treatment with CHX or PURO resulted also in reduced phosphorylation of PLCγ1 (**Figure 1G**) and Erk1/2 (**Figure 1H**), indicating that protein synthesis inhibition impacts downstream TCR signalling. Since tubulin dynamics is required for complete PLCγ1 activation due to intracellular transport of vesicles upon T-cell activation (52), these data suggest that inhibition of protein translation on 80S ribosomes affects TCR downstream signalling pathways by preventing organisation and dynamics of the tubulin cytoskeleton during T-cell activation. Indeed, inhibition of 55S mitochondrial ribosomes could also increase the effect in CTLs after puromycin treatment. To assess whether PURO could affect mitochondria, they were isolated from CTRL, CHX, or PURO CTLs. Mitochondria from puromycin-treated cells were positive for puromycin labelling by flow cytometry (**Supplementary Figure 2A**). These results point to a reinforcement of the puromycin effect due to the potential dual inhibition of cytosolic and mitochondrial ribosomes.

### Mitochondrial network organisation in stimulated CD8 cytotoxic T lymphocytes is affected by the inhibition of cytosolic protein translation

**Figure 2:**
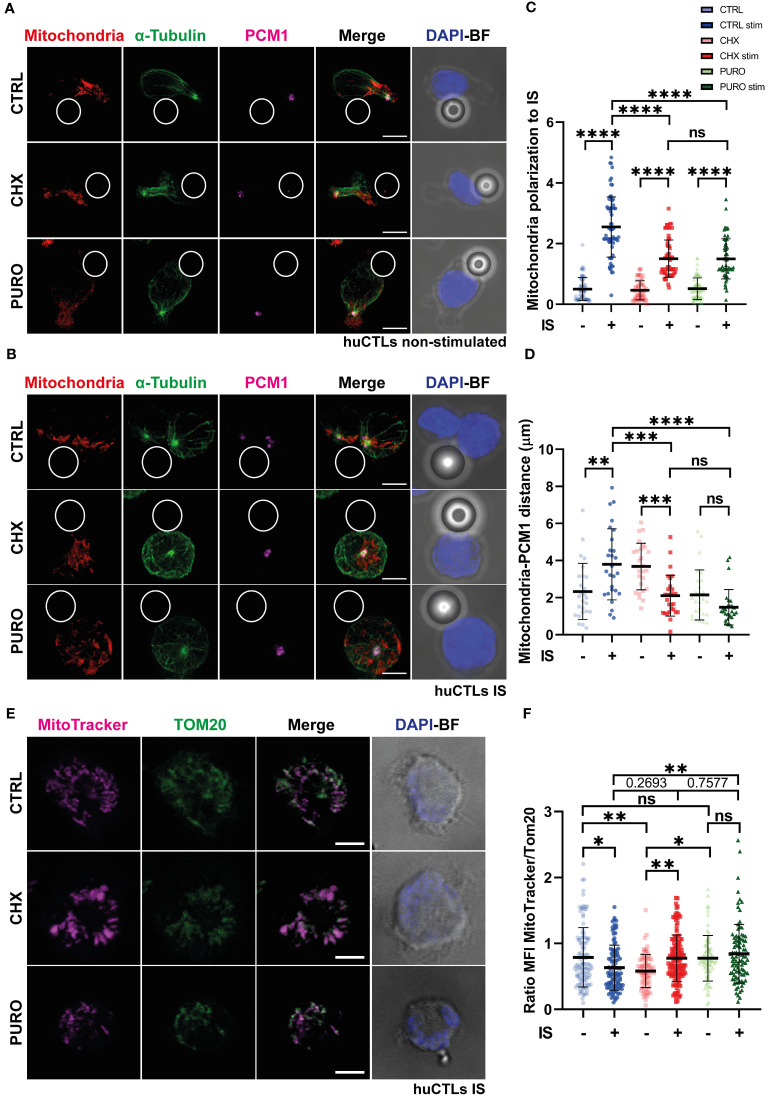
Mitochondrial network reorganisation in human CD8^+^ cytotoxic T lymphocytes requires active cytosolic protein translation during T-cell receptor (TCR)-driven asymmetry. **(A, B)** Fluorescence images of human cytotoxic T lymphocytes (CTLs) treated with vehicle (CTRL), cycloheximide (CHX; 20 μg/mL), or puromycin (PURO; 50 μg/mL) for 1 h and conjugated with **(A)** control or **(B)** stimulating beads to establish immunological synapse (IS). Red, MitoTracker Orange; green, α-tubulin; magenta, PCM1; blue, DAPI; BF, brightfield. Maximal projections are shown. Bar, 5 μm. **(C)** Graph showing quantification of mitochondrial polarisation to the IS. Data are mean ± SD; CTRL(−), n = 63; CTRL(+), n = 65; CHX(−), n = 62; CHX(+), n = 64; PURO(−), n = 64; and PURO(+), n = 64, cells analysed from six healthy donors; one-way ANOVA. ****, p < 0.0001; ns, non-significant. **(D)** Graph showing quantification of mitochondria distance to PCM-1. Data are mean ± SD; CTRL(−), n = 26; CTRL(+), n = 27; CHX(−), n = 27; CHX(+), n = 29; PURO(−), n = 27; and PURO(+), n = 28, cells analysed from six healthy donors; one-way ANOVA. *, p < 0.05; **, p < 0.01; ***, p < 0.001; ****, p < 0.0001; ns, non-significant. **(E)** Fluorescence images of human cytotoxic T lymphocytes (CTLs) treated as in panel A and settled over stimulatory surfaces coated with anti-CD3 and anti-CD28 monoclonal antibodies. Magenta, MitoTracker Orange; green, Tom-20; blue, DAPI; BF, brightfield. Images are single planes of the IS plane. Bar, 5 μm. **(F)** Graph showing quantification of the ratio between MitoTracker Orange and Tom-20 mean fluorescence intensity (MFI). Data are mean ± SD; CTRL(−), n = 113; CTRL(+), n = 102; CHX(−), n = 77; CHX(+), n = 138; PURO(−), n = 77; and PURO(+), n = 96, cells analysed from six healthy donors; one-way ANOVA. *, p < 0.05; **, p < 0.01; ns, non-significant. See also **Supplementary Figure 2**.

Due to the crucial role of tubulin cytoskeleton dynamics in organising the mitochondrial network during T-cell activation in human peripheral blood lymphocytes (PBLs) (25), CTLs were subjected to treatment with CTRL, CHX, or PURO for 1 h; then conjugated with control beads; and stained for mitochondria, α-tubulin, and PCM-1. Under resting conditions in the absence of TCR stimulation, mitochondria were mainly distributed in the uropod, near the centrosome in all CTLs (**Figure 2A**). Upon conjugation of CTLs with activating beads, CTRL correctly polarised their mitochondria to the IS accompanying the centrosome (**Figures 2B, C**). In contrast, cells treated with CHX and PURO exhibited a defect in mitochondrial polarisation to the synaptic area (**Figures 2B, C**). In PURO and CHX CTLs, mitochondria remained around the non-translocated centrosome in the centre of the cell. Indeed, the relative localisation of mitochondria to the centrosome was different in CHX- and PURO-treated cells, with PURO mitochondria closer to the centrosome (**Figures 2B–D**). However, in unstimulated CHX-treated cells, two clusters of mitochondria were observed—one in the centrosome and the other outside the uropod—in the mid-zone of the cell, although the mass of mitochondria was in general closer to the centrosome than in CTRL and PURO CTLs (**Figures 2A, D**). These data suggest that CHX and PURO have distinct effects on CTLs, which may be attributed to differences in their mechanisms of action such as a combined effect of PURO in cytosolic and mitochondrial ribosomes.

To investigate mitochondrial activity during synaptic contacts, CTLs were labelled with a MitoTracker Orange probe to stain mitochondria according to their mitochondrial membrane potential (Ψ_m_), treated, and settled over poly-l-Lys-coated coverslips (**Supplementary Figure 2B**), or stimulatory surfaces coated with anti-CD3 and anti-CD28 monoclonal antibodies (**Figure 2E**). MFI of Ψ_m_ was normalised to Tom-20 (translocase of the outer mitochondrial membrane-20) MFI (**Figure 2F**). This Ψ_m_/mitochondrial mass ratio is crucial for CD4^+^ T-cell activation (24). In control cells, TCR activation and co-stimulation for 30 min resulted in a drop in mitochondrial membrane potential, as shown by the decrease in the Ψ_m_/mitochondrial mass (**Figure 2F**), probably due to the production of ATP using the flux of protons through the mitochondrial F0/F1 ATPase. CTLs treated with CHX showed a significant increase in the Ψ_m_ mitochondrial mass ratio after activation, whereas in CTLs treated with PURO, there was a slight increase (**Figure 2F**), which suggests that mitochondria in CHX and PURO are not using this potential to produce ATP through the F0/F1 ATPase. These results show that inhibition of cytosolic protein synthesis impacts the distribution and activity of mitochondria upon TCR stimulation and co-stimulation in human CTLs.

### Eukaryotic protein translation is required for mitochondrial respiration in human cytotoxic T lymphocytes

**Figure 3:**
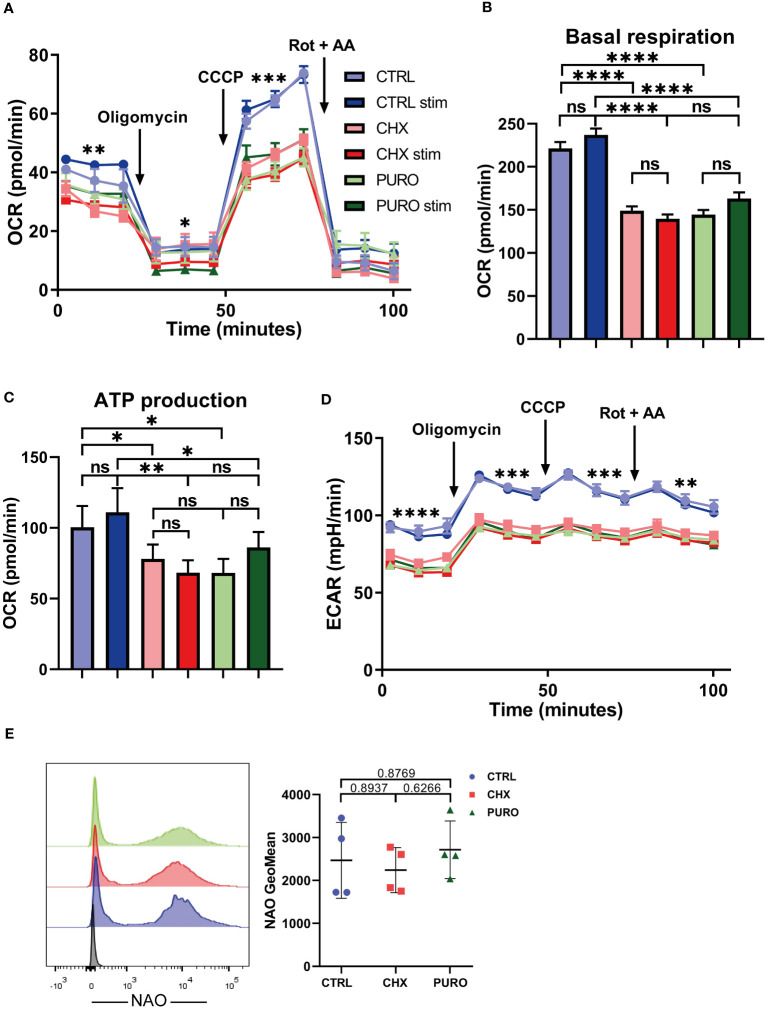
Protein translation on 80S ribosomes is required for mitochondrial respiration in human CD8^+^ cytotoxic T lymphocytes. **(A)** Mitochondrial oxygen consumption rate (OCR) in resting and stimulated human cytotoxic T lymphocytes (CTLs) treated with vehicle (CTRL), cycloheximide (CHX; 20 μg/mL), or puromycin (PURO; 50 μg/mL) for 1 h. Stimulation was with αCD3/αCD28 tetramers. Oligomycin, CCCP, and rotenone plus antimycin A were injected as indicated. **(B, C)** Graphs show **(B)** basal respiration and **(C)** ATP production. **(D)** Extracellular acidification rate (ECAR) from experiment shown in panel **(A)** Data are mean ± SEM from five technical replicates from six healthy donors. **(A, D)** A representative experiment out of six. Linear mixed model was used to analyse differences in **(A)** OCR and **(D)** ECAR and one-way ANOVA test for **(B)** basal respiration and **(C)** ATP production. *, p < 0.05; **, p < 0.01; ***, p < 0.001; ****, p < 0.0001; ns, non-significant. **(E)** Mitochondrial mass of CTLs treated as in panel **(A)** Graph shows the geometric mean (GeoMean) of nonyl acridine orange (NAO) fluorescence. Graph showing mean ± SD; n = 4. Unpaired t-test; ns, non-significant. See also **Supplementary Figure 3** for gating strategy.

To further address the relevance of cytosolic protein translation on mitochondrial functionality, CTLs were subjected to a mitostress test using glucose as a unique external energy source, and OCR and ECAR were measured. CTRL, CHX, or PURO CTLs were left unstimulated or stimulated with anti-CD3 and anti-CD28 monoclonal antibodies (**Figure 3**). OCR curve (**Figure 3A**) indicated that control CTLs showed higher OCR using glucose compared to CTLs treated with CHX or PURO. Additionally, CHX- or PURO-treated cells did not respond to stimulation, unlike CTRL-treated CTLs, which showed a slight but non-significant increase in different parameters. Basal respiration (**Figure 3B**) and ATP production (**Figure 3C**) were reduced in CTLs treated with CHX and PURO, supporting the observed increase in the Ψ_m_/mitochondrial mass ratio (**Figure 2E**). Maximal respiration was also decreased in CTLs treated with CHX or PURO. Control CTLs also showed a higher ECAR in this assay (**Figure 3D**), suggesting a potential defect in glycolysis due to decreased lactic acid production. These data suggest that mitochondria in CHX- and PURO-treated CTLs are less efficient in using acetyl-CoA derived from glucose.

To ensure that the differences observed in mitochondrial respiration were not caused by a reduction in mitochondrial content in CTLs treated with CHX or PURO, mitochondrial mass was assessed by staining cells with NAO, a mitochondrial probe that binds to cardiolipin in mitochondria, independently of the Ψ_m_. No significant differences in mitochondrial mass were observed (**Figure 3E**).

### Inhibition of eukaryotic translation affects glycolytic response and Akt/mTOR/S6 signalling in human CTLs

**Figure 4:**
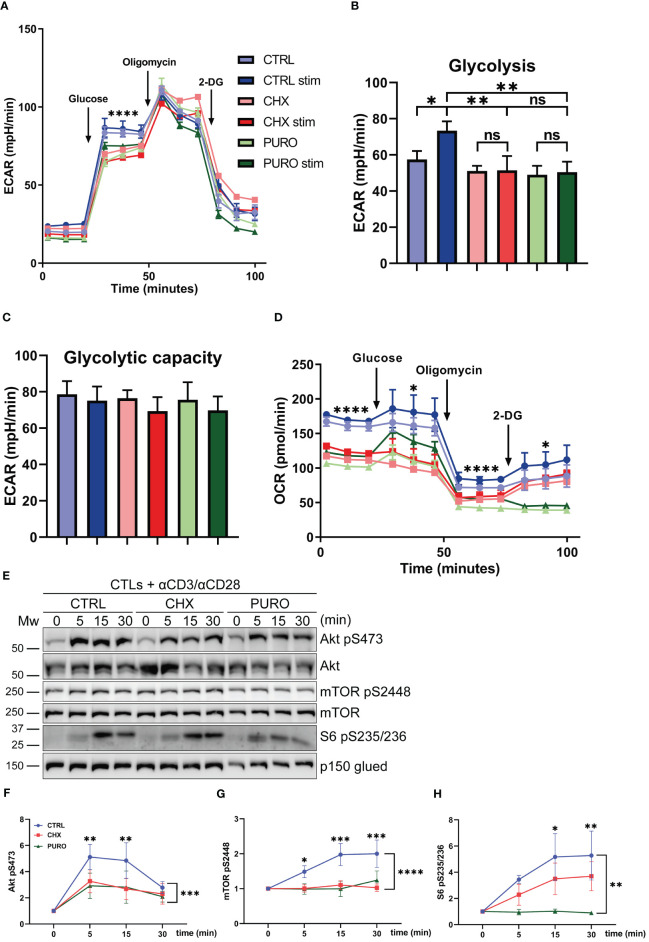
Glycolytic response during T-cell receptor (TCR) activation requires active cytosolic protein translation in human CD8^+^ cytotoxic T lymphocytes. **(A)** Glycolytic response estimated by extracellular acidification rate (ECAR) in resting or stimulated human cytotoxic T lymphocytes (CTLs) treated with vehicle (CTRL), cycloheximide (CHX; 20 μg/mL), or puromycin (PURO; 50 μg/mL) for 1 h. Stimulation was with αCD3/αCD28 tetramers. Glucose, oligomycin, and 2-deoxyglucose (2-DG) were injected as indicated. **(B, C)** Graphs show the rate of **(B)** glycolysis and **(C)** glycolytic capacity. Data are mean ± SEM from five technical replicates from six healthy donors. **(D)** Oxygen consumption rate (OCR) from the experiment in panel **(A)**. **(A, D)** A representative experiment out of six. Linear mixed model was used to analyse differences in **(A)** ECAR and **(D)** OCR and one-way ANOVA test for **(B)** glycolysis and **(C)** glycolytic capacity. *, p < 0.05; **, p < 0.01; ****, p < 0.0001; ns, non-significant. **(E)** Phosphorylation of Akt (S473), mTOR (S2448), and S6 (S235/S236) in CTLs pre-treated as in panel A and stimulated with αCD3/αCD28 tetramers at indicated times. Graphs showing quantification of densitometries of **(F)** Akt (S473), **(G)** mTOR (S2448), and **(H)** S6 (S235/S236) blots; data are normalised to p150-Glued and referenced to non-stimulated CTRL. Data are mean ± SEM; **(F)**, n = 4 **(G)**, n = 5 **(H)**, n = 4; two-way ANOVA. *, p < 0.05; **, p < 0.01; *** p < 0.001; **** p < 0.0001.

To ascertain whether glycolysis is affected by protein translation inhibition on 80S ribosomes, CTLs were subjected to a glycolysis stress test, and ECAR and OCR were measured (**Figure 4**). After glucose injection, the ECAR curve showed a significant decrease in CHX- and PURO-treated CTLs (**Figure 4A**). This indicates lower lactic acid production as a result of defective glycolysis. In contrast to CTRL-treated CTLs, when CTLs were treated with CHX or PURO, they were unable to respond to stimulation (**Figure 4B**), indicating that the switch to a higher glycolytic status in CTLs upon TCR activation is prevented by CHX and PURO. After the inhibition of the F0/F1 ATPase with oligomycin to maximise glycolysis, no differences were observed between CTRL-treated CTLs and those treated with CHX or PURO, resulting in similar glycolytic capacity (**Figure 4C**). CHX or PURO treatment promoted lower OCR during this test (**Figure 4D**) as in the mitostress test (**Figure 3**), confirming the altered mitochondrial functionality by inhibition of cytosolic translation during early TCR activation. These effects may be partly due to defective dynamics of the cytoskeleton and mitochondria, not allowing the correct asymmetric positioning of organelles in CTLs for full functionality.

Considering the differences observed in the metabolic fate of CTLs after cytosolic protein synthesis inhibition, the Akt/mTOR/S6 signalling pathway, which regulates T-lymphocyte metabolism and protein synthesis, among other processes (36), was investigated. CTRL-, CHX-, and PURO-treated CTLs were stimulated with anti-CD3 and anti-CD28 antibodies for 5 min, 15 min, and 30 min, and the phosphorylation of Akt (S473), mTOR (S2448), and S6 (S235/S236) was determined by Western blotting (**Figure 4E**). The reduced phosphorylation kinetics of S473 Akt (**Figure 4F**), S2448 mTOR (**Figure 4G**), and S235/S236 S6 (**Figure 4H**) in CHX- and PURO-treated CTLs suggested that eukaryotic protein translation is required for the correct activation of these pathways. These data indicate a lower metabolic capacity of CTLs in response to TCR activation in the absence of cytosolic translation.

### Cytosolic protein translation regulates degranulation capacity and effector function of human CTLs

**Figure 5:**
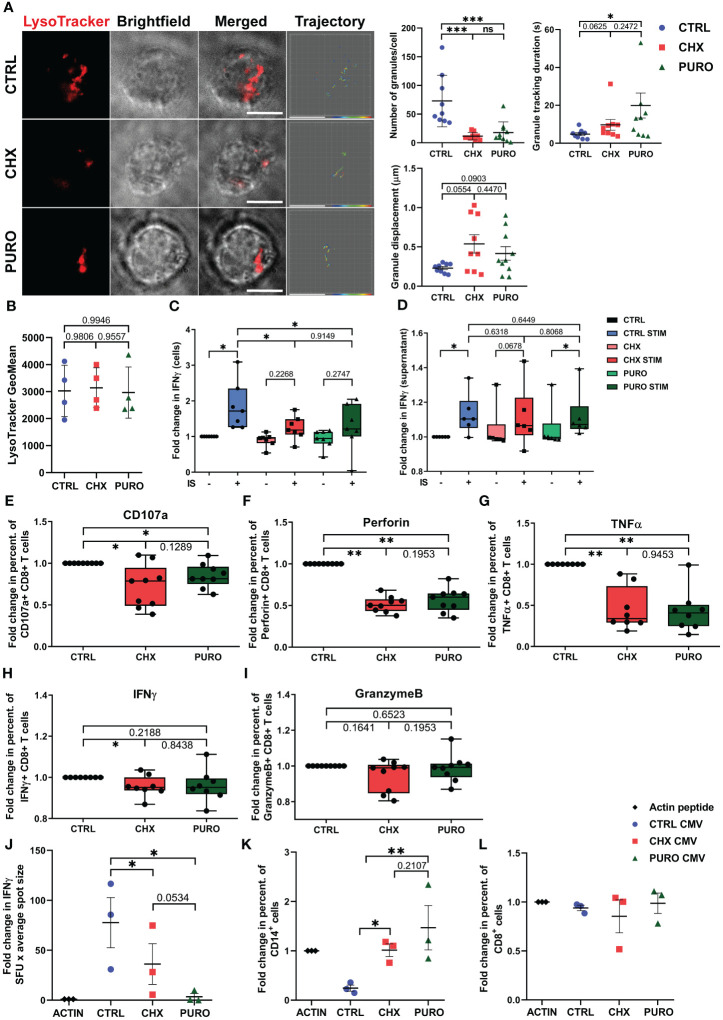
Polyfunctional capacity of human CD8^+^ cytotoxic T lymphocytes requires cytosolic protein translation during early T-cell activation. **(A)** Representative fluorescence images from total internal reflection fluorescence microscopy (TIRFm) of LysoTracker-Red DND-99-loaded cytotoxic T lymphocytes (CTLs). Brightfield images and granule trajectories are also provided. CTLs were treated with vehicle (CTRL), cycloheximide (CHX; 20 μg/mL), or puromycin (PURO; 50 μg/mL) for 1 h and then settled onto glass-bottom chambers coated with anti-CD3 (Hit3a clone) and anti-CD28 (CD28.2 clone) monoclonal antibodies. Video recording was initiated upon cell adhesion (37°C and 5% CO_2_). Images were acquired for 5 min every 90 ms. Individual granule trajectories were obtained by fluorescent tracking of puncta. Graphs showing quantification of lytic granule parameters at the immune-synapse-like structure performed for each cell: number of granules/cell, granule tracking duration in seconds, and granule displacement in µm. Data of mean ± SD; CTRL, n = 9; CHX, n = 9; and PURO, n = 10 cells analysed from four independent healthy donors; unpaired t-test; *, p < 0.05; ***, p < 0.001; ns, non-significant. **(B)** Flow cytometry analysis of LysoTracker-Red fluorescence in vehicle (CTRL), cycloheximide (CHX; 20 μg/mL), or puromycin (PURO; 50 μg/mL) pre-treated CTLs and loaded with LysoTracker-Red DND-99 (1 μM) for 1 h. Graph shows the geometric mean (GeoMean) of LysoTracker-Red fluorescence. Graph showing mean SD; n = 4; unpaired t-test. See also **Supplementary Figure 3B** for gating strategy. **(C, D)** IFN-γ production in **(C)** CTLs and **(D)** culture supernatants treated as in panel **(B)** Graph showing box and whiskers (median plus min and max) from n = 6 healthy donors; two-tailed non-parametric Wilcoxon test. **(E–I)** Fold change in proportions of CD8^+^ T cells stimulated with αCD3/αCD28 tetramers and treated with vehicle (CTRL), cycloheximide (CHX; 20 μg/mL), or puromycin (PURO; 50 μg/mL), expressing **(E)** CD107a, **(F)** perforin, **(G)** TNF-α, **(H)** IFN-γ, and **(I)** Granzyme **(B)** Graphs showing box and whiskers (median plus min and max) from n = 8 healthy donors; two-tailed non-parametric Wilcoxon test. *, p < 0.05; **, p < 0.01. **(J–L)** Peripheral blood mononuclear cells (PBMCs) treated with vehicle (CTRL), cycloheximide (CHX; 20 μg/mL), or puromycin (PURO; 50 μg/mL), stimulated with CMV-peptides for 18 h, and assayed for **(J)** IFN-γ production by enzyme-linked immunosorbent spot (ELISPOT). **(K)** Percentage of CD14^+^ live cells and **(L)** percentage of CD8^+^ live cells. Graphs showing mean ± SD; n = 3 healthy donors; two-tailed non-parametric Wilcoxon test. *, p < 0.05; **, p < 0.01. See also **Supplementary Figures 4**, **6** for gating strategies.

To investigate the effect of defective dynamics of MTs in CHX- and PURO-treated CTLs, the dynamics of lytic granules was studied using TIRFm in treated CTLs that were incubated with LysoTracker-Red DND-99 and seeded on glass-bottom chambers coated with anti-CD3 and anti-CD28 monoclonal antibodies (**Figure 5A**). A decrease in the number of granules delivered at the activating surface (i.e., TIRF focal plane) was observed in CHX- and PURO-treated CTLs (**Figure 5A**), indicating defects in their active transport to the plasma membrane. An increase in the duration (**Figure 5A**) and displacement (**Figure 5A**) of the trajectories of lytic granules was observed in CHX- and PURO-treated CTLs. To determine whether these treatments reduced the content in lytic granules compared to control CTLs, cells were monitored by flow cytometry, and non-significant differences were observed (**Figure 5B**). These data indicate that cytosolic translation, regulating microtubule dynamics, is required for correct lytic granule dynamics in TCR-activated CTLs at the active site.

The production and release of IFN-γ in CTLs were assessed in cells (**Figure 5C**) and culture supernatants (**Figure 5D**) through ELISA from CTRL-, CHX-, or PURO-treated CTLs unstimulated or activated with anti-CD3 + anti-CD28 antibodies for 30 min. IFN-γ content in cells largely increased in activated CTRL, which was reversed by treatments (**Figure 5C**), pointing to specific inhibition of *de novo* IFN-γ production. The secreted IFN-γ increases in CTRL-treated CTLs at 30 min of stimulation (**Figure 5D**), and CHX prevents the increase of IFN-γ secretion, although PURO increases at this short time of activation (**Figure 5D**). Stimulated CTRL-treated CTLs showed no difference with PURO- or CHX-treated cells, suggesting that centrosome polarisation or conserved lytic granule dynamics at the IS-like is not required for the secretion of IFN-γ by CTLs at short times. To further investigate the effect of cytosolic protein translation inhibition on the degranulation capacity and effector function of CTLs, CTLs were subjected to degranulation assays by flow cytometry. CTRL-, CHX-, and PURO-treated CTLs were simultaneously treated and stimulated with anti-CD3 and anti-CD28 monoclonal antibodies for 1 h. CD107a-APC antibody was used to monitor degranulation (**Supplementary Figure 4A**). No differences were observed in the percentage of cells expressing CD107a in the membrane in unstimulated or PMA/ionomycin-stimulated CTLs, supporting the correct potential functionality of these CTLs (**Supplementary Figure 4B**). CTLs treated with CHX or PURO and activated via TCR showed a decrease in the percentage of cells expressing CD107a on the membrane, indicating a defect in degranulation capacity after inhibition of cytosolic protein translation (**Figure 5E**). Consistent with previous observations, CTLs treated with CHX or PURO showed a significant decrease in the percentage of cells expressing perforin (**Figure 5F**), a lytic mediator and pro-inflammatory cytokine TNF-α (**Figure 5G**), whereas IFN-γ was significantly decreased in CHX-treated CTLs but only showed a tendency in PURO (**Figure 5H**). No differences were observed in the percentage of CTLs expressing Granzyme B in CHX and PURO compared to CTRL (**Figure 5I**).

To assess the relevance of *de novo* proteins in CTLs in an antigen-specific model, PBMCs from healthy donors were treated with CHX or PURO and activated with CMV-derived peptides for 18 h. The secretion of IFN-γ was then determined by ELISPOT (**Figure 5J**). CHX and PURO treatments reduced the secretion of IFN-γ (**Figure 5J**). In parallel, the potential ability of primary human CD8^+^ T cells to respond against cells presenting specific CMV peptides was investigated using this same CMV model in PBMCs, although we cannot discard the participation of other cells. The percentage of live monocytes (CD14^+^ cells) largely decreased in CTRL PBMCs stimulated with CMV peptides (**Figure 5K**), and CHX and PURO reverted the effect (**Figure 5K**), indicating a potential defect in killing following inhibition of protein synthesis. The percentage of live CD8^+^ T cells remained unchanged for all treatments (**Figure 5L**).

Altogether, these data suggest that cytosolic protein synthesis is required for a polyfunctional response early after T-cell activation of CTLs by preventing the adoption of cell asymmetry through defective cytoskeletal dynamics, although their potential ability to degranulate is preserved as observed through CD107a expression in PMA- and ionomycin-stimulated CTLs.

### Inhibition of cytosolic protein synthesis modulates the binding of tubulin molecular motors to mitochondria

**Figure 6:**
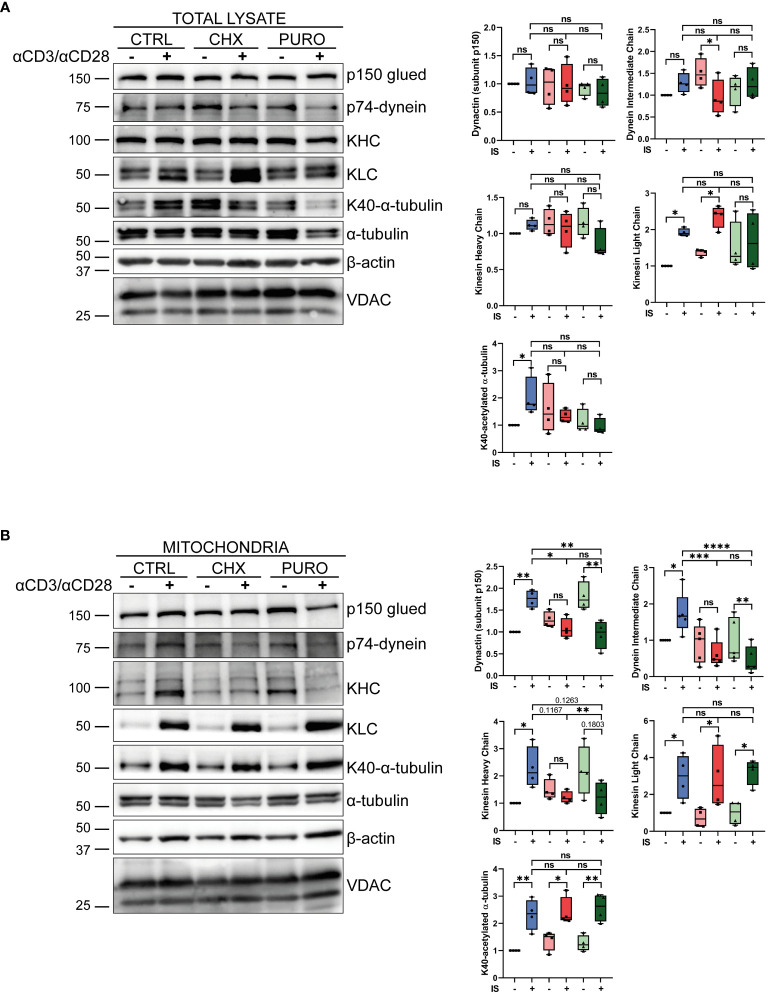
Inhibition of cytosolic protein synthesis during T-cell receptor (TCR) and CD28 stimulation modulates the recruitment of tubulin molecular motors to mitochondria in human CD8^+^ cytotoxic T lymphocytes. **(A, B)** Western blotting showing distribution of components of dynein (p74-dynein intermediate chain) and kinesin-1 [Kinesin heavy chain (KHC); kinesin light chain (KLC)] molecular motors and the dynactin adaptor (p150-Glued), K40-acetylated and total α-tubulin, and β-actin and VDAC in **(A)** total lysate and **(B)** isolated mitochondria from cytotoxic T lymphocytes (CTLs) treated with vehicle (CTRL), cycloheximide (CHX; 20 μg/mL), or puromycin (PURO; 50 μg/mL) for 1 h and stimulated with αCD3/αCD28 tetramers for 15 min. A representative experiment out of four is shown. Graphs showing densitometric quantification ratios of indicated proteins normalised to VDAC and referenced to non-stimulated CTRL. Box and whiskers, data are median plus min and max from n = 4 healthy donors; two way ANOVA. *, p < 0.05; **, p < 0.01; ***, p < 0.001; ****, p < 0.0001; ns, non-significant.

To investigate the potential mechanism by which inhibition of cytosolic protein synthesis affects the association between MTs and organelles such as mitochondria, the association of relevant molecular motors was studied in CTLs pre-treated with vehicle, CHX, or PURO early after TCR and CD28 activation (**Figure 6**). In total lysates (**Figure 6A**), the adaptor dynactin (p150 subunit), which helps kinesin and dynein to bind to different organelles (53), was unaffected in CHX- or PURO-treated CTLs relative to control or activated CTLs. Kinesin-1, a complex formed by kinesin heavy chain (KHC) and kinesin light chain (KLC) relevant for anterograde transport in CTLs (54), was also investigated. KHC was not affected by activation or inhibition of protein synthesis, whereas KLC increased in CTRL-treated CTLs after activation. This increase was also observed in CHX-treated CTLs, but not in PURO-treated CTLs. The dynein intermediate chain (p74) remained unchanged. As already observed (**Figure 1D**), CTLs treated with CHX or PURO showed decreased tubulin acetylation at K40 in contrast to CTRL-treated CTLs showing increased tubulin acetylation after TCR activation.

Isolated mitochondria (**Figure 6B**) from activated CTRL-treated CTLs showed increased levels of p150 subunit, p74, and KHC, whereas they remained unchanged in mitochondria from CHX but decreased in PURO. Similar increased levels of β-actin, KLC, and tubulin acetylation were observed in mitochondria after activation in control and treated CTLs. Taken together, these results suggest an impairment of the molecular motors involved in mitochondrial transport in CTLs after TCR stimulation and co-stimulation when cytosolic protein synthesis is inhibited.

## Discussion

This study investigated the altered reorganisation of the tubulin cytoskeleton and mitochondrial network and subsequently in the metabolic fate and effector function of human CTLs due to short-term inhibition of protein synthesis at eukaryotic ribosomes by puromycin and cycloheximide treatment after TCR activation and co-stimulation. Although both compounds prevent the synthesis of proteins during the elongation of the nascent polypeptides, they show different mechanisms of action. Puromycin couples covalently to the carboxyl-activated polypeptide at the ribosomal peptidyl-tRNA binding site (P site) adjacent to the ribosomal aminoacyl-tRNA binding site (A site). A site is used by puromycin to enter the ribosome and bind, causing rapid chain termination by covalently attaching to the C-terminus of the nascent chain, producing a C-terminal puromycylated polypeptide (46); puromycin is also known to act in the 80S and 55S ribosomes (47). However, cycloheximide binds to the E-site, which is located next to the deacylated tRNA of the 60S ribosome, and inhibits eEF2-mediated translocation, causing the ribosome to arrest on the second codon (45). These mechanisms of action could explain the differences in the observed effects in this study for mitochondrial localisation and activity.

Here, we studied the effect of short-term inhibition of protein translation on the polyfunctionality of CTLs; puromycin showed greater activity than CHX at the doses tested, as well as a potential ability to enter the mitochondria. In this context, the phosphorylation of proteins involved in early TCR signalling, such as PLCγ1, and downstream signalling, such as Erk1/2, was decreased by inhibition of cytosolic protein synthesis. mTOR signalling is also defective in our study after short-term inhibition of protein synthesis, with defective Akt, mTOR, and S6 phosphorylation early upon TCR stimulation and CD28 co-stimulation. The contribution of mTOR signalling to pathways leading to full CD8^+^ T-cell activation has been demonstrated in several immune contexts, including infection (55–57). mTOR is a key molecule for fine-tuning metabolic pathways in T cells and regulating their differentiation and exhaustion status (34–37). In this regard, cytosolic protein synthesis plays a regulatory role in the TCR-dependent cell growth required for full T-cell effector functions; mTOR activity is required in activation processes leading to *de novo* synthesis of >2,300 proteins in CD8^+^ T cells (33). Human T cells accumulate mRNAs ready for use in ribosomes upon cell stimulation to enable a rapid response to TCR activation through translation (27, 58).

Inhibition of protein translation on 80S ribosomes in CTLs by short-term treatment prevented the asymmetric localisation of the centrosome in human CTLs, with reduced stabilisation of MTs in cells as observed by a lower ratio of MT acetylation after TCR and CD28 activation at 30 min after activation. The changes in the kinetics of tubulin acetylation observed in CTLs treated with CHX or PURO suggest a decrease in MT dynamics upon CTL activation. This is consistent with previous data on human CD4 T cells examining *de novo* cytosolic protein translation and subsequent folding during short-term T-cell activation (24). MT dynamics is a highly regulated process dependent on protein translation and folding (59) that facilitates centrosome localisation (24, 52, 60, 61) in coordination with actin dynamics (16) during CD4 T-cell activation. This is consistent with the results shown in this work during the activation of primary human CTLs. Moreover, both the growth of MTs, regulated by proteins such as EB1, Plk1, and Aurora Kinase A (52, 62, 63), and their stability status, regulated by HDAC6 through the balance of the post-translational modification (PTM) of α-tubulin by acetylation/deacetylation at K40 (64), are induced by TCR activation. K40 tubulin acetylation is a hallmark of MT stability (50) and affects protein interactions with MTs, including kinesin binding; the frequency of bundling increases the number of kinesin binding sites (65, 66) and regulates the transport of vesicles and organelles in cells (67). Kinesin-1 facilitates anterograde movement (68), and dynein regulates retrograde movement by transporting material towards the minus ends of the MTs, moving from the cell periphery to the cell interior (69). Both motors can bind the adaptor dynactin (p150^Glued^) to anchor cargoes (53). TCR signalling promotes the synaptic recruitment of the dynein–dynactin complex (15, 70), which can provide traction forces on MTs radiating from the centrosome (71) and regulate mitochondrial transport (72). Here, dynein (p74-dynein intermediate chain) and kinesin (mainly KHC and KLC) increased their binding to mitochondria upon TCR activation. CHX treatment prevented the increase in p150-dynactin, p74-dynein, and KHC binding, and PURO treatment promoted a dramatic decrease in p150-dynactin and p74-dynein interaction with mitochondria in activated CTLs. The differences in the recruitment of molecular motors to mitochondria can explain their differential localisation regarding the centrosome, with PURO mitochondria being close to the centrosome due to a lack of dynein activity. However, the increase in acetylation of the contacting tubulin remained similar, as well as the recruitment of KLC in these mitochondria, which deserves further research and may suggest that other kinesin motors apart from kinesin-1 can be involved in mitochondrial movement. Additionally, cells lost their uropod, which points to preserved cell plasticity in response to TCR activation.

Mitochondria are critical for optimal T-cell function; these organelles provide ATP and metabolic mediators and act as regulators of intracellular calcium flux for CTL migration, development, and TCR signalling (20, 22, 31, 38). Regulators of the tubulin cytoskeleton, such as EB1 or CCT, play a key role in the reorganisation of the mitochondrial network and the metabolic capacity of T cells after activation (24, 25). In this sense, the altered polarisation of the centrosome towards the IS in CTLs treated with CHX or PURO may partly account for the defect in mitochondrial mobilisation; the defect in the recruitment of molecular motors may also explain the localisation of mitochondria and their defective distribution in the active site. The defective localisation of the mitochondria may be relevant to their function and the metabolic fate of T cells. We observed that these *in vitro* differentiated CTLs showed only a weak, non-significant response in the mitostress test to activation with anti-CD3 plus anti-CD28 antibodies (ImmunoCult T cell activator; 1 h) prior to measurement of OCR and ECAR, which tend to increase. However, for both compounds, CHX and PURO, all the parameters observed were decreased and showed no difference between resting and stimulated CTLs. As these inhibitors were added to the cell media shortly before activation, these data support an active role for cytosolic protein translation during CTL activation to promote proper mitochondrial respiration and an increase after TCR activation and CD28 co-stimulation.

Lytic granule exocytosis is a highly regulated process dependent on the intensity and kinetics of TCR signalling (73–75). The observed defect in early TCR signalling in CHX- and PURO-treated CTLs, with decreased phosphorylation kinetics of molecules such as PLCγ1 and Erk1/2, could explain the altered polarisation of lytic granules towards the synapse site. Taken together, these factors affect the effector function of CTLs, resulting in impaired degranulation, killing capacity, and pro-inflammatory cytokine production through short-term inhibition of cytosolic protein synthesis.

Mouse *Usp30*-deficient CTLs show reduced levels of TNF-α and also perforin and granzyme in lytic granules due to defective mitochondrial translation, which promotes chronic reduction in cytosolic ribosomal activity, but no defects in the ability of CTLs to adopt an asymmetric shape and to interact with the target (39). Here, we demonstrate the relevant role of cytosolic protein translation in facilitating human CTL functionality that is probably potentiated by mitochondrial ribosomes, as deduced by the potential effect of puromycin on 55S ribosomes. This includes the adoption of cell asymmetry for CTL effector function. We observed a decrease in IFN-γ in active CTLs for 30 min upon CHX and PURO treatment, although its release was not dramatically inhibited by puromycin at short times, and the differences in the percentage of CTLs containing IFN-γ were not significant upon 5 h, even upon complete inhibition of translation. However, IFN-γ secretion and killing of CD14^+^ cells by stimulation with specific CMV peptides of human PBMCs were prevented by both treatments at 18 h; puromycin was more effective in inhibiting these actions. These results suggest that the production of IFN-γ during CTL differentiation is enough for the correct biogenesis of vesicles that are ready to be released without a requirement for directed secretion of IFN-γ at the contact site with the target cell. Tubulin-based molecular motors assist in delivering lytic granules to the active killing site of CTLs. Dynein controls the targeting of lytic granules to the centrosome (76), while the kinesin-1-Slp3-Rab27a complex directs the terminal transport to the plasma membrane for exocytosis (54). Dynactin may also be a component of this complex, binding cargo to the kinesin-1 motor (77, 78). This process is also regulated by HDAC6, which helps to form the p150 and kinesin complex used for cargo in mouse CTLs (43). In this context, cytosolic protein translation may affect the movement of lytic granules to the active site via kinesin, as well as to the centrosome via dynein, since the components of these molecular motors showed defective recruitment to the mitochondria, which may be paralleled by lytic granules. In this sense, our experiments showing a defective recruitment of lytic granules to the active site by TIRFm support a defective transport of lytic granules from the centrosome to the stimulatory surface mimicking the target cell. These data, together with the altered polarisation of the centrosome, point to a failure of transport along MTs as a cause of defective degranulation of human CTLs treated with CHX or PURO.

Therefore, we described here that TCR and CD28 activation promotes early induction of protein synthesis on cytosolic ribosomes, which is essential to allow full activation of CD8^+^ T cells and adoption of the cell asymmetry required for CTL effector function.

## Data availability statement

The original contributions presented in the study are included in the article/**Supplementary Material**. Further inquiries can be directed to the corresponding author.

## Ethics statement

The studies involving humans were approved by Comité para la Ética en la Investigación Médica (CEIm) under Protocol 4707 to NM-C. Hospital Universitario La Princesa. Diego de Leon 62, 28006. Madrid. Spain. The studies were conducted in accordance with the local legislation and institutional requirements. The human samples used in this study were acquired from a by- product of routine care or industry. Written informed consent for participation was not required from the participants or the participants’ legal guardians/next of kin in accordance with the national legislation and institutional requirements.

## Author contributions

ÁG-M: Conceptualization, Data curation, Writing – original draft, Writing – review & editing, Formal analysis, Investigation, Methodology, Software. IT: Conceptualization, Data curation, Investigation, Methodology, Software, Writing – review & editing. CS: Investigation, Methodology, Writing – review & editing. CP: Investigation, Methodology, Writing – review & editing. ML-P: Writing – review & editing, Data curation, Formal analysis, Software. PM-F: Writing – review & editing, Methodology, Data curation, Formal analysis, Investigation. SR: Writing – review & editing, Methodology. PM: Funding acquisition, Resources, Visualization, Writing – review & editing. EM-G: Funding acquisition, Resources, Supervision, Visualization, Writing – review & editing, Investigation, Methodology. AA: Formal analysis, Funding acquisition, Resources, Supervision, Writing – review & editing. NM-C: Conceptualization, Data curation, Funding acquisition, Project administration, Resources, Supervision, Visualization, Writing – original draft, Writing – review & editing.
