# Corrigendum: Cytosolic protein translation regulates cell asymmetry and function in early TCR activation of human CD8^+^ T lymphocytes

**DOI:** 10.3389/fimmu.2024.1500666

**Published:** 2024-10-14

**Authors:** Álvaro Gómez-Morón, Ilya Tsukalov, Camila Scagnetti, Clara Pertusa, Marta Lozano-Prieto, Pedro Martínez-Fleta, Silvia Requena, Pilar Martín, Aranzazu Alfranca, Enrique Martin-Gayo, Noa B Martin-Cofreces

**Affiliations:** ^1^ Immunology Service, Instituto de Investigación Sanitaria del Hospital Universitario La Princesa, IIS- Princesa, Universidad Autónoma de Madrid (UAM), Madrid, Spain; ^2^ Department of Immunology, Ophthalmology and ENT, School of Medicine, Universidad Complutense de Madrid and 12 de Octubre Health Research Institute (imas12), Madrid, Spain; ^3^ Medicine Faculty, Universidad Autónoma de Madrid (UAM), Madrid, Spain; ^4^ Videomicroscopy Unit, Instituto de Investigación Sanitaria del Hospital Universitario La Princesa, IIS-Princesa, Universidad Autónoma de Madrid (UAM), Madrid, Spain; ^5^ Centro de Investigación Biomédica en Red de Enfermedades Cardiovasculares (CIBERCV), Instituto de Salud Carlos III, Madrid, Spain; ^6^ Area of Vascular Pathophysiology, Laboratory of Regulatory Molecules of Inflammatory Processes, Fundación Centro Nacional de Investigaciones Cardiovasculares-Carlos III, Madrid, Spain; ^7^ Centro de Investigación Biomédica en Red Enfermedades Infecciosas (CIBERINFECC), Instituto de Salud Carlos III, Madrid, Spain; ^8^ Area of Vascular Pathophysiology, Laboratory of Intercellular Communication, Fundación Centro Nacional de Investigaciones Cardiovasculares-Carlos III, Madrid, Spain

**Keywords:** protein translation, cell asymmetry, cytotoxic CD8^+^ T lymphocytes, cytoskeleton, metabolism, mitochondria, T cell activation, immunological synapse

In the published article, there was an error. The name “70S” was used to name mitochondrial ribosomes instead of “55S”.

A correction has been made to **Abstract**, *Introduction*. This sentence previously stated:

“Mitochondria play a role in regulating CTL function by controlling processes such as calcium flux, providing the necessary energy through oxidative phosphorylation, and its own protein translation on 70S ribosomes.”

The corrected sentence appears below:

“Mitochondria play a role in regulating CTL function by controlling processes such as calcium flux, providing the necessary energy through oxidative phosphorylation, and its own protein translation on 55S ribosomes.”

A correction has also been made to **Abstract**, *Methods*. This sentence previously stated:

“Here, we investigated the importance of cytosolic protein synthesis in human CTLs after early TCR activation and CD28 co-stimulation for the dynamic reorganisation of the cytoskeleton, mitochondria, and lytic granules through short-term chemical inhibition of 80S ribosomes by cycloheximide and 80S and 70S by puromycin.”

The corrected sentence appears below:

“Here, we investigated the importance of cytosolic protein synthesis in human CTLs after early TCR activation and CD28 co-stimulation for the dynamic reorganisation of the cytoskeleton, mitochondria, and lytic granules through short-term chemical inhibition of 80S ribosomes by cycloheximide and 80S and 55S by puromycin.”

A correction has been made to **Introduction**, Paragraph 3. This sentence previously stated:

“CTLs require correct translation at mitochondrial 70S ribosomes to help CD8+ cytotoxic T cell-mediated prolonged killing (39).”

The corrected sentence appears below:

“CTLs require correct translation at mitochondrial 55S ribosomes to help CD8+ cytotoxic T cell-mediated prolonged killing (39).”

A correction has been made to **Results**, Eukaryotic protein translation inhibition affects tubulin cytoskeleton dynamics and TCR signalling of human cytotoxic T lymphocytes, Paragraph 1. This sentence previously stated:

“Indeed, PURO has also been used to inhibit mitochondrial 70S ribosomes *in vitro* (47).”

The corrected sentence appears below:

“Indeed, PURO has also been used to inhibit mitochondrial 55S ribosomes *in vitro* (47).”

A correction has also been made to **Results**, Eukaryotic protein translation inhibition affects tubulin cytoskeleton dynamics and TCR signalling of human cytotoxic T lymphocytes, Paragraph 3. This sentence previously stated:

“Indeed, inhibition of 70S mitochondrial ribosomes could also increase the effect in CTLs after puromycin treatment.”

The corrected sentence appears below:

“Indeed, inhibition of 55S mitochondrial ribosomes could also increase the effect in CTLs after puromycin treatment.”

A correction has been made to **Discussion**, Paragraph 1. This sentence previously stated:

“A site is used by puromycin to enter the ribosome and bind, causing rapid chain termination by covalently attaching to the C-terminus of the nascent chain, producing a Cterminal puromycylated polypeptide (46); puromycin is also known to act in the 80S and 70S ribosomes (47).”

The corrected sentence appears below:

“A site is used by puromycin to enter the ribosome and bind, causing rapid chain termination by covalently attaching to the C-terminus of the nascent chain, producing a Cterminal puromycylated polypeptide (46); puromycin is also known to act in the 80S and 55S ribosomes (47).”

A correction has also been made to **Discussion**, Paragraph 6. This sentence previously stated:

“Here, we demonstrate the relevant role of cytosolic protein translation in facilitating human CTL functionality that is probably potentiated by mitochondrial ribosomes, as deduced by the potential effect of puromycin on 70S ribosomes.”

The corrected sentence appears below:

“Here, we demonstrate the relevant role of cytosolic protein translation in facilitating human CTL functionality that is probably potentiated by mitochondrial ribosomes, as deduced by the potential effect of puromycin on 55S ribosomes.”

The authors apologize for these errors and state that they do not change the scientific conclusions of the article in any way. The original article has been updated.

