# Supplementary material for: Cytosolic protein translation regulates cell asymmetry and function in early TCR activation of human CD8+ T lymphocytes
Source: Front Immunol. 2024 Jul 24;15:1411957. doi: 10.3389/fimmu.2024.1411957 (PMC11303187; doi:10.3389/fimmu.2024.1411957)
Supplement: Supplementary file 1 [file DataSheet_1.pdf]

Supplementary Material

Cytosolic protein translation regulates cell asymmetry and function in early TCR-activation of human CD8$^+$ T lymphocytes.

Álvaro Gómez-Morón , Ilya Tsukalov , Camila Scagnetti, Clara Pertusa, Marta Lozano-Prieto, Pedro Martínez-Fleta, Silvia Requena, Pilar Martin, Arantzazu Alfranca and Enrique Martin-Gayo and Noa Beatriz Martín-Cófreces

Correspondence: Corresponding Author: noa.martin@salud.madrid.org

1 Supplementary Data

Supplementary file | Linear mixed model analysis for Seahorse experiments. Corresponds to Figures 3 and 4.

2 Supplementary Figures and Tables

2.1 Supplementary Figures

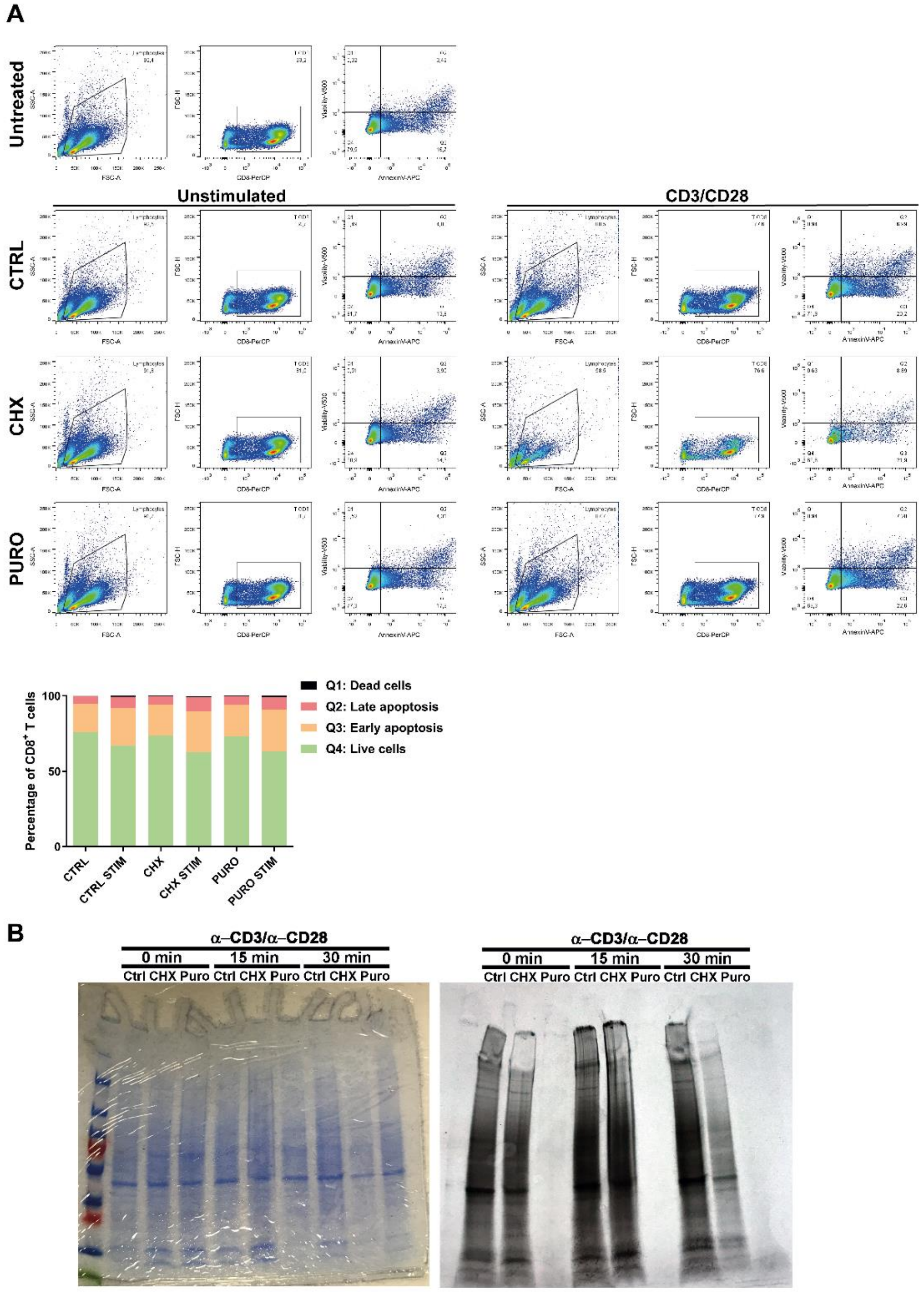

Supplementary Figure 1. (A) Cell viability of CTLs treated with vehicle (CTRL), cycloheximide (CHX, 20 μg/mL) or puromycin (PURO, 50 μg/mL) for 1h and stimulated with anti-CD3/CD28 antibodies for 30 min. Cells were stained with AnnexinV-APC and Ghost dye Violet viability dye. n = 4. **(B)** Protein translation monitored by ^{35}S-Cys/Met metabolic labelling of CTLs treated with vehicle (CTRL), cycloheximide (CHX, 20 μg/mL) or puromycin (PURO, 50 μg/mL) for 1h, unstimulated or stimulated with anti-CD3/CD28 at indicated times. Left, Coomassie staining; right, autoradiographic exposure for 72 h. Experiments were performed with 3 different healthy donors.

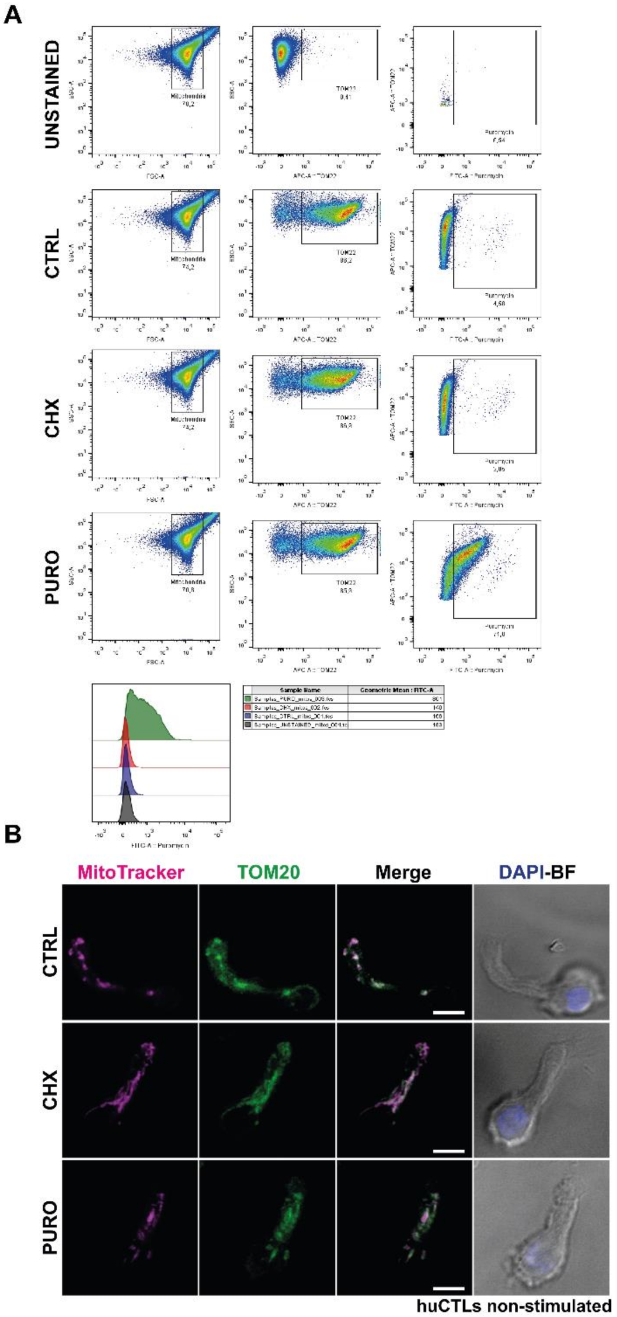

Supplementary Figure 2. (A) Isolated mitochondria from CTLs treated with vehicle (CTRL), cycloheximide (CHX, 20 µg/mL) or puromycin (PURO, 50 µg/mL) for 1h and isolated mitochondria were stained with Alexa 488 anti-puromycin antibody. Gating strategy and Puromycin GeoMean of one experiment is shown**. (B)** Representative fluorescent images from confocal microscopy of human cytotoxic CD8$^+$ T lymphocytes pre-treated with vehicle (CTRL), cycloheximide (CHX, 20 µg/mL) or puromycin (PURO, 50 µg/mL) for 1h and spreading over poly-L-Lys-coated coverslips. Magenta, MitoTracker Orange; Green, Tom-20; Blue, DAPI; BF, brightfield. Images are single focus planes of the IS plane. Bar, 5 µm**. Corresponds to Figure 2.**

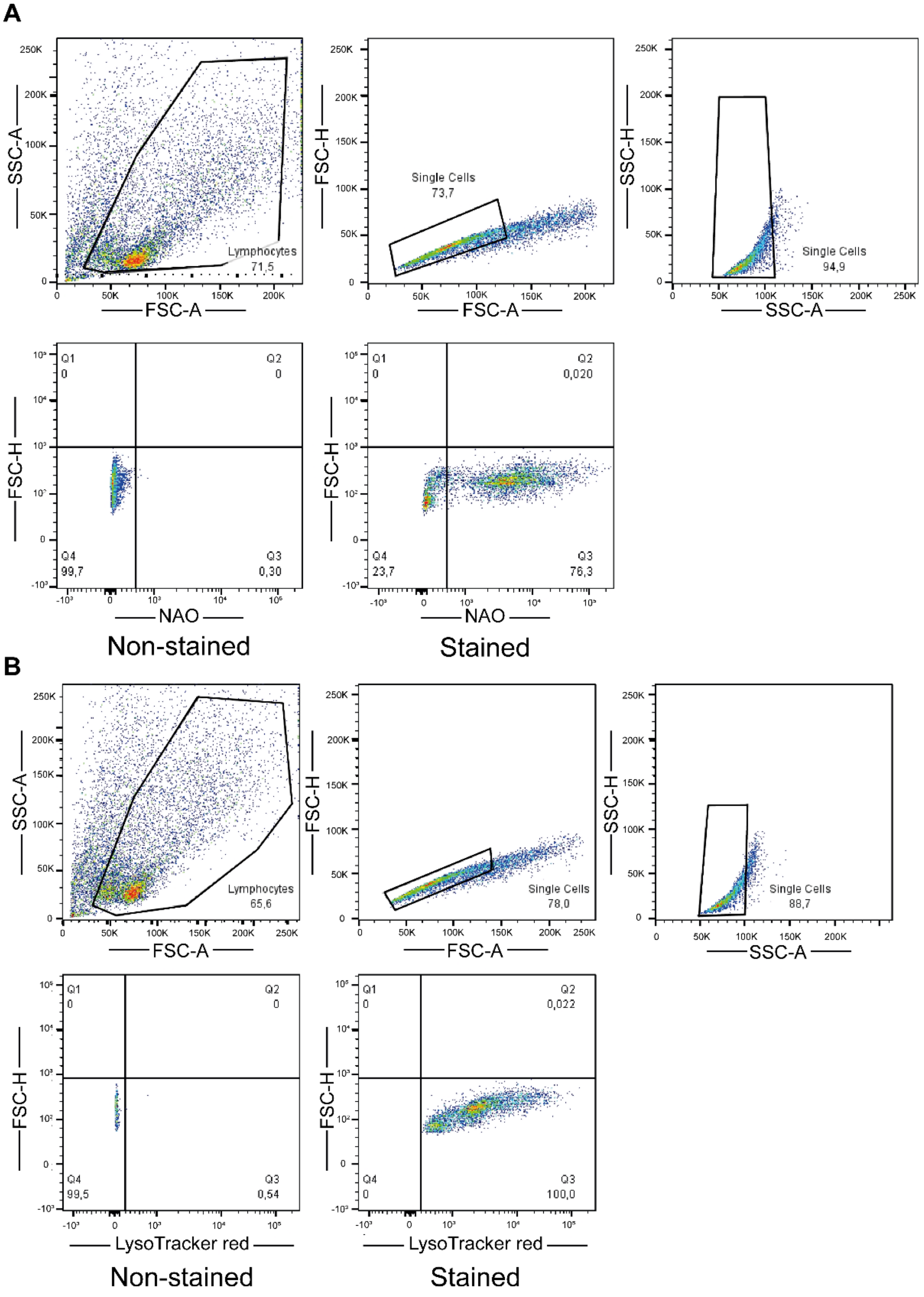

Supplementary Figure 3. Gating strategy used to measure **(A)** mitochondrial mass with Nonyl-acridine orange (NAO) and **(B)** lytic granule fluorescence with LysoTracker-Red DND-99 by flow cytometry. **Corresponds to Figure 3E and 5B.**

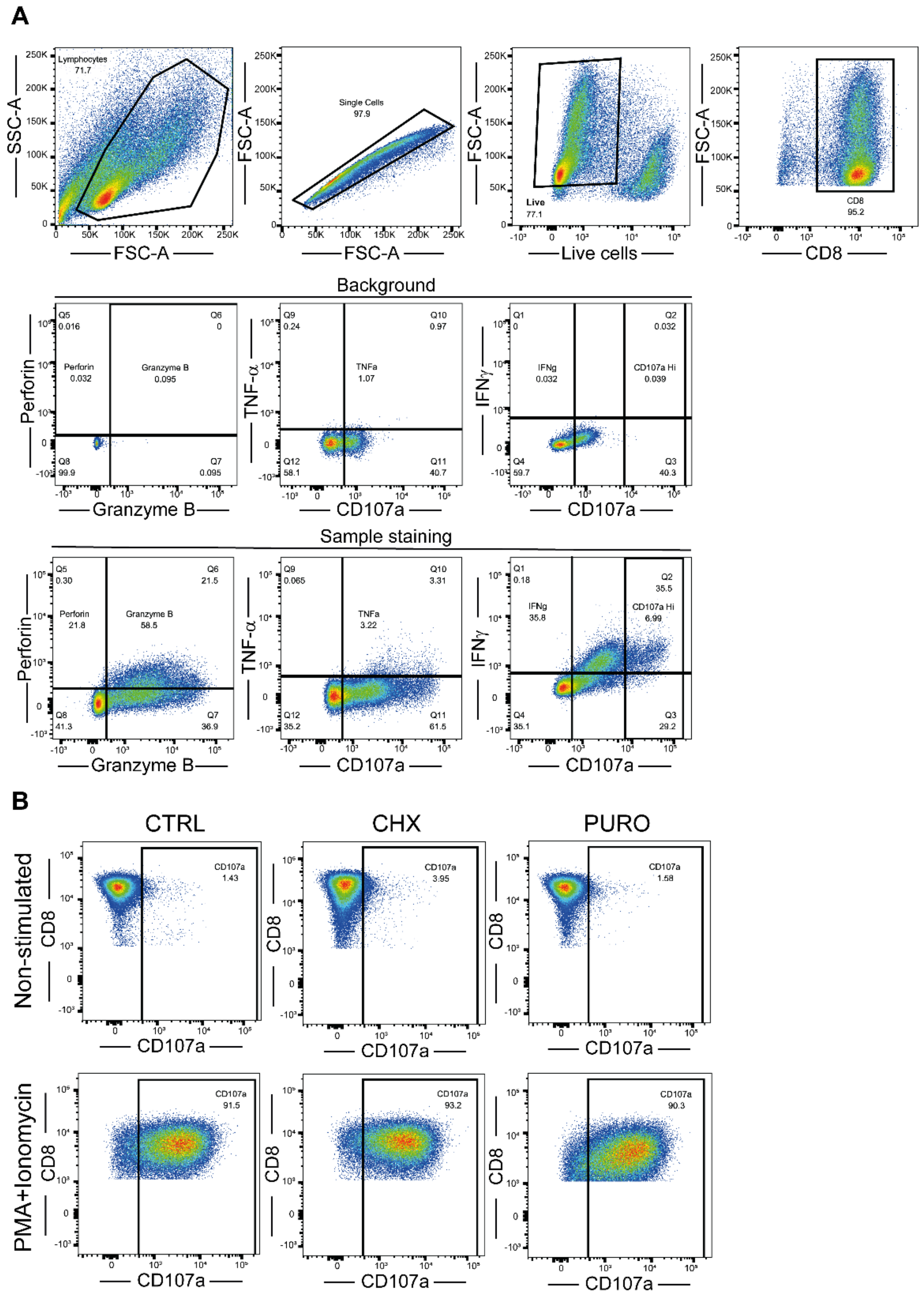

Supplementary Figure 4. (A) Gating strategy used to analyse human cytotoxic T lymphocytes effector function and degranulation capacity by flow cytometry. **(B)** Effect of short-term cytosolic protein translation inhibition in the degranulation capacity (CD107a expression in membrane) of non-stimulated and PMA/ionomycin-stimulated human cytotoxic T lymphocytes through flow cytometry. **Corresponds to Figure 5.**

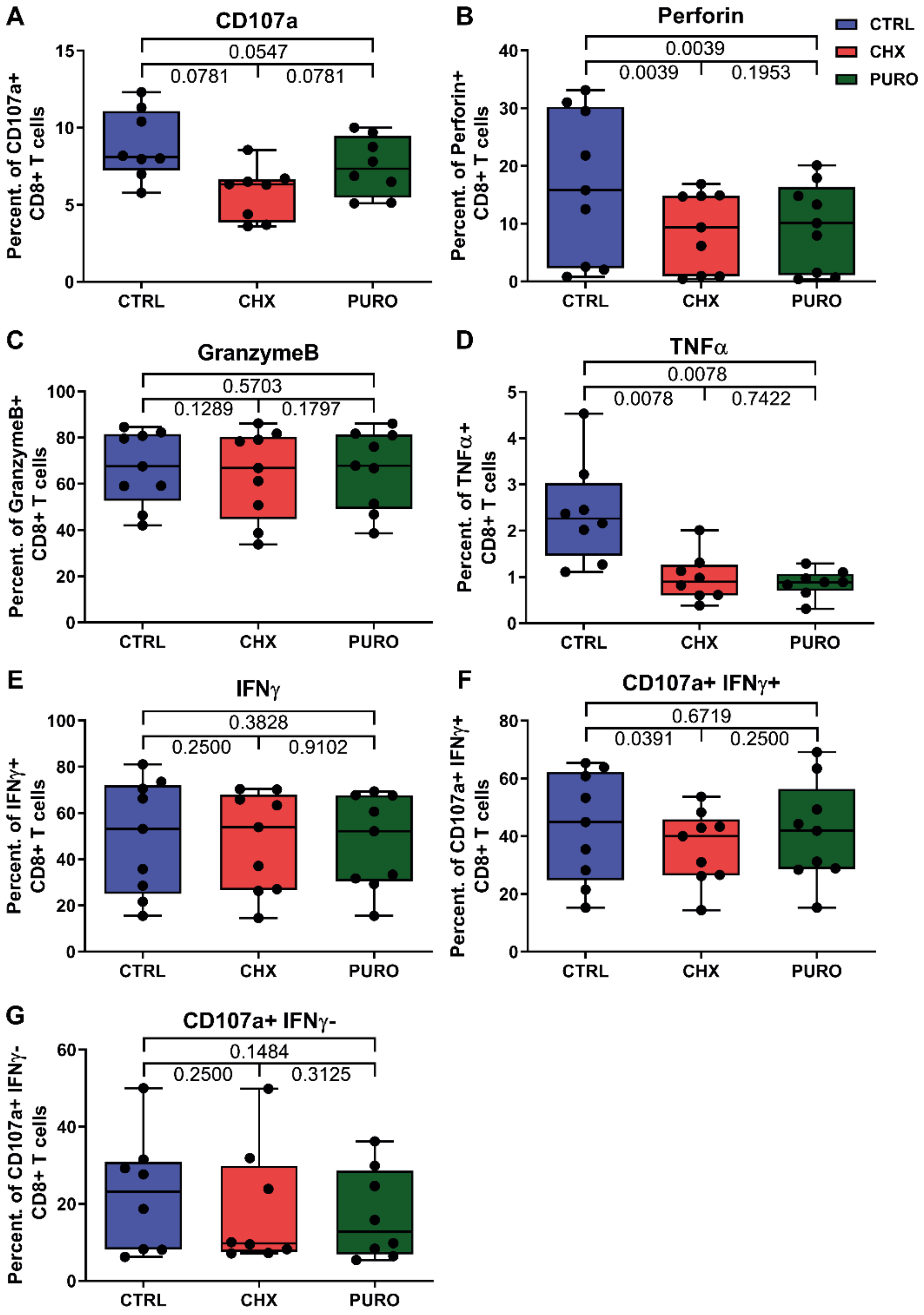

Supplementary Figure 5. Raw data from proportions of CD8^{+} T cells expressing **(A)** CD107a, **(B)** Perforin, **(C)** Granzyme B, **(D)** TNF-α, **(E)** IFN-γ **(F)** CD107a^{+}, IFN-γ^{+} and **(G)** CD107a^{+} IFN-γ^{-}, to study cytotoxic T lymphocyte effector function and degranulation capacity by flow cytometry. **Corresponds to Figure 5.**

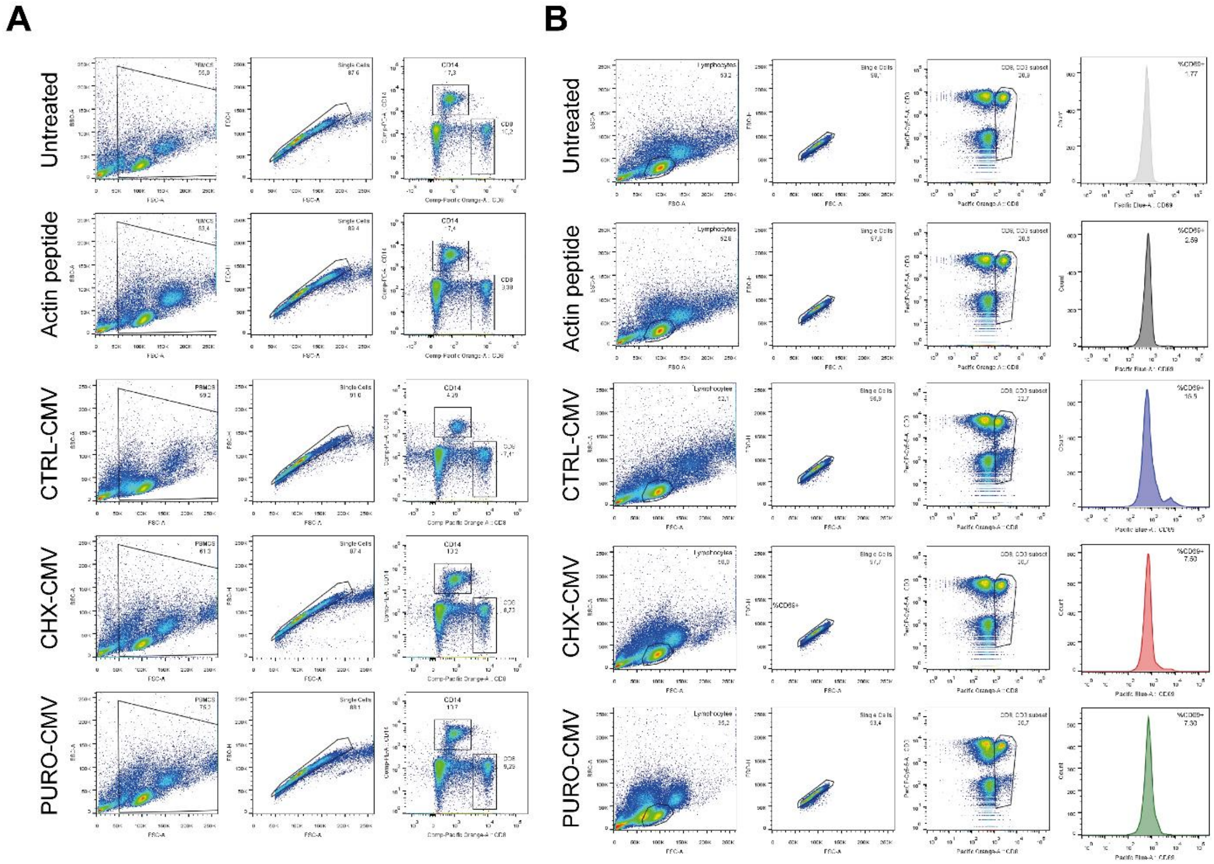

Supplementary Figure 6. (A) Gating strategy for killing assay of CD14^{+} cells (monocytes) by CD8^{+} activated with CMV-specific peptides for 18 h. A representative experiment from 3 is shown. Actin peptide was used as negative control. **(B)** Gating strategy for CD69 expression as a readout for CMV-specific activation of CD8+ cells. **Corresponds to Figure 5.**
