# Supplementary material for: Cytosolic protein translation regulates cell asymmetry and function in early TCR activation of human CD8+ T lymphocytes
Source: Front Immunol. 2024 Jul 24;15:1411957. doi: 10.3389/fimmu.2024.1411957 (PMC11303187; doi:10.3389/fimmu.2024.1411957)
Supplement: Supplementary file 2 [file DataSheet_2.pdf]

# Seahorse statistical analysis

```
library(lattice)
library(AICcmodavg)
library(emmeans)
library(lme4)
```

```
## Loading required package: Matrix

##
## Attaching package: 'lme4'

## The following object is masked from 'package:AICcmodavg':
##
##     checkConv
```

```
library(lmerTest)
```

```
##
## Attaching package: 'lmerTest'

## The following object is masked from 'package:lme4':
##
##     lmer

## The following object is masked from 'package:stats':
##
##     step
```

```
library(lmtest)
```

```
## Loading required package: zoo

##
## Attaching package: 'zoo'

## The following objects are masked from 'package:base':
##
##     as.Date, as.Date.numeric
```

```
library(ggplot2)
```

## ECAR - Glycostress

```r
seahorse5$time <- as.numeric(seahorse5$time)
ggplot(seahorse5, aes(time, value, colour=condition))+
  geom_point()+
  theme_bw()+
  xlab("Time (min)") +
  ylab('Value')+
  facet_wrap(~ind)
```

```
## Warning: Removed 6 rows containing missing values (`geom_point()`).
```

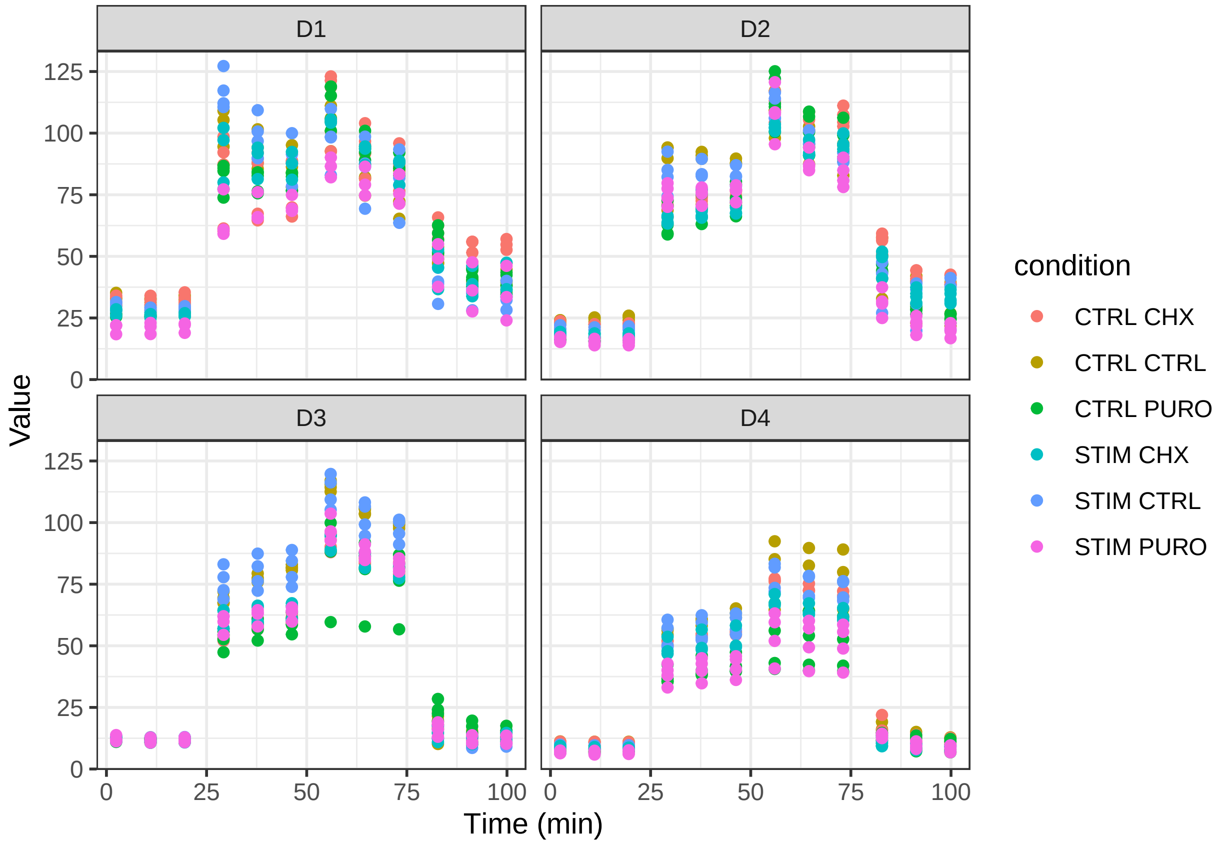


```r
m5<-lmer(value ~ treatment*condition + (1|ind), data=seahorse5)
summary(m5)
```

```
## Linear mixed model fit by REML. t-tests use Satterthwaite's method [
## lmerModLmerTest]
## Formula: value ~ treatment * condition + (1 | ind)
##    Data: seahorse5
##
## REML criterion at convergence: 8151.7
##
## Scaled residuals:
##     Min      1Q  Median      3Q     Max
## -4.2570 -0.5702 -0.0644  0.6275  3.7037
```

```
##
## Random effects:
##  Groups   Name        Variance Std.Dev.
##  ind      (Intercept) 162.8    12.759
##  Residual              88.7     9.418
## Number of obs: 1122, groups:  ind, 4
##
## Fixed effects:
##                                 Estimate Std. Error         df t value Pr(>|t|)
## (Intercept)                     18.76298    6.52309    3.26547   2.876  0.05757
## treatmentb                      47.17815    1.92247 1094.99936  24.540  < 2e-16
## treatmentc                      71.87382    1.92247 1094.99936  37.386  < 2e-16
## treatmentd                      13.65951    1.92247 1094.99936   7.105 2.16e-12
## conditionCTRL CTRL               0.41417    1.95447 1095.00032   0.212  0.83222
## conditionCTRL PURO              -3.70821    1.86864 1095.00096  -1.984  0.04746
## conditionSTIM CHX               -2.86336    1.99095 1095.00362  -1.438  0.15067
## conditionSTIM CTRL              -1.14466    1.92315 1095.00259  -0.595  0.55183
## conditionSTIM PURO              -4.66506    1.95587 1095.00672  -2.385  0.01724
## treatmentb:conditionCTRL CTRL   12.25639    2.76372 1094.99936   4.435 1.01e-05
## treatmentc:conditionCTRL CTRL    1.11979    2.76372 1094.99936   0.405  0.68543
## treatmentd:conditionCTRL CTRL   -5.78059    2.76372 1094.99936  -2.092  0.03670
## treatmentb:conditionCTRL PURO   -0.05975    2.64218 1094.99936  -0.023  0.98196
## treatmentc:conditionCTRL PURO   -3.25845    2.64218 1094.99936  -1.233  0.21775
## treatmentd:conditionCTRL PURO   -1.71045    2.64218 1094.99936  -0.647  0.51753
## treatmentb:conditionSTIM CHX     3.84690    2.81420 1094.99936   1.367  0.17192
## treatmentc:conditionSTIM CHX    -1.77840    2.81420 1094.99936  -0.632  0.52756
## treatmentd:conditionSTIM CHX    -4.39139    2.81420 1094.99936  -1.560  0.11894
## treatmentb:conditionSTIM CTRL   15.31395    2.71878 1094.99936   5.633 2.25e-08
## treatmentc:conditionSTIM CTRL    1.97280    2.71878 1094.99936   0.726  0.46823
## treatmentd:conditionSTIM CTRL   -8.61414    2.76786 1095.00261  -3.112  0.00191
## treatmentb:conditionSTIM PURO    0.57276    2.76372 1094.99936   0.207  0.83586
## treatmentc:conditionSTIM PURO   -6.45369    2.76372 1094.99936  -2.335  0.01972
## treatmentd:conditionSTIM PURO   -6.21096    2.76372 1094.99936  -2.247  0.02482
##
## (Intercept)                   .
## treatmentb                    ***
## treatmentc                    ***
## treatmentd                    ***
## conditionCTRL CTRL
## conditionCTRL PURO            *
## conditionSTIM CHX
## conditionSTIM CTRL
## conditionSTIM PURO            *
## treatmentb:conditionCTRL CTRL ***
## treatmentc:conditionCTRL CTRL
## treatmentd:conditionCTRL CTRL *
## treatmentb:conditionCTRL PURO
## treatmentc:conditionCTRL PURO
## treatmentd:conditionCTRL PURO
## treatmentb:conditionSTIM CHX
## treatmentc:conditionSTIM CHX
## treatmentd:conditionSTIM CHX
## treatmentb:conditionSTIM CTRL ***
## treatmentc:conditionSTIM CTRL
```

```
## treatmentd:conditionSTIM CTRL **
## treatmentb:conditionSTIM PURO
## treatmentc:conditionSTIM PURO *
## treatmentd:conditionSTIM PURO *
## ---
## Signif. codes:  0 '***' 0.001 '**' 0.01 '*' 0.05 '.' 0.1 ' ' 1

##
## Correlation matrix not shown by default, as p = 24 > 12.
## Use print(x, correlation=TRUE)  or
##     vcov(x)        if you need it
```

```
summary(emmeans(m5, pairwise ~ condition|treatment, infer=T))
```

```
## $emmeans
## treatment = a:
##  condition emmean   SE   df lower.CL upper.CL t.ratio p.value
##  CTRL CHX    18.8 6.52 3.27   -1.071     38.6   2.876  0.0576
##  CTRL CTRL   19.2 6.53 3.28   -0.628     39.0   2.936  0.0543
##  CTRL PURO   15.1 6.51 3.23   -4.830     34.9   2.314  0.0973
##  STIM CHX    15.9 6.54 3.31   -3.873     35.7   2.430  0.0854
##  STIM CTRL   17.6 6.52 3.27   -2.217     37.5   2.701  0.0672
##  STIM PURO   14.1 6.53 3.28   -5.708     33.9   2.158  0.1120
##
## treatment = b:
##  condition emmean   SE   df lower.CL upper.CL t.ratio p.value
##  CTRL CHX    65.9 6.52 3.27   46.107     85.8  10.109  0.0014
##  CTRL CTRL   78.6 6.53 3.28   58.806     98.4  12.034  0.0008
##  CTRL PURO   62.2 6.51 3.23   42.288     82.1   9.555  0.0018
##  STIM CHX    66.9 6.54 3.31   47.152     86.7  10.228  0.0013
##  STIM CTRL   80.1 6.52 3.27   60.275     99.9  12.281  0.0008
##  STIM PURO   61.8 6.53 3.28   42.043     81.7   9.468  0.0017
##
## treatment = c:
##  condition emmean   SE   df lower.CL upper.CL t.ratio p.value
##  CTRL CHX    90.6 6.52 3.27   70.802    110.5  13.895  0.0005
##  CTRL CTRL   92.2 6.53 3.28   72.365    112.0  14.110  0.0005
##  CTRL PURO   83.7 6.51 3.23   63.785    103.6  12.858  0.0007
##  STIM CHX    86.0 6.54 3.31   66.223    105.8  13.143  0.0006
##  STIM CTRL   91.5 6.52 3.27   71.630    111.3  14.022  0.0005
##  STIM PURO   79.5 6.53 3.28   59.713     99.3  12.173  0.0008
##
## treatment = d:
##  condition emmean   SE   df lower.CL upper.CL t.ratio p.value
##  CTRL CHX    32.4 6.52 3.27   12.588     52.3   4.970  0.0127
##  CTRL CTRL   27.1 6.53 3.28    7.251     46.9   4.142  0.0214
##  CTRL PURO   27.0 6.51 3.23    7.119     46.9   4.150  0.0219
##  STIM CHX    25.2 6.54 3.31    5.395     44.9   3.846  0.0261
##  STIM CTRL   22.7 6.54 3.31    2.892     42.4   3.464  0.0348
##  STIM PURO   21.5 6.53 3.28    1.741     41.4   3.298  0.0401
##
## Degrees-of-freedom method: kenward-roger
## Confidence level used: 0.95
```

```
##
## $contrasts
## treatment = a:
##  contrast              estimate   SE   df lower.CL upper.CL t.ratio p.value
##  CTRL CHX - CTRL CTRL   -0.4142 1.95 1095  -5.9937    5.165  -0.212  0.9999
##  CTRL CHX - CTRL PURO    3.7082 1.87 1095  -1.6263    9.043   1.984  0.3519
##  CTRL CHX - STIM CHX     2.8634 1.99 1095  -2.8203    8.547   1.438  0.7036
##  CTRL CHX - STIM CTRL    1.1447 1.92 1095  -4.3455    6.635   0.595  0.9914
##  CTRL CHX - STIM PURO    4.6651 1.96 1095  -0.9185   10.249   2.385  0.1623
##  CTRL CTRL - CTRL PURO   4.1224 1.90 1095  -1.3051    9.550   2.168  0.2535
##  CTRL CTRL - STIM CHX    3.2775 2.02 1095  -2.4918    9.047   1.622  0.5842
##  CTRL CTRL - STIM CTRL   1.5588 1.95 1095  -4.0209    7.139   0.798  0.9680
##  CTRL CTRL - STIM PURO   5.0792 1.99 1095  -0.5911   10.750   2.557  0.1089
##  CTRL PURO - STIM CHX   -0.8448 1.94 1095  -6.3783    4.689  -0.436  0.9980
##  CTRL PURO - STIM CTRL  -2.5636 1.87 1095  -7.8979    2.771  -1.372  0.7439
##  CTRL PURO - STIM PURO   0.9569 1.90 1095  -4.4725    6.386   0.503  0.9961
##  STIM CHX - STIM CTRL   -1.7187 1.99 1095  -7.4007    3.963  -0.864  0.9550
##  STIM CHX - STIM PURO    1.8017 2.02 1095  -3.9677    7.571   0.891  0.9486
##  STIM CTRL - STIM PURO   3.5204 1.95 1095  -2.0593    9.100   1.801  0.4654
##
## treatment = b:
##  contrast              estimate   SE   df lower.CL upper.CL t.ratio p.value
##  CTRL CHX - CTRL CTRL  -12.6706 1.95 1095 -18.2501   -7.091  -6.483  <.0001
##  CTRL CHX - CTRL PURO    3.7680 1.87 1095  -1.5666    9.102   2.016  0.3335
##  CTRL CHX - STIM CHX    -0.9835 1.99 1095  -6.6672    4.700  -0.494  0.9964
##  CTRL CHX - STIM CTRL  -14.1693 1.92 1095 -19.6594   -8.679  -7.368  <.0001
##  CTRL CHX - STIM PURO    4.0923 1.96 1095  -1.4913    9.676   2.092  0.2920
##  CTRL CTRL - CTRL PURO  16.4385 1.90 1095  11.0110   21.866   8.646  <.0001
##  CTRL CTRL - STIM CHX   11.6870 2.02 1095   5.9177   17.456   5.783  <.0001
##  CTRL CTRL - STIM CTRL  -1.4987 1.95 1095  -7.0785    4.081  -0.767  0.9730
##  CTRL CTRL - STIM PURO  16.7629 1.99 1095  11.0925   22.433   8.439  <.0001
##  CTRL PURO - STIM CHX   -4.7515 1.94 1095 -10.2849    0.782  -2.451  0.1398
##  CTRL PURO - STIM CTRL -17.9372 1.87 1095 -23.2716  -12.603  -9.599  <.0001
##  CTRL PURO - STIM PURO   0.3244 1.90 1095  -5.1050    5.754   0.171  1.0000
##  STIM CHX - STIM CTRL  -13.1858 1.99 1095 -18.8678   -7.504  -6.625  <.0001
##  STIM CHX - STIM PURO    5.0758 2.02 1095  -0.6936   10.845   2.512  0.1215
##  STIM CTRL - STIM PURO  18.2616 1.95 1095  12.6819   23.841   9.343  <.0001
##
## treatment = c:
##  contrast              estimate   SE   df lower.CL upper.CL t.ratio p.value
##  CTRL CHX - CTRL CTRL   -1.5340 1.95 1095  -7.1135    4.046  -0.785  0.9701
##  CTRL CHX - CTRL PURO    6.9667 1.87 1095   1.6321   12.301   3.728  0.0028
##  CTRL CHX - STIM CHX     4.6418 1.99 1095  -1.0419   10.325   2.331  0.1823
##  CTRL CHX - STIM CTRL   -0.8281 1.92 1095  -6.3183    4.662  -0.431  0.9981
##  CTRL CHX - STIM PURO   11.1187 1.96 1095   5.5352   16.702   5.685  <.0001
##  CTRL CTRL - CTRL PURO   8.5006 1.90 1095   3.0731   13.928   4.471  0.0001
##  CTRL CTRL - STIM CHX    6.1757 2.02 1095   0.4064   11.945   3.056  0.0278
##  CTRL CTRL - STIM CTRL   0.7058 1.95 1095  -4.8739    6.286   0.361  0.9992
##  CTRL CTRL - STIM PURO  12.6527 1.99 1095   6.9824   18.323   6.370  <.0001
##  CTRL PURO - STIM CHX   -2.3249 1.94 1095  -7.8583    3.209  -1.199  0.8372
##  CTRL PURO - STIM CTRL  -7.7948 1.87 1095 -13.1291   -2.460  -4.172  0.0005
##  CTRL PURO - STIM PURO   4.1521 1.90 1095  -1.2772    9.581   2.183  0.2464
##  STIM CHX - STIM CTRL   -5.4699 1.99 1095 -11.1519    0.212  -2.748  0.0669
##  STIM CHX - STIM PURO    6.4770 2.02 1095   0.7076   12.246   3.205  0.0174
```

```
##  STIM CTRL - STIM PURO  11.9469 1.95 1095   6.3672   17.527   6.112  <.0001
##
## treatment = d:
##  contrast              estimate   SE   df lower.CL upper.CL t.ratio p.value
##  CTRL CHX - CTRL CTRL    5.3664 1.95 1095  -0.2131   10.946   2.746  0.0674
##  CTRL CHX - CTRL PURO    5.4187 1.87 1095   0.0841   10.753   2.900  0.0440
##  CTRL CHX - STIM CHX     7.2548 1.99 1095   1.5710   12.938   3.644  0.0038
##  CTRL CHX - STIM CTRL    9.7588 1.99 1095   4.0767   15.441   4.903  <.0001
##  CTRL CHX - STIM PURO   10.8760 1.96 1095   5.2924   16.460   5.561  <.0001
##  CTRL CTRL - CTRL PURO   0.0522 1.90 1095  -5.3753    5.480   0.027  1.0000
##  CTRL CTRL - STIM CHX    1.8883 2.02 1095  -3.8810    7.658   0.934  0.9376
##  CTRL CTRL - STIM CTRL   4.3924 2.02 1095  -1.3772   10.162   2.173  0.2511
##  CTRL CTRL - STIM PURO   5.5096 1.99 1095  -0.1607   11.180   2.774  0.0625
##  CTRL PURO - STIM CHX    1.8361 1.94 1095  -3.6974    7.370   0.947  0.9340
##  CTRL PURO - STIM CTRL   4.3401 1.94 1095  -1.1941    9.874   2.239  0.2208
##  CTRL PURO - STIM PURO   5.4574 1.90 1095   0.0281   10.887   2.870  0.0480
##  STIM CHX - STIM CTRL    2.5040 2.06 1095  -3.3655    8.374   1.218  0.8282
##  STIM CHX - STIM PURO    3.6213 2.02 1095  -2.1482    9.391   1.792  0.4714
##  STIM CTRL - STIM PURO   1.1172 2.02 1095  -4.6570    6.891   0.552  0.9939
##
## Degrees-of-freedom method: kenward-roger
## Confidence level used: 0.95
## Conf-level adjustment: tukey method for comparing a family of 6 estimates
## P value adjustment: tukey method for comparing a family of 6 estimates
```

```r
emmip(m5, condition ~ treatment) +
  theme_bw()+
  xlab('Treatment') +
  ylab('Value')
```

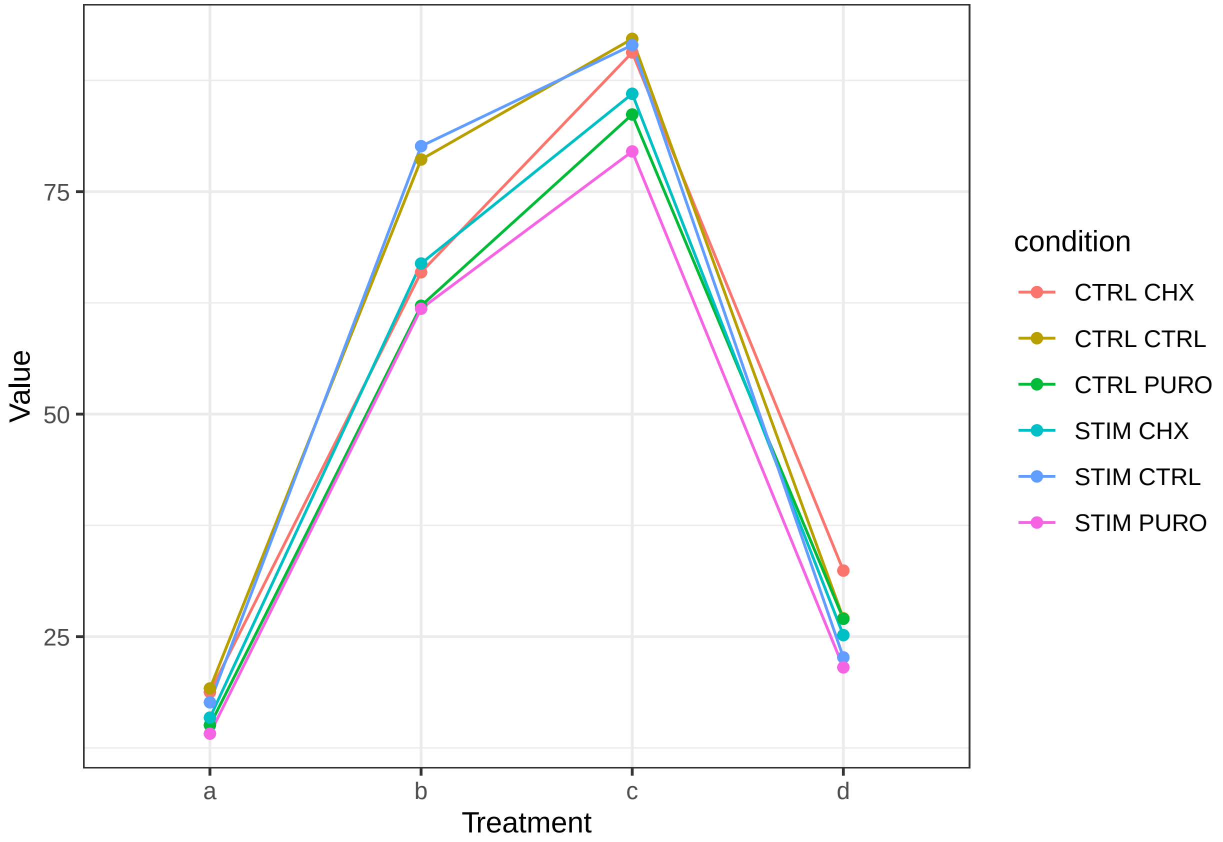


```
summary(pairs(emmeans(m5, "condition",by = "treatment",level = 0.95, infer = TRUE)))
```

```
## treatment = a:
##  contrast                estimate   SE   df  t.ratio p.value
##  CTRL CHX - CTRL CTRL     -0.4142 1.95 1095  -0.212  0.9999
##  CTRL CHX - CTRL PURO      3.7082 1.87 1095   1.984  0.3519
##  CTRL CHX - STIM CHX       2.8634 1.99 1095   1.438  0.7036
##  CTRL CHX - STIM CTRL      1.1447 1.92 1095   0.595  0.9914
##  CTRL CHX - STIM PURO      4.6651 1.96 1095   2.385  0.1623
##  CTRL CTRL - CTRL PURO     4.1224 1.90 1095   2.168  0.2535
##  CTRL CTRL - STIM CHX      3.2775 2.02 1095   1.622  0.5842
##  CTRL CTRL - STIM CTRL     1.5588 1.95 1095   0.798  0.9680
##  CTRL CTRL - STIM PURO     5.0792 1.99 1095   2.557  0.1089
##  CTRL PURO - STIM CHX     -0.8448 1.94 1095  -0.436  0.9980
##  CTRL PURO - STIM CTRL    -2.5636 1.87 1095  -1.372  0.7439
##  CTRL PURO - STIM PURO     0.9569 1.90 1095   0.503  0.9961
##  STIM CHX - STIM CTRL     -1.7187 1.99 1095  -0.864  0.9550
##  STIM CHX - STIM PURO      1.8017 2.02 1095   0.891  0.9486
##  STIM CTRL - STIM PURO     3.5204 1.95 1095   1.801  0.4654
##
## treatment = b:
##  contrast                estimate   SE   df  t.ratio p.value
##  CTRL CHX - CTRL CTRL    -12.6706 1.95 1095  -6.483  <.0001
##  CTRL CHX - CTRL PURO      3.7680 1.87 1095   2.016  0.3335
##  CTRL CHX - STIM CHX      -0.9835 1.99 1095  -0.494  0.9964
```

```
##  CTRL CHX - STIM CTRL  -14.1693 1.92 1095  -7.368  <.0001
##  CTRL CHX - STIM PURO    4.0923 1.96 1095   2.092  0.2920
##  CTRL CTRL - CTRL PURO  16.4385 1.90 1095   8.646  <.0001
##  CTRL CTRL - STIM CHX   11.6870 2.02 1095   5.783  <.0001
##  CTRL CTRL - STIM CTRL  -1.4987 1.95 1095  -0.767  0.9730
##  CTRL CTRL - STIM PURO  16.7629 1.99 1095   8.439  <.0001
##  CTRL PURO - STIM CHX   -4.7515 1.94 1095  -2.451  0.1398
##  CTRL PURO - STIM CTRL -17.9372 1.87 1095  -9.599  <.0001
##  CTRL PURO - STIM PURO   0.3244 1.90 1095   0.171  1.0000
##  STIM CHX - STIM CTRL  -13.1858 1.99 1095  -6.625  <.0001
##  STIM CHX - STIM PURO    5.0758 2.02 1095   2.512  0.1215
##  STIM CTRL - STIM PURO  18.2616 1.95 1095   9.343  <.0001
##
## treatment = c:
##  contrast              estimate   SE   df t.ratio p.value
##  CTRL CHX - CTRL CTRL   -1.5340 1.95 1095  -0.785  0.9701
##  CTRL CHX - CTRL PURO    6.9667 1.87 1095   3.728  0.0028
##  CTRL CHX - STIM CHX     4.6418 1.99 1095   2.331  0.1823
##  CTRL CHX - STIM CTRL   -0.8281 1.92 1095  -0.431  0.9981
##  CTRL CHX - STIM PURO   11.1187 1.96 1095   5.685  <.0001
##  CTRL CTRL - CTRL PURO   8.5006 1.90 1095   4.471  0.0001
##  CTRL CTRL - STIM CHX    6.1757 2.02 1095   3.056  0.0278
##  CTRL CTRL - STIM CTRL   0.7058 1.95 1095   0.361  0.9992
##  CTRL CTRL - STIM PURO  12.6527 1.99 1095   6.370  <.0001
##  CTRL PURO - STIM CHX   -2.3249 1.94 1095  -1.199  0.8372
##  CTRL PURO - STIM CTRL  -7.7948 1.87 1095  -4.172  0.0005
##  CTRL PURO - STIM PURO   4.1521 1.90 1095   2.183  0.2464
##  STIM CHX - STIM CTRL   -5.4699 1.99 1095  -2.748  0.0669
##  STIM CHX - STIM PURO    6.4770 2.02 1095   3.205  0.0174
##  STIM CTRL - STIM PURO  11.9469 1.95 1095   6.112  <.0001
##
## treatment = d:
##  contrast              estimate   SE   df t.ratio p.value
##  CTRL CHX - CTRL CTRL    5.3664 1.95 1095   2.746  0.0674
##  CTRL CHX - CTRL PURO    5.4187 1.87 1095   2.900  0.0440
##  CTRL CHX - STIM CHX     7.2548 1.99 1095   3.644  0.0038
##  CTRL CHX - STIM CTRL    9.7588 1.99 1095   4.903  <.0001
##  CTRL CHX - STIM PURO   10.8760 1.96 1095   5.561  <.0001
##  CTRL CTRL - CTRL PURO   0.0522 1.90 1095   0.027  1.0000
##  CTRL CTRL - STIM CHX    1.8883 2.02 1095   0.934  0.9376
##  CTRL CTRL - STIM CTRL   4.3924 2.02 1095   2.173  0.2511
##  CTRL CTRL - STIM PURO   5.5096 1.99 1095   2.774  0.0625
##  CTRL PURO - STIM CHX    1.8361 1.94 1095   0.947  0.9340
##  CTRL PURO - STIM CTRL   4.3401 1.94 1095   2.239  0.2208
##  CTRL PURO - STIM PURO   5.4574 1.90 1095   2.870  0.0480
##  STIM CHX - STIM CTRL    2.5040 2.06 1095   1.218  0.8282
##  STIM CHX - STIM PURO    3.6213 2.02 1095   1.792  0.4714
##  STIM CTRL - STIM PURO   1.1172 2.02 1095   0.552  0.9939
##
## Degrees-of-freedom method: kenward-roger
## P value adjustment: tukey method for comparing a family of 6 estimates
```

# OCR - Glycostress

```
seahorse6$time <- as.numeric(seahorse6$time)
ggplot(seahorse6, aes(time, value, colour=condition))+
  geom_point()+
  theme_bw()+
  xlab("Time (min)") +
  ylab('Value')+
  facet_wrap(~ind)
```

```
## Warning: Removed 12 rows containing missing values (‘geom_point()‘).
```

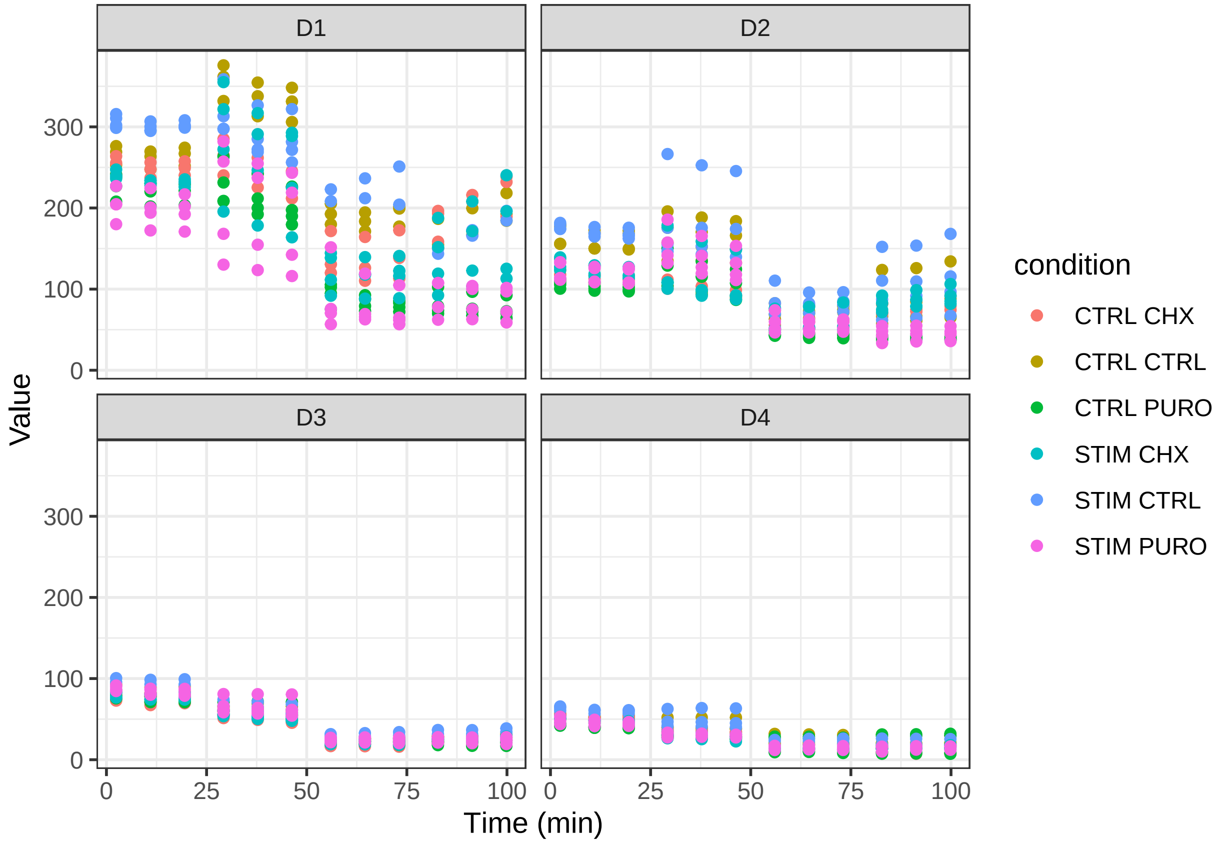


```
m6<-lmer(value ~ treatment*condition + (1|ind), data=seahorse6)
summary(m6)
```

```
## Linear mixed model fit by REML. t-tests use Satterthwaite's method [
## lmerModLmerTest]
## Formula: value ~ treatment * condition + (1 | ind)
##    Data: seahorse6
##
## REML criterion at convergence: 10527.3
##
## Scaled residuals:
```

```
##     Min      1Q  Median      3Q     Max
## -3.1220 -0.4565 -0.1066  0.5774  4.5595
##
## Random effects:
##  Groups   Name        Variance Std.Dev.
##  ind      (Intercept) 5535.5   74.40
##  Residual              901.4   30.02
## Number of obs: 1104, groups:  ind, 4
##
## Fixed effects:
##                              Estimate Std. Error       df t value Pr(>|t|)
## (Intercept)                   121.413     37.488    3.090   3.239  0.04593 *
## treatmentb                    -15.518      6.552 1077.000  -2.369  0.01803 *
## treatmentc                    -63.369      6.552 1077.000  -9.672  < 2e-16 ***
## treatmentd                    -43.590      6.552 1077.000  -6.653 4.55e-11 ***
## conditionCTRL CTRL             16.958      6.345 1077.000   2.673  0.00763 **
## conditionCTRL PURO            -10.653      6.345 1077.000  -1.679  0.09345 .
## conditionSTIM CHX               0.835      6.557 1077.002   0.127  0.89869
## conditionSTIM CTRL             32.044      6.257 1077.000   5.121 3.59e-07 ***
## conditionSTIM PURO             -7.938      6.347 1077.001  -1.251  0.21131
## treatmentb:conditionCTRL CTRL   20.421      8.971 1077.000   2.276  0.02302 *
## treatmentc:conditionCTRL CTRL    1.161      8.971 1077.000   0.129  0.89705
## treatmentd:conditionCTRL CTRL  -15.967      8.971 1077.000  -1.780  0.07539 .
## treatmentb:conditionCTRL PURO    7.785      8.971 1077.000   0.868  0.38572
## treatmentc:conditionCTRL PURO   -5.316      8.971 1077.000  -0.593  0.55358
## treatmentd:conditionCTRL PURO  -27.687      8.971 1077.000  -3.086  0.00208 **
## treatmentb:conditionSTIM CHX     9.866      9.265 1077.000   1.065  0.28718
## treatmentc:conditionSTIM CHX    -9.033      9.265 1077.000  -0.975  0.32984
## treatmentd:conditionSTIM CHX    -9.960      9.265 1077.000  -1.075  0.28261
## treatmentb:conditionSTIM CTRL    5.969      8.847 1077.000   0.675  0.50000
## treatmentc:conditionSTIM CTRL   -7.080      8.982 1077.001  -0.788  0.43073
## treatmentd:conditionSTIM CTRL  -27.168      8.982 1077.001  -3.025  0.00255 **
## treatmentb:conditionSTIM PURO   10.249      8.971 1077.000   1.142  0.25351
## treatmentc:conditionSTIM PURO   -6.689      8.971 1077.000  -0.746  0.45606
## treatmentd:conditionSTIM PURO  -27.697      8.971 1077.000  -3.087  0.00207 **
## ---
## Signif. codes:  0 '***' 0.001 '**' 0.01 '*' 0.05 '.' 0.1 ' ' 1

##
## Correlation matrix not shown by default, as p = 24 > 12.
## Use print(x, correlation=TRUE)  or
##     vcov(x)        if you need it
```

```
summary(emmeans(m6, pairwise ~ condition|treatment, infer=T))
```

```
## $emmeans
## treatment = a:
##  condition emmean   SE   df lower.CL upper.CL t.ratio p.value
##  CTRL CHX   121.4 37.5 3.09    4.054      239   3.239  0.0459
##  CTRL CTRL  138.4 37.5 3.08   20.880      256   3.695  0.0329
##  CTRL PURO  110.8 37.5 3.08   -6.732      228   2.957  0.0578
##  STIM CHX   122.2 37.5 3.09    4.889      240   3.261  0.0451
##  STIM CTRL  153.5 37.4 3.07   35.909      271   4.099  0.0251
```

```
##  STIM PURO  113.5 37.5 3.08   -4.017      231   3.030  0.0545
##
## treatment = b:
##  condition emmean   SE   df lower.CL upper.CL t.ratio p.value
##  CTRL CHX   105.9 37.5 3.09  -11.464      223   2.825  0.0642
##  CTRL CTRL  143.3 37.5 3.08   25.783      261   3.825  0.0301
##  CTRL PURO  103.0 37.5 3.08  -14.466      221   2.751  0.0687
##  STIM CHX   116.6 37.5 3.09   -0.763      234   3.110  0.0508
##  STIM CTRL  143.9 37.4 3.07   26.360      261   3.844  0.0298
##  STIM PURO  108.2 37.5 3.08   -9.286      226   2.889  0.0611
##
## treatment = c:
##  condition emmean   SE   df lower.CL upper.CL t.ratio p.value
##  CTRL CHX    58.0 37.5 3.09  -59.314      175   1.548  0.2167
##  CTRL CTRL   76.2 37.5 3.08  -41.327      194   2.034  0.1325
##  CTRL PURO   42.1 37.5 3.08  -75.417      160   1.123  0.3412
##  STIM CHX    49.8 37.5 3.09  -67.512      167   1.330  0.2733
##  STIM CTRL   83.0 37.5 3.08  -34.419      200   2.215  0.1111
##  STIM PURO   43.4 37.5 3.08  -74.074      161   1.159  0.3283
##
## treatment = d:
##  condition emmean   SE   df lower.CL upper.CL t.ratio p.value
##  CTRL CHX    77.8 37.5 3.09  -39.535      195   2.076  0.1268
##  CTRL CTRL   78.8 37.5 3.08  -38.677      196   2.104  0.1237
##  CTRL PURO   39.5 37.5 3.08  -78.009      157   1.054  0.3674
##  STIM CHX    68.7 37.5 3.09  -48.661      186   1.833  0.1616
##  STIM CTRL   82.7 37.5 3.08  -34.728      200   2.207  0.1120
##  STIM PURO   42.2 37.5 3.08  -75.303      160   1.126  0.3401
##
## Degrees-of-freedom method: kenward-roger
## Confidence level used: 0.95
##
## $contrasts
## treatment = a:
##  contrast              estimate   SE   df lower.CL upper.CL t.ratio p.value
##  CTRL CHX - CTRL CTRL   -16.958 6.34 1077   -35.07   1.1548  -2.673  0.0816
##  CTRL CHX - CTRL PURO    10.653 6.34 1077    -7.46  28.7668   1.679  0.5461
##  CTRL CHX - STIM CHX     -0.835 6.56 1077   -19.55  17.8837  -0.127  1.0000
##  CTRL CHX - STIM CTRL   -32.043 6.26 1077   -49.91 -14.1810  -5.121  <.0001
##  CTRL CHX - STIM PURO     7.938 6.35 1077   -10.18  26.0583   1.251  0.8115
##  CTRL CTRL - CTRL PURO   27.611 6.13 1077    10.11  45.1130   4.504  0.0001
##  CTRL CTRL - STIM CHX    16.123 6.35 1077    -2.00  34.2493   2.539  0.1137
##  CTRL CTRL - STIM CTRL  -15.085 6.04 1077   -32.32   2.1539  -2.498  0.1255
##  CTRL CTRL - STIM PURO   24.897 6.13 1077     7.39  42.3989   4.061  0.0007
##  CTRL PURO - STIM CHX   -11.488 6.34 1077   -29.60   6.6259  -1.811  0.4593
##  CTRL PURO - STIM CTRL  -42.696 6.04 1077   -59.94 -25.4579  -7.071  <.0001
##  CTRL PURO - STIM PURO   -2.715 6.13 1077   -20.22  14.7871  -0.443  0.9979
##  STIM CHX - STIM CTRL   -31.209 6.26 1077   -49.08 -13.3402  -4.986  <.0001
##  STIM CHX - STIM PURO     8.773 6.35 1077    -9.35  26.8922   1.382  0.7377
##  STIM CTRL - STIM PURO   39.982 6.04 1077    22.74  57.2210   6.621  <.0001
##
## treatment = b:
##  contrast              estimate   SE   df lower.CL upper.CL t.ratio p.value
##  CTRL CHX - CTRL CTRL   -37.379 6.34 1077   -55.49 -19.2664  -5.892  <.0001
```

```
##  CTRL CHX - CTRL PURO     2.868 6.34 1077   -15.25  20.9820   0.452  0.9976
##  CTRL CHX - STIM CHX    -10.701 6.56 1077   -29.42   8.0175  -1.632  0.5774
##  CTRL CHX - STIM CTRL   -38.013 6.26 1077   -55.88 -20.1504  -6.075  <.0001
##  CTRL CHX - STIM PURO    -2.311 6.35 1077   -20.43  15.8090  -0.364  0.9992
##  CTRL CTRL - CTRL PURO   40.248 6.13 1077    22.75  57.7495   6.565  <.0001
##  CTRL CTRL - STIM CHX    26.678 6.35 1077     8.55  44.8043   4.202  0.0004
##  CTRL CTRL - STIM CTRL   -0.633 6.04 1077   -17.87  16.6058  -0.105  1.0000
##  CTRL CTRL - STIM PURO   35.068 6.13 1077    17.57  52.5708   5.720  <.0001
##  CTRL PURO - STIM CHX   -13.569 6.34 1077   -31.68   4.5444  -2.139  0.2682
##  CTRL PURO - STIM CTRL  -40.881 6.04 1077   -58.12 -23.6424  -6.770  <.0001
##  CTRL PURO - STIM PURO   -5.179 6.13 1077   -22.68  12.3225  -0.845  0.9590
##  STIM CHX - STIM CTRL   -27.312 6.26 1077   -45.18  -9.4433  -4.364  0.0002
##  STIM CHX - STIM PURO     8.390 6.35 1077    -9.73  26.5092   1.322  0.7729
##  STIM CTRL - STIM PURO   35.702 6.04 1077    18.46  52.9410   5.912  <.0001
##
## treatment = c:
##  contrast              estimate   SE   df lower.CL upper.CL t.ratio p.value
##  CTRL CHX - CTRL CTRL   -18.119 6.34 1077   -36.23  -0.0063  -2.856  0.0499
##  CTRL CHX - CTRL PURO    15.969 6.34 1077    -2.14  34.0831   2.517  0.1200
##  CTRL CHX - STIM CHX      8.198 6.56 1077   -10.52  26.9163   1.250  0.8118
##  CTRL CHX - STIM CTRL   -24.963 6.44 1077   -43.36  -6.5649  -3.874  0.0016
##  CTRL CHX - STIM PURO    14.627 6.35 1077    -3.49  32.7474   2.305  0.1930
##  CTRL CTRL - CTRL PURO   34.089 6.13 1077    16.59  51.5904   5.560  <.0001
##  CTRL CTRL - STIM CHX    26.317 6.35 1077     8.19  44.4430   4.145  0.0005
##  CTRL CTRL - STIM CTRL   -6.844 6.23 1077   -24.63  10.9441  -1.098  0.8821
##  CTRL CTRL - STIM PURO   32.747 6.13 1077    15.24  50.2491   5.341  <.0001
##  CTRL PURO - STIM CHX    -7.772 6.34 1077   -25.89  10.3422  -1.225  0.8247
##  CTRL PURO - STIM CTRL  -40.933 6.24 1077   -58.73 -23.1312  -6.564  <.0001
##  CTRL PURO - STIM PURO   -1.342 6.13 1077   -18.84  16.1599  -0.219  0.9999
##  STIM CHX - STIM CTRL   -33.161 6.45 1077   -51.58 -14.7414  -5.140  <.0001
##  STIM CHX - STIM PURO     6.430 6.35 1077   -11.69  24.5487   1.013  0.9137
##  STIM CTRL - STIM PURO   39.591 6.24 1077    21.79  57.3928   6.349  <.0001
##
## treatment = d:
##  contrast              estimate   SE   df lower.CL upper.CL t.ratio p.value
##  CTRL CHX - CTRL CTRL    -0.991 6.34 1077   -19.10  17.1217  -0.156  1.0000
##  CTRL CHX - CTRL PURO    38.340 6.34 1077    20.23  56.4535   6.043  <.0001
##  CTRL CHX - STIM CHX      9.126 6.56 1077    -9.59  27.8442   1.392  0.7321
##  CTRL CHX - STIM CTRL    -4.876 6.44 1077   -23.27  13.5228  -0.757  0.9745
##  CTRL CHX - STIM PURO    35.636 6.35 1077    17.52  53.7554   5.615  <.0001
##  CTRL CTRL - CTRL PURO   39.331 6.13 1077    21.83  56.8327   6.416  <.0001
##  CTRL CTRL - STIM CHX    10.117 6.35 1077    -8.01  28.2429   1.593  0.6031
##  CTRL CTRL - STIM CTRL   -3.884 6.23 1077   -21.67  13.9038  -0.623  0.9893
##  CTRL CTRL - STIM PURO   36.627 6.13 1077    19.12  54.1291   5.974  <.0001
##  CTRL PURO - STIM CHX   -29.214 6.34 1077   -47.33 -11.1003  -4.604  0.0001
##  CTRL PURO - STIM CTRL  -43.215 6.24 1077   -61.02 -25.4139  -6.931  <.0001
##  CTRL PURO - STIM PURO   -2.704 6.13 1077   -20.21  14.7975  -0.441  0.9979
##  STIM CHX - STIM CTRL   -14.001 6.45 1077   -32.42   4.4185  -2.170  0.2527
##  STIM CHX - STIM PURO    26.510 6.35 1077     8.39  44.6288   4.177  0.0005
##  STIM CTRL - STIM PURO   40.511 6.24 1077    22.71  58.3130   6.497  <.0001
##
## Degrees-of-freedom method: kenward-roger
## Confidence level used: 0.95
## Conf-level adjustment: tukey method for comparing a family of 6 estimates
```

```
## P value adjustment: tukey method for comparing a family of 6 estimates
```

```
emmip(m6, condition ~ treatment) +
  theme_bw()+
  xlab('Treatment') +
  ylab('Value')
```

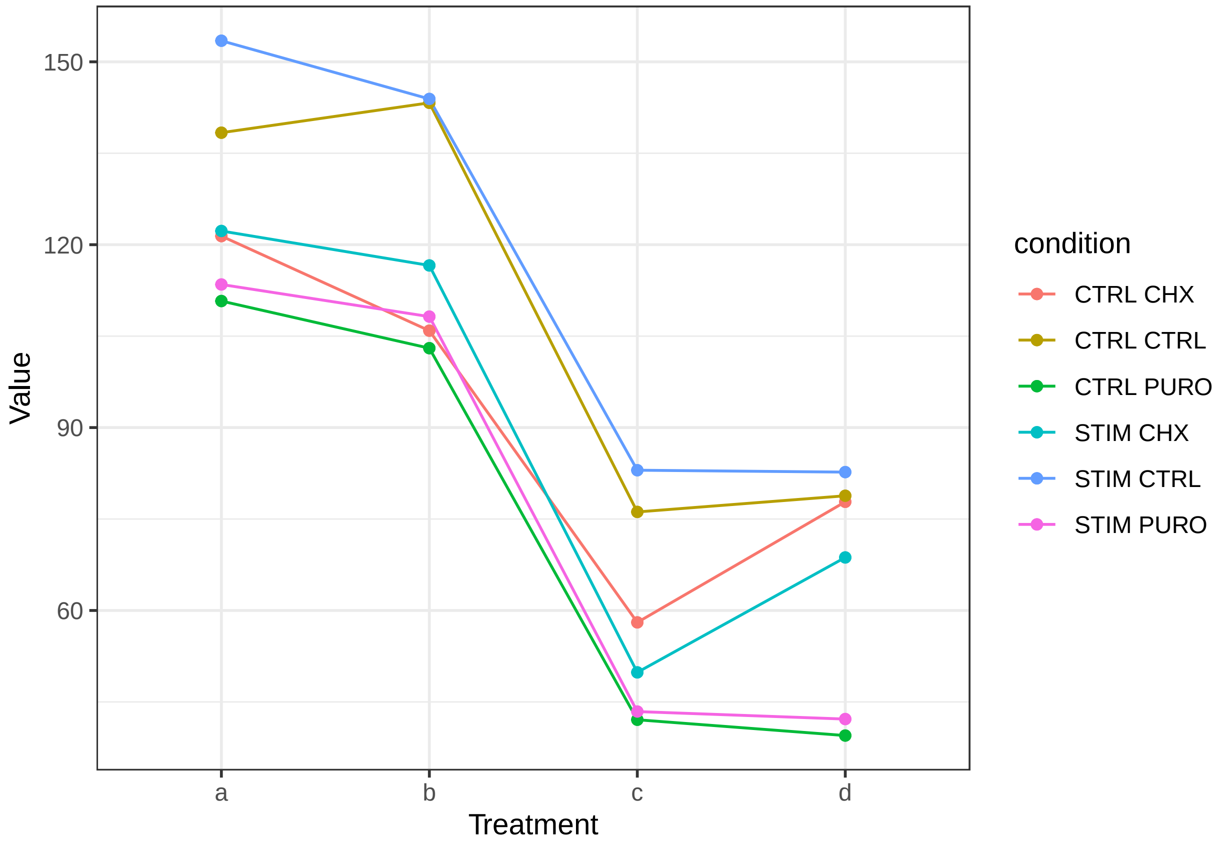


```
summary(pairs(emmeans(m6, "condition",by = "treatment",level = 0.95, infer = TRUE)))
```

```
## treatment = a:
##  contrast                estimate   SE   df t.ratio p.value
##  CTRL CHX - CTRL CTRL     -16.958 6.34 1077  -2.673  0.0816
##  CTRL CHX - CTRL PURO      10.653 6.34 1077   1.679  0.5461
##  CTRL CHX - STIM CHX       -0.835 6.56 1077  -0.127  1.0000
##  CTRL CHX - STIM CTRL     -32.043 6.26 1077  -5.121  <.0001
##  CTRL CHX - STIM PURO       7.938 6.35 1077   1.251  0.8115
##  CTRL CTRL - CTRL PURO     27.611 6.13 1077   4.504  0.0001
##  CTRL CTRL - STIM CHX      16.123 6.35 1077   2.539  0.1137
##  CTRL CTRL - STIM CTRL    -15.085 6.04 1077  -2.498  0.1255
##  CTRL CTRL - STIM PURO     24.897 6.13 1077   4.061  0.0007
##  CTRL PURO - STIM CHX     -11.488 6.34 1077  -1.811  0.4593
##  CTRL PURO - STIM CTRL    -42.696 6.04 1077  -7.071  <.0001
##  CTRL PURO - STIM PURO     -2.715 6.13 1077  -0.443  0.9979
##  STIM CHX - STIM CTRL     -31.209 6.26 1077  -4.986  <.0001
##  STIM CHX - STIM PURO       8.773 6.35 1077   1.382  0.7377
```

```
##  STIM CTRL - STIM PURO   39.982 6.04 1077   6.621  <.0001
##
## treatment = b:
##  contrast              estimate   SE   df t.ratio p.value
##  CTRL CHX - CTRL CTRL   -37.379 6.34 1077  -5.892  <.0001
##  CTRL CHX - CTRL PURO     2.868 6.34 1077   0.452  0.9976
##  CTRL CHX - STIM CHX    -10.701 6.56 1077  -1.632  0.5774
##  CTRL CHX - STIM CTRL   -38.013 6.26 1077  -6.075  <.0001
##  CTRL CHX - STIM PURO    -2.311 6.35 1077  -0.364  0.9992
##  CTRL CTRL - CTRL PURO   40.248 6.13 1077   6.565  <.0001
##  CTRL CTRL - STIM CHX    26.678 6.35 1077   4.202  0.0004
##  CTRL CTRL - STIM CTRL   -0.633 6.04 1077  -0.105  1.0000
##  CTRL CTRL - STIM PURO   35.068 6.13 1077   5.720  <.0001
##  CTRL PURO - STIM CHX   -13.569 6.34 1077  -2.139  0.2682
##  CTRL PURO - STIM CTRL  -40.881 6.04 1077  -6.770  <.0001
##  CTRL PURO - STIM PURO   -5.179 6.13 1077  -0.845  0.9590
##  STIM CHX - STIM CTRL   -27.312 6.26 1077  -4.364  0.0002
##  STIM CHX - STIM PURO     8.390 6.35 1077   1.322  0.7729
##  STIM CTRL - STIM PURO   35.702 6.04 1077   5.912  <.0001
##
## treatment = c:
##  contrast              estimate   SE   df t.ratio p.value
##  CTRL CHX - CTRL CTRL   -18.119 6.34 1077  -2.856  0.0499
##  CTRL CHX - CTRL PURO    15.969 6.34 1077   2.517  0.1200
##  CTRL CHX - STIM CHX      8.198 6.56 1077   1.250  0.8118
##  CTRL CHX - STIM CTRL   -24.963 6.44 1077  -3.874  0.0016
##  CTRL CHX - STIM PURO    14.627 6.35 1077   2.305  0.1930
##  CTRL CTRL - CTRL PURO   34.089 6.13 1077   5.560  <.0001
##  CTRL CTRL - STIM CHX    26.317 6.35 1077   4.145  0.0005
##  CTRL CTRL - STIM CTRL   -6.844 6.23 1077  -1.098  0.8821
##  CTRL CTRL - STIM PURO   32.747 6.13 1077   5.341  <.0001
##  CTRL PURO - STIM CHX    -7.772 6.34 1077  -1.225  0.8247
##  CTRL PURO - STIM CTRL  -40.933 6.24 1077  -6.564  <.0001
##  CTRL PURO - STIM PURO   -1.342 6.13 1077  -0.219  0.9999
##  STIM CHX - STIM CTRL   -33.161 6.45 1077  -5.140  <.0001
##  STIM CHX - STIM PURO     6.430 6.35 1077   1.013  0.9137
##  STIM CTRL - STIM PURO   39.591 6.24 1077   6.349  <.0001
##
## treatment = d:
##  contrast              estimate   SE   df t.ratio p.value
##  CTRL CHX - CTRL CTRL    -0.991 6.34 1077  -0.156  1.0000
##  CTRL CHX - CTRL PURO    38.340 6.34 1077   6.043  <.0001
##  CTRL CHX - STIM CHX      9.126 6.56 1077   1.392  0.7321
##  CTRL CHX - STIM CTRL    -4.876 6.44 1077  -0.757  0.9745
##  CTRL CHX - STIM PURO    35.636 6.35 1077   5.615  <.0001
##  CTRL CTRL - CTRL PURO   39.331 6.13 1077   6.416  <.0001
##  CTRL CTRL - STIM CHX    10.117 6.35 1077   1.593  0.6031
##  CTRL CTRL - STIM CTRL   -3.884 6.23 1077  -0.623  0.9893
##  CTRL CTRL - STIM PURO   36.627 6.13 1077   5.974  <.0001
##  CTRL PURO - STIM CHX   -29.214 6.34 1077  -4.604  0.0001
##  CTRL PURO - STIM CTRL  -43.215 6.24 1077  -6.931  <.0001
##  CTRL PURO - STIM PURO   -2.704 6.13 1077  -0.441  0.9979
##  STIM CHX - STIM CTRL   -14.001 6.45 1077  -2.170  0.2527
##  STIM CHX - STIM PURO    26.510 6.35 1077   4.177  0.0005
```

```
##  STIM CTRL - STIM PURO   40.511 6.24 1077   6.497  <.0001
##
## Degrees-of-freedom method: kenward-roger
## P value adjustment: tukey method for comparing a family of 6 estimates
```

## ECAR - MitoStress

```
seahorse7$time <- as.numeric(seahorse7$time)
ggplot(seahorse7, aes(time, value, colour=condition))+
  geom_point()+
  theme_bw()+
  xlab("Time (min)") +
  ylab('Value')+
  facet_wrap(~ind)
```

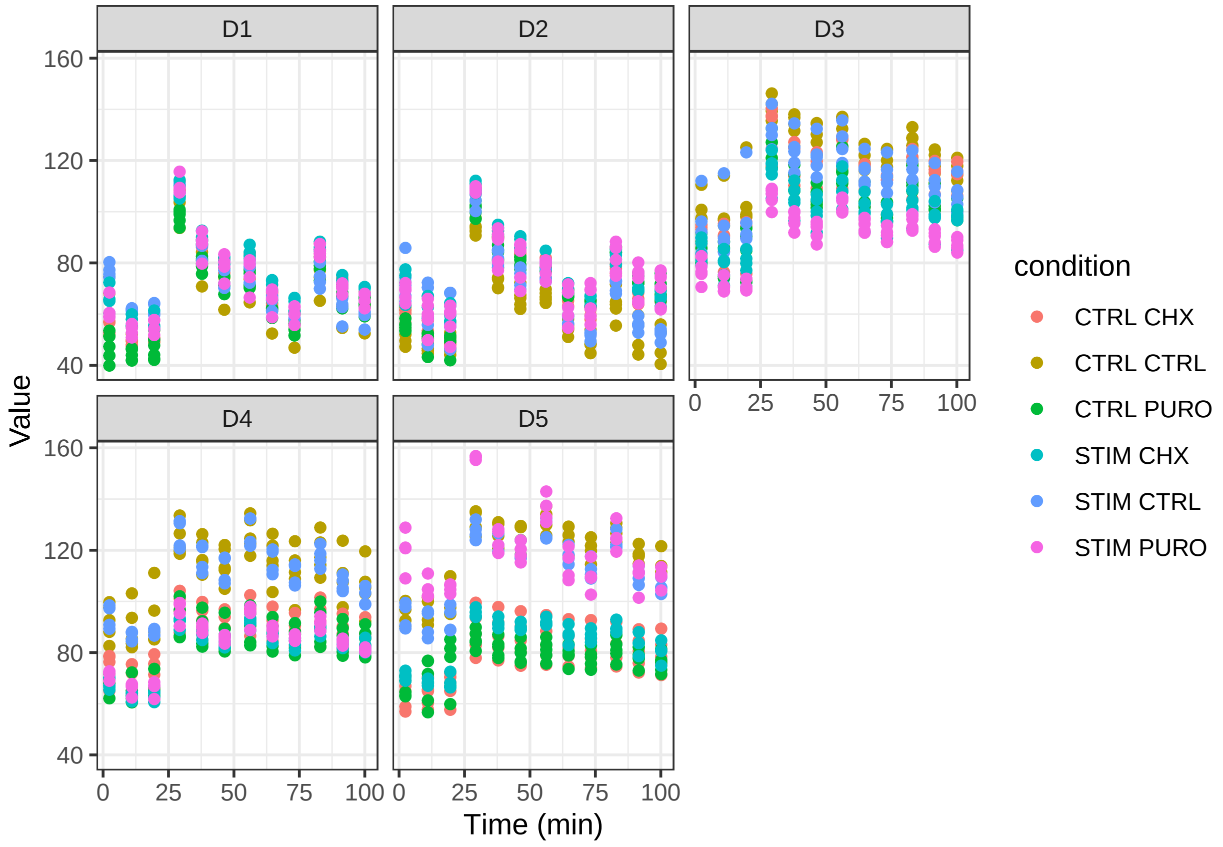


```
m7<-lmer(value ~ treatment*condition + (1|ind), data=seahorse7)
summary(m7)
```

```
## Linear mixed model fit by REML. t-tests use Satterthwaite's method [
## lmerModLmerTest]
## Formula: value ~ treatment * condition + (1 | ind)
##    Data: seahorse7
```

```
##
## REML criterion at convergence: 13156.1
##
## Scaled residuals:
##     Min      1Q  Median      3Q     Max
## -2.8643 -0.6470 -0.0177  0.6403  3.5059
##
## Random effects:
##  Groups   Name        Variance Std.Dev.
##  ind      (Intercept) 244.5    15.64
##  Residual             171.8    13.11
## Number of obs: 1656, groups:  ind, 5
##
## Fixed effects:
##                                Estimate Std. Error        df t value Pr(>|t|)
## (Intercept)                     66.0134     7.1686    4.3993   9.209 0.000488
## treatmentb                      31.7541     2.2313 1628.0003  14.231  < 2e-16
## treatmentc                      19.5166     2.2313 1628.0003   8.747  < 2e-16
## treatmentd                      19.2519     2.2313 1628.0003   8.628  < 2e-16
## conditionCTRL CTRL              13.9699     2.2572 1628.0049   6.189 7.65e-10
## conditionCTRL PURO              -2.6484     2.1662 1628.0020  -1.223 0.221669
## conditionSTIM CHX                1.3502     2.2571 1628.0043   0.598 0.549788
## conditionSTIM CTRL              15.4464     2.2567 1628.0016   6.845 1.08e-11
## conditionSTIM PURO               7.4538     2.2317 1628.0029   3.340 0.000857
## treatmentb:conditionCTRL CTRL   -2.8523     3.1912 1628.0003  -0.894 0.371551
## treatmentc:conditionCTRL CTRL   -1.2905     3.1912 1628.0003  -0.404 0.685969
## treatmentd:conditionCTRL CTRL   -4.5394     3.1912 1628.0003  -1.422 0.155081
## treatmentb:conditionCTRL PURO   -2.9834     3.0632 1628.0003  -0.974 0.330214
## treatmentc:conditionCTRL PURO   -1.3759     3.0632 1628.0003  -0.449 0.653371
## treatmentd:conditionCTRL PURO    0.8755     3.0632 1628.0003   0.286 0.775052
## treatmentb:conditionSTIM CHX    -4.5565     3.1912 1628.0003  -1.428 0.153527
## treatmentc:conditionSTIM CHX    -2.2750     3.1912 1628.0003  -0.713 0.476015
## treatmentd:conditionSTIM CHX    -3.5985     3.1912 1628.0003  -1.128 0.259637
## treatmentb:conditionSTIM CTRL   -4.9161     3.1912 1628.0003  -1.541 0.123629
## treatmentc:conditionSTIM CTRL   -4.7641     3.1912 1628.0003  -1.493 0.135659
## treatmentd:conditionSTIM CTRL   -9.8138     3.1912 1628.0003  -3.075 0.002138
## treatmentb:conditionSTIM PURO   -4.2237     3.1555 1628.0003  -1.339 0.180913
## treatmentc:conditionSTIM PURO   -5.2507     3.1555 1628.0003  -1.664 0.096313
## treatmentd:conditionSTIM PURO   -5.0811     3.1555 1628.0003  -1.610 0.107542
##
## (Intercept)                   ***
## treatmentb                    ***
## treatmentc                    ***
## treatmentd                    ***
## conditionCTRL CTRL            ***
## conditionCTRL PURO
## conditionSTIM CHX
## conditionSTIM CTRL            ***
## conditionSTIM PURO            ***
## treatmentb:conditionCTRL CTRL
## treatmentc:conditionCTRL CTRL
## treatmentd:conditionCTRL CTRL
## treatmentb:conditionCTRL PURO
## treatmentc:conditionCTRL PURO
```

```
## treatmentd:conditionCTRL PURO
## treatmentb:conditionSTIM CHX
## treatmentc:conditionSTIM CHX
## treatmentd:conditionSTIM CHX
## treatmentb:conditionSTIM CTRL
## treatmentc:conditionSTIM CTRL
## treatmentd:conditionSTIM CTRL **
## treatmentb:conditionSTIM PURO
## treatmentc:conditionSTIM PURO .
## treatmentd:conditionSTIM PURO
## ---
## Signif. codes:  0 '***' 0.001 '**' 0.01 '*' 0.05 '.' 0.1 ' ' 1

##
## Correlation matrix not shown by default, as p = 24 > 12.
## Use print(x, correlation=TRUE)  or
##     vcov(x)        if you need it
```

```
summary(emmeans(m7, pairwise ~ condition|treatment, infer=T))
```

```
## $emmeans
## treatment = a:
##  condition emmean   SE   df lower.CL upper.CL t.ratio p.value
##  CTRL CHX    66.0 7.17 4.40     46.8     85.2   9.209  0.0005
##  CTRL CTRL   80.0 7.18 4.42     60.8     99.2  11.145  0.0002
##  CTRL PURO   63.4 7.15 4.35     44.1     82.6   8.864  0.0006
##  STIM CHX    67.4 7.18 4.42     48.2     86.6   9.387  0.0004
##  STIM CTRL   81.5 7.18 4.42     62.3    100.7  11.351  0.0002
##  STIM PURO   73.5 7.17 4.40     54.3     92.7  10.248  0.0003
##
## treatment = b:
##  condition emmean   SE   df lower.CL upper.CL t.ratio p.value
##  CTRL CHX    97.8 7.17 4.40     78.6    117.0  13.638  0.0001
##  CTRL CTRL  108.9 7.18 4.42     89.7    128.1  15.172  0.0001
##  CTRL PURO   92.1 7.15 4.35     72.9    111.4  12.889  0.0001
##  STIM CHX    94.6 7.18 4.42     75.4    113.8  13.177  0.0001
##  STIM CTRL  108.3 7.18 4.42     89.1    127.5  15.091  0.0001
##  STIM PURO  101.0 7.17 4.40     81.8    120.2  14.089  0.0001
##
## treatment = c:
##  condition emmean   SE   df lower.CL upper.CL t.ratio p.value
##  CTRL CHX    85.5 7.17 4.40     66.3    104.7  11.931  0.0002
##  CTRL CTRL   98.2 7.18 4.42     79.0    117.4  13.685  0.0001
##  CTRL PURO   81.5 7.15 4.35     62.3    100.7  11.402  0.0002
##  STIM CHX    84.6 7.18 4.42     65.4    103.8  11.789  0.0002
##  STIM CTRL   96.2 7.18 4.42     77.0    115.4  13.407  0.0001
##  STIM PURO   87.7 7.17 4.40     68.5    106.9  12.238  0.0001
##
## treatment = d:
##  condition emmean   SE   df lower.CL upper.CL t.ratio p.value
##  CTRL CHX    85.3 7.17 4.40     66.1    104.5  11.894  0.0002
##  CTRL CTRL   94.7 7.18 4.42     75.5    113.9  13.195  0.0001
##  CTRL PURO   83.5 7.15 4.35     64.3    102.7  11.680  0.0002
```

```
##  STIM CHX    83.0 7.18 4.42     63.8    102.2  11.568  0.0002
##  STIM CTRL   90.9 7.18 4.42     71.7    110.1  12.666  0.0001
##  STIM PURO   87.6 7.17 4.40     68.4    106.8  12.225  0.0001
##
## Degrees-of-freedom method: kenward-roger
## Confidence level used: 0.95
##
## $contrasts
## treatment = a:
##  contrast              estimate   SE   df lower.CL upper.CL t.ratio p.value
##  CTRL CHX - CTRL CTRL   -13.970 2.26 1628 -20.4099  -7.5298  -6.189  <.0001
##  CTRL CHX - CTRL PURO     2.648 2.17 1628  -3.5321   8.8289   1.223  0.8259
##  CTRL CHX - STIM CHX     -1.350 2.26 1628  -7.7901   5.0897  -0.598  0.9912
##  CTRL CHX - STIM CTRL   -15.446 2.26 1628 -21.8850  -9.0078  -6.845  <.0001
##  CTRL CHX - STIM PURO    -7.454 2.23 1628 -13.8211  -1.0864  -3.340  0.0111
##  CTRL CTRL - CTRL PURO   16.618 2.19 1628  10.3623  22.8742   7.579  <.0001
##  CTRL CTRL - STIM CHX    12.620 2.28 1628   6.1091  19.1302   5.530  <.0001
##  CTRL CTRL - STIM CTRL   -1.477 2.28 1628  -7.9872   5.0341  -0.647  0.9873
##  CTRL CTRL - STIM PURO    6.516 2.26 1628   0.0761  12.9560   2.887  0.0455
##  CTRL PURO - STIM CHX    -3.999 2.19 1628 -10.2541   2.2569  -1.824  0.4506
##  CTRL PURO - STIM CTRL  -18.095 2.19 1628 -24.3492 -11.8405  -8.254  <.0001
##  CTRL PURO - STIM PURO  -10.102 2.17 1628 -16.2828  -3.9215  -4.663  <.0001
##  STIM CHX - STIM CTRL   -14.096 2.28 1628 -20.6067  -7.5857  -6.177  <.0001
##  STIM CHX - STIM PURO    -6.104 2.26 1628 -12.5445   0.3374  -2.704  0.0750
##  STIM CTRL - STIM PURO    7.993 2.26 1628   1.5542  14.4311   3.542  0.0055
##
## treatment = b:
##  contrast              estimate   SE   df lower.CL upper.CL t.ratio p.value
##  CTRL CHX - CTRL CTRL   -11.118 2.26 1628 -17.5576  -4.6775  -4.925  <.0001
##  CTRL CHX - CTRL PURO     5.632 2.17 1628  -0.5487  11.8123   2.600  0.0978
##  CTRL CHX - STIM CHX      3.206 2.26 1628  -3.2336   9.6462   1.421  0.7146
##  CTRL CHX - STIM CTRL   -10.530 2.26 1628 -16.9690  -4.0918  -4.666  <.0001
##  CTRL CHX - STIM PURO    -3.230 2.23 1628  -9.5974   3.1373  -1.447  0.6979
##  CTRL CTRL - CTRL PURO   16.749 2.19 1628  10.4934  23.0052   7.639  <.0001
##  CTRL CTRL - STIM CHX    14.324 2.28 1628   7.8132  20.8344   6.277  <.0001
##  CTRL CTRL - STIM CTRL    0.587 2.28 1628  -5.9235   7.0978   0.257  0.9998
##  CTRL CTRL - STIM PURO    7.887 2.26 1628   1.4475  14.3274   3.494  0.0065
##  CTRL PURO - STIM CHX    -2.426 2.19 1628  -8.6810   3.8300  -1.106  0.8789
##  CTRL PURO - STIM CTRL  -16.162 2.19 1628 -22.4165  -9.9078  -7.373  <.0001
##  CTRL PURO - STIM PURO   -8.862 2.17 1628 -15.0425  -2.6812  -4.091  0.0006
##  STIM CHX - STIM CTRL   -13.737 2.28 1628 -20.2472  -7.2262  -6.020  <.0001
##  STIM CHX - STIM PURO    -6.436 2.26 1628 -12.8773   0.0046  -2.851  0.0503
##  STIM CTRL - STIM PURO    7.300 2.26 1628   0.8618  13.7388   3.235  0.0157
##
## treatment = c:
##  contrast              estimate   SE   df lower.CL upper.CL t.ratio p.value
##  CTRL CHX - CTRL CTRL   -12.679 2.26 1628 -19.1194  -6.2393  -5.617  <.0001
##  CTRL CHX - CTRL PURO     4.024 2.17 1628  -2.1563  10.2047   1.858  0.4289
##  CTRL CHX - STIM CHX      0.925 2.26 1628  -5.5152   7.3646   0.410  0.9985
##  CTRL CHX - STIM CTRL   -10.682 2.26 1628 -17.1210  -4.2437  -4.734  <.0001
##  CTRL CHX - STIM PURO    -2.203 2.23 1628  -8.5705   4.1642  -0.987  0.9221
##  CTRL CTRL - CTRL PURO   16.704 2.19 1628  10.4477  22.9595   7.618  <.0001
##  CTRL CTRL - STIM CHX    13.604 2.28 1628   7.0935  20.1147   5.962  <.0001
##  CTRL CTRL - STIM CTRL    1.997 2.28 1628  -4.5137   8.5077   0.875  0.9524
```

```
##  CTRL CTRL - STIM PURO   10.476 2.26 1628   4.0363  16.9162   4.641  0.0001
##  CTRL PURO - STIM CHX    -3.099 2.19 1628  -9.3550   3.1560  -1.414  0.7188
##  CTRL PURO - STIM CTRL  -14.707 2.19 1628 -20.9609  -8.4522  -6.709  <.0001
##  CTRL PURO - STIM PURO   -6.227 2.17 1628 -12.4080  -0.0467  -2.875  0.0470
##  STIM CHX - STIM CTRL   -11.607 2.28 1628 -18.1176  -5.0966  -5.087  <.0001
##  STIM CHX - STIM PURO    -3.128 2.26 1628  -9.5688   3.3131  -1.386  0.7358
##  STIM CTRL - STIM PURO    8.479 2.26 1628   2.0407  14.9177   3.757  0.0024
##
## treatment = d:
##  contrast              estimate   SE   df lower.CL upper.CL t.ratio p.value
##  CTRL CHX - CTRL CTRL    -9.430 2.26 1628 -15.8706  -2.9904  -4.178  0.0004
##  CTRL CHX - CTRL PURO     1.773 2.17 1628  -4.4076   7.9534   0.818  0.9642
##  CTRL CHX - STIM CHX      2.248 2.26 1628  -4.1916   8.6882   0.996  0.9193
##  CTRL CHX - STIM CTRL    -5.633 2.26 1628 -12.0712   0.8060  -2.496  0.1258
##  CTRL CHX - STIM PURO    -2.373 2.23 1628  -8.7400   3.9947  -1.063  0.8959
##  CTRL CTRL - CTRL PURO   11.203 2.19 1628   4.9474  17.4593   5.109  <.0001
##  CTRL CTRL - STIM CHX    11.679 2.28 1628   5.1682  18.1894   5.118  <.0001
##  CTRL CTRL - STIM CTRL    3.798 2.28 1628  -2.7128  10.3085   1.664  0.5558
##  CTRL CTRL - STIM PURO    7.058 2.26 1628   0.6179  13.4978   3.127  0.0222
##  CTRL PURO - STIM CHX     0.475 2.19 1628  -5.7800   6.7309   0.217  0.9999
##  CTRL PURO - STIM CTRL   -7.405 2.19 1628 -13.6598  -1.1511  -3.378  0.0097
##  CTRL PURO - STIM PURO   -4.146 2.17 1628 -10.3261   2.0351  -1.914  0.3940
##  STIM CHX - STIM CTRL    -7.881 2.28 1628 -14.3914  -1.3704  -3.454  0.0075
##  STIM CHX - STIM PURO    -4.621 2.26 1628 -11.0619   1.8200  -2.047  0.3162
##  STIM CTRL - STIM PURO    3.260 2.26 1628  -3.1785   9.6985   1.445  0.6996
##
## Degrees-of-freedom method: kenward-roger
## Confidence level used: 0.95
## Conf-level adjustment: tukey method for comparing a family of 6 estimates
## P value adjustment: tukey method for comparing a family of 6 estimates
```

```r
emmip(m7, condition ~ treatment) +
  theme_bw()+
  xlab('Treatment') +
  ylab('Value')
```

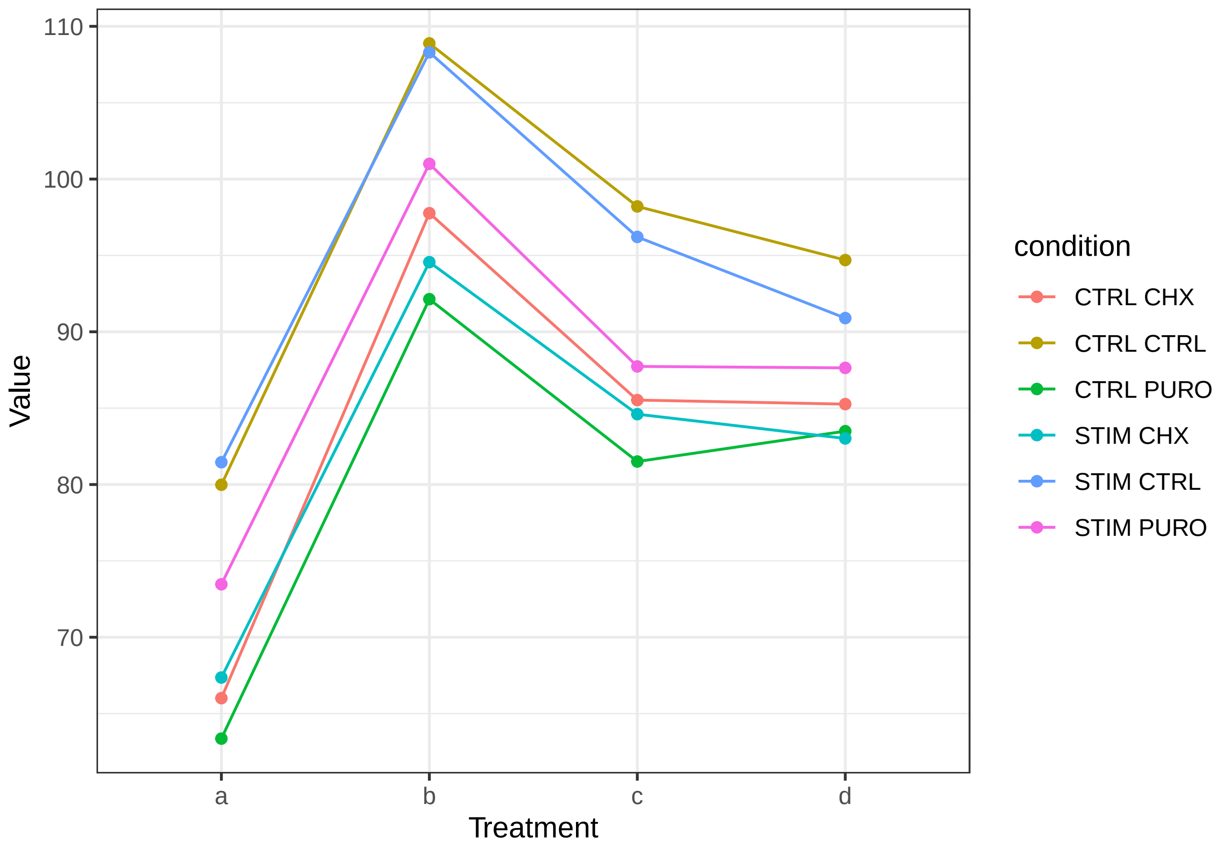


```
summary(pairs(emmeans(m7, "condition",by = "treatment",level = 0.95, infer = TRUE)))
```

```
## treatment = a:
##  contrast                estimate   SE   df t.ratio p.value
##  CTRL CHX - CTRL CTRL     -13.970 2.26 1628  -6.189  <.0001
##  CTRL CHX - CTRL PURO       2.648 2.17 1628   1.223  0.8259
##  CTRL CHX - STIM CHX       -1.350 2.26 1628  -0.598  0.9912
##  CTRL CHX - STIM CTRL     -15.446 2.26 1628  -6.845  <.0001
##  CTRL CHX - STIM PURO      -7.454 2.23 1628  -3.340  0.0111
##  CTRL CTRL - CTRL PURO     16.618 2.19 1628   7.579  <.0001
##  CTRL CTRL - STIM CHX      12.620 2.28 1628   5.530  <.0001
##  CTRL CTRL - STIM CTRL     -1.477 2.28 1628  -0.647  0.9873
##  CTRL CTRL - STIM PURO      6.516 2.26 1628   2.887  0.0455
##  CTRL PURO - STIM CHX      -3.999 2.19 1628  -1.824  0.4506
##  CTRL PURO - STIM CTRL    -18.095 2.19 1628  -8.254  <.0001
##  CTRL PURO - STIM PURO    -10.102 2.17 1628  -4.663  <.0001
##  STIM CHX - STIM CTRL     -14.096 2.28 1628  -6.177  <.0001
##  STIM CHX - STIM PURO      -6.104 2.26 1628  -2.704  0.0750
##  STIM CTRL - STIM PURO      7.993 2.26 1628   3.542  0.0055
##
## treatment = b:
##  contrast                estimate   SE   df t.ratio p.value
##  CTRL CHX - CTRL CTRL     -11.118 2.26 1628  -4.925  <.0001
##  CTRL CHX - CTRL PURO       5.632 2.17 1628   2.600  0.0978
##  CTRL CHX - STIM CHX        3.206 2.26 1628   1.421  0.7146
```

```
##  CTRL CHX - STIM CTRL   -10.530 2.26 1628  -4.666  <.0001
##  CTRL CHX - STIM PURO    -3.230 2.23 1628  -1.447  0.6979
##  CTRL CTRL - CTRL PURO   16.749 2.19 1628   7.639  <.0001
##  CTRL CTRL - STIM CHX    14.324 2.28 1628   6.277  <.0001
##  CTRL CTRL - STIM CTRL    0.587 2.28 1628   0.257  0.9998
##  CTRL CTRL - STIM PURO    7.887 2.26 1628   3.494  0.0065
##  CTRL PURO - STIM CHX    -2.426 2.19 1628  -1.106  0.8789
##  CTRL PURO - STIM CTRL  -16.162 2.19 1628  -7.373  <.0001
##  CTRL PURO - STIM PURO   -8.862 2.17 1628  -4.091  0.0006
##  STIM CHX - STIM CTRL   -13.737 2.28 1628  -6.020  <.0001
##  STIM CHX - STIM PURO    -6.436 2.26 1628  -2.851  0.0503
##  STIM CTRL - STIM PURO    7.300 2.26 1628   3.235  0.0157
##
## treatment = c:
##  contrast              estimate   SE   df t.ratio p.value
##  CTRL CHX - CTRL CTRL   -12.679 2.26 1628  -5.617  <.0001
##  CTRL CHX - CTRL PURO     4.024 2.17 1628   1.858  0.4289
##  CTRL CHX - STIM CHX      0.925 2.26 1628   0.410  0.9985
##  CTRL CHX - STIM CTRL   -10.682 2.26 1628  -4.734  <.0001
##  CTRL CHX - STIM PURO    -2.203 2.23 1628  -0.987  0.9221
##  CTRL CTRL - CTRL PURO   16.704 2.19 1628   7.618  <.0001
##  CTRL CTRL - STIM CHX    13.604 2.28 1628   5.962  <.0001
##  CTRL CTRL - STIM CTRL    1.997 2.28 1628   0.875  0.9524
##  CTRL CTRL - STIM PURO   10.476 2.26 1628   4.641  0.0001
##  CTRL PURO - STIM CHX    -3.099 2.19 1628  -1.414  0.7188
##  CTRL PURO - STIM CTRL  -14.707 2.19 1628  -6.709  <.0001
##  CTRL PURO - STIM PURO   -6.227 2.17 1628  -2.875  0.0470
##  STIM CHX - STIM CTRL   -11.607 2.28 1628  -5.087  <.0001
##  STIM CHX - STIM PURO    -3.128 2.26 1628  -1.386  0.7358
##  STIM CTRL - STIM PURO    8.479 2.26 1628   3.757  0.0024
##
## treatment = d:
##  contrast              estimate   SE   df t.ratio p.value
##  CTRL CHX - CTRL CTRL    -9.430 2.26 1628  -4.178  0.0004
##  CTRL CHX - CTRL PURO     1.773 2.17 1628   0.818  0.9642
##  CTRL CHX - STIM CHX      2.248 2.26 1628   0.996  0.9193
##  CTRL CHX - STIM CTRL    -5.633 2.26 1628  -2.496  0.1258
##  CTRL CHX - STIM PURO    -2.373 2.23 1628  -1.063  0.8959
##  CTRL CTRL - CTRL PURO   11.203 2.19 1628   5.109  <.0001
##  CTRL CTRL - STIM CHX    11.679 2.28 1628   5.118  <.0001
##  CTRL CTRL - STIM CTRL    3.798 2.28 1628   1.664  0.5558
##  CTRL CTRL - STIM PURO    7.058 2.26 1628   3.127  0.0222
##  CTRL PURO - STIM CHX     0.475 2.19 1628   0.217  0.9999
##  CTRL PURO - STIM CTRL   -7.405 2.19 1628  -3.378  0.0097
##  CTRL PURO - STIM PURO   -4.146 2.17 1628  -1.914  0.3940
##  STIM CHX - STIM CTRL    -7.881 2.28 1628  -3.454  0.0075
##  STIM CHX - STIM PURO    -4.621 2.26 1628  -2.047  0.3162
##  STIM CTRL - STIM PURO    3.260 2.26 1628   1.445  0.6996
##
## Degrees-of-freedom method: kenward-roger
## P value adjustment: tukey method for comparing a family of 6 estimates
```

## OCR - MitoStress

```
seahorse8$time <- as.numeric(seahorse8$time)
ggplot(seahorse8, aes(time, value, colour=condition))+
  geom_point()+
  theme_bw()+
  xlab("Time (min)") +
  ylab('Value')+
  facet_wrap(~ind)
```

```
## Warning: Removed 4 rows containing missing values (`geom_point()`).
```

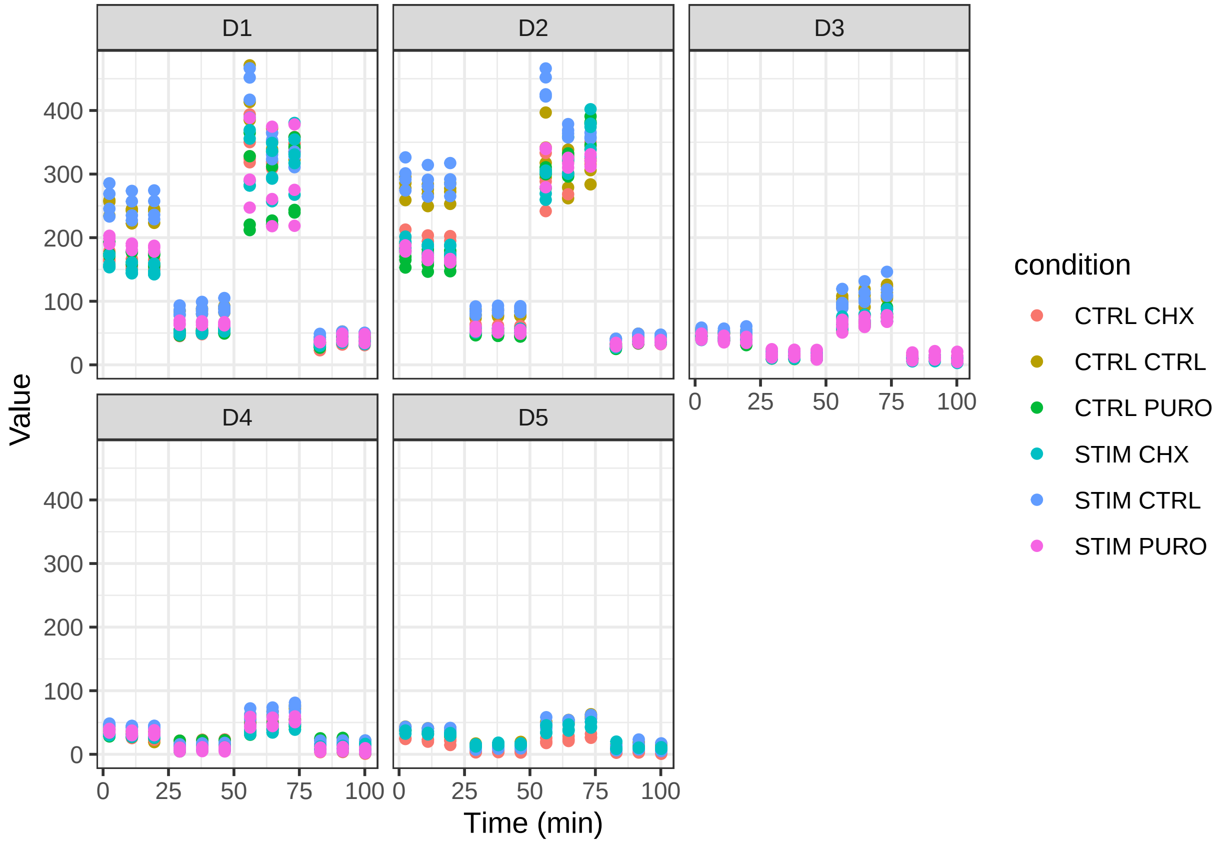


```
m8<-lmer(value ~ treatment*condition + (1|ind), data=seahorse8)
summary(m8)
```

```
## Linear mixed model fit by REML. t-tests use Satterthwaite's method [
## lmerModLmerTest]
## Formula: value ~ treatment * condition + (1 | ind)
##    Data: seahorse8
##
## REML criterion at convergence: 14524.2
##
## Scaled residuals:
```

```
##     Min      1Q  Median      3Q     Max
## -1.7425 -0.8971 -0.0585  0.7467  4.2241
##
## Random effects:
##  Groups   Name        Variance Std.Dev.
##  ind      (Intercept) 5229     72.31
##  Residual             2726     52.21
## Number of obs: 1364, groups:  ind, 5
##
## Fixed effects:
##                              Estimate Std. Error        df t value Pr(>|t|)
## (Intercept)                   88.7471    33.0720    4.3568   2.683 0.050307
## treatmentb                   -66.2506     9.7792 1335.9999  -6.775 1.86e-11
## treatmentc                    81.4053     9.7792 1335.9999   8.324  < 2e-16
## treatmentd                   -82.6012     9.9635 1336.0014  -8.290 2.72e-16
## conditionCTRL CTRL            35.5042     9.6657 1336.0104   3.673 0.000249
## conditionCTRL PURO            -0.7141    10.0898 1336.0389  -0.071 0.943591
## conditionSTIM CHX             -2.0430     9.5451 1336.0016  -0.214 0.830551
## conditionSTIM CTRL            42.0891     9.3542 1336.0124   4.500 7.40e-06
## conditionSTIM PURO             3.3675    10.6465 1336.0373   0.316 0.751826
## treatmentb:conditionCTRL CTRL  -13.6318    13.6559 1335.9999  -0.998 0.318348
## treatmentc:conditionCTRL CTRL  -31.3894    13.6559 1335.9999  -2.299 0.021682
## treatmentd:conditionCTRL CTRL  -14.2638    13.7885 1336.0007  -1.034 0.301102
## treatmentb:conditionCTRL PURO   -9.5426    14.2308 1335.9999  -0.671 0.502618
## treatmentc:conditionCTRL PURO    2.3973    14.2308 1335.9999   0.168 0.866249
## treatmentd:conditionCTRL PURO   -1.3527    14.3581 1336.0006  -0.094 0.924955
## treatmentb:conditionSTIM CHX     4.4488    13.4966 1335.9999   0.330 0.741738
## treatmentc:conditionSTIM CHX    -4.6656    13.4966 1335.9999  -0.346 0.729630
## treatmentd:conditionSTIM CHX    11.6550    13.6307 1336.0007   0.855 0.392672
## treatmentb:conditionSTIM CTRL  -17.4088    13.2149 1335.9999  -1.317 0.187943
## treatmentc:conditionSTIM CTRL  -24.6700    13.2149 1335.9999  -1.867 0.062144
## treatmentd:conditionSTIM CTRL  -17.8628    13.3519 1336.0007  -1.338 0.181173
## treatmentb:conditionSTIM PURO    0.6372    15.0140 1335.9999   0.042 0.966157
## treatmentc:conditionSTIM PURO  -14.3195    15.0140 1335.9999  -0.954 0.340386
## treatmentd:conditionSTIM PURO    5.3219    15.1347 1336.0005   0.352 0.725165
##
## (Intercept)                   .
## treatmentb                    ***
## treatmentc                    ***
## treatmentd                    ***
## conditionCTRL CTRL            ***
## conditionCTRL PURO
## conditionSTIM CHX
## conditionSTIM CTRL            ***
## conditionSTIM PURO
## treatmentb:conditionCTRL CTRL
## treatmentc:conditionCTRL CTRL *
## treatmentd:conditionCTRL CTRL
## treatmentb:conditionCTRL PURO
## treatmentc:conditionCTRL PURO
## treatmentd:conditionCTRL PURO
## treatmentb:conditionSTIM CHX
## treatmentc:conditionSTIM CHX
## treatmentd:conditionSTIM CHX
```

```
## treatmentb:conditionSTIM CTRL
## treatmentc:conditionSTIM CTRL .
## treatmentd:conditionSTIM CTRL
## treatmentb:conditionSTIM PURO
## treatmentc:conditionSTIM PURO
## treatmentd:conditionSTIM PURO
## ---
## Signif. codes:  0 '***' 0.001 '**' 0.01 '*' 0.05 '.' 0.1 ' ' 1

##
## Correlation matrix not shown by default, as p = 24 > 12.
## Use print(x, correlation=TRUE)  or
##     vcov(x)        if you need it
```

```
summary(emmeans(m8, pairwise ~ condition|treatment, infer=T))
```

```
## $emmeans
## treatment = a:
##  condition emmean   SE   df lower.CL upper.CL t.ratio p.value
##  CTRL CHX    88.75 33.1 4.36   -0.184    177.7   2.683  0.0503
##  CTRL CTRL 124.25 33.0 4.34   35.278    213.2   3.761  0.0171
##  CTRL PURO  88.03 33.2 4.41   -0.793    176.9   2.654  0.0513
##  STIM CHX   86.70 33.0 4.32   -2.306    175.7   2.627  0.0540
##  STIM CTRL 130.84 32.9 4.29   41.757    219.9   3.971  0.0144
##  STIM PURO  92.11 33.3 4.50    3.466    180.8   2.763  0.0445
##
## treatment = b:
##  condition emmean   SE   df lower.CL upper.CL t.ratio p.value
##  CTRL CHX   22.50 33.1 4.36  -66.434    111.4   0.680  0.5308
##  CTRL CTRL  44.37 33.0 4.34  -44.604    133.3   1.343  0.2452
##  CTRL PURO  12.24 33.2 4.41  -76.586    101.1   0.369  0.7292
##  STIM CHX   24.90 33.0 4.32  -64.108    113.9   0.755  0.4896
##  STIM CTRL  47.18 32.9 4.29  -41.903    136.3   1.432  0.2208
##  STIM PURO  26.50 33.3 4.50  -62.148    115.1   0.795  0.4665
##
## treatment = c:
##  condition emmean   SE   df lower.CL upper.CL t.ratio p.value
##  CTRL CHX  170.15 33.1 4.36   81.221    259.1   5.145  0.0054
##  CTRL CTRL 174.27 33.0 4.34   85.294    263.2   5.275  0.0049
##  CTRL PURO 171.84 33.2 4.41   83.010    260.7   5.181  0.0050
##  STIM CHX  163.44 33.0 4.32   74.433    252.5   4.952  0.0063
##  STIM CTRL 187.57 32.9 4.29   98.492    276.7   5.693  0.0038
##  STIM PURO 159.20 33.3 4.50   70.552    247.8   4.775  0.0066
##
## treatment = d:
##  condition emmean   SE   df lower.CL upper.CL t.ratio p.value
##  CTRL CHX    6.15 33.1 4.39  -82.722     95.0   0.186  0.8611
##  CTRL CTRL  27.39 33.0 4.34  -61.587    116.4   0.829  0.4503
##  CTRL PURO   4.08 33.2 4.41  -84.747     92.9   0.123  0.9075
##  STIM CHX   15.76 33.0 4.32  -73.252    104.8   0.477  0.6562
##  STIM CTRL  30.37 32.9 4.29  -58.707    119.5   0.922  0.4054
##  STIM PURO  14.84 33.3 4.50  -73.813    103.5   0.445  0.6769
##
```

```
## Degrees-of-freedom method: kenward-roger
## Confidence level used: 0.95
##
## $contrasts
## treatment = a:
##  contrast              estimate    SE   df lower.CL upper.CL t.ratio p.value
##  CTRL CHX - CTRL CTRL   -35.504  9.67 1336  -63.089    -7.92  -3.673  0.0034
##  CTRL CHX - CTRL PURO     0.714 10.09 1336  -28.081    29.51   0.071  1.0000
##  CTRL CHX - STIM CHX      2.043  9.55 1336  -25.197    29.28   0.214  0.9999
##  CTRL CHX - STIM CTRL   -42.089  9.35 1336  -68.785   -15.39  -4.499  0.0001
##  CTRL CHX - STIM PURO    -3.367 10.65 1336  -33.751    27.02  -0.316  0.9996
##  CTRL CTRL - CTRL PURO   36.218  9.98 1336    7.724    64.71   3.627  0.0040
##  CTRL CTRL - STIM CHX    37.547  9.42 1336   10.651    64.44   3.984  0.0010
##  CTRL CTRL - STIM CTRL   -6.585  9.22 1336  -32.889    19.72  -0.714  0.9802
##  CTRL CTRL - STIM PURO   32.137 10.54 1336    2.054    62.22   3.049  0.0283
##  CTRL PURO - STIM CHX     1.329  9.86 1336  -26.802    29.46   0.135  1.0000
##  CTRL PURO - STIM CTRL  -42.803  9.69 1336  -70.457   -15.15  -4.417  0.0002
##  CTRL PURO - STIM PURO   -4.082 10.89 1336  -35.151    26.99  -0.375  0.9990
##  STIM CHX - STIM CTRL   -44.132  9.11 1336  -70.117   -18.15  -4.847  <.0001
##  STIM CHX - STIM PURO    -5.410 10.42 1336  -35.154    24.33  -0.519  0.9954
##  STIM CTRL - STIM PURO   38.722 10.26 1336    9.428    68.02   3.772  0.0023
##
## treatment = b:
##  contrast              estimate    SE   df lower.CL upper.CL t.ratio p.value
##  CTRL CHX - CTRL CTRL   -21.872  9.67 1336  -49.457     5.71  -2.263  0.2102
##  CTRL CHX - CTRL PURO    10.257 10.09 1336  -18.538    39.05   1.017  0.9125
##  CTRL CHX - STIM CHX     -2.406  9.55 1336  -29.646    24.83  -0.252  0.9999
##  CTRL CHX - STIM CTRL   -24.680  9.35 1336  -51.376     2.02  -2.638  0.0889
##  CTRL CHX - STIM PURO    -4.005 10.65 1336  -34.388    26.38  -0.376  0.9990
##  CTRL CTRL - CTRL PURO   32.129  9.98 1336    3.634    60.62   3.218  0.0166
##  CTRL CTRL - STIM CHX    19.467  9.42 1336   -7.429    46.36   2.066  0.3061
##  CTRL CTRL - STIM CTRL   -2.808  9.22 1336  -29.112    23.50  -0.305  0.9997
##  CTRL CTRL - STIM PURO   17.868 10.54 1336  -12.215    47.95   1.695  0.5353
##  CTRL PURO - STIM CHX   -12.662  9.86 1336  -40.793    15.47  -1.285  0.7936
##  CTRL PURO - STIM CTRL  -34.937  9.69 1336  -62.591    -7.28  -3.605  0.0044
##  CTRL PURO - STIM PURO  -14.261 10.89 1336  -45.331    16.81  -1.310  0.7796
##  STIM CHX - STIM CTRL   -22.275  9.11 1336  -48.260     3.71  -2.446  0.1413
##  STIM CHX - STIM PURO    -1.599 10.42 1336  -31.342    28.14  -0.153  1.0000
##  STIM CTRL - STIM PURO   20.676 10.26 1336   -8.618    49.97   2.014  0.3346
##
## treatment = c:
##  contrast              estimate    SE   df lower.CL upper.CL t.ratio p.value
##  CTRL CHX - CTRL CTRL    -4.115  9.67 1336  -31.699    23.47  -0.426  0.9982
##  CTRL CHX - CTRL PURO    -1.683 10.09 1336  -30.478    27.11  -0.167  1.0000
##  CTRL CHX - STIM CHX      6.709  9.55 1336  -20.532    33.95   0.703  0.9816
##  CTRL CHX - STIM CTRL   -17.419  9.35 1336  -44.115     9.28  -1.862  0.4262
##  CTRL CHX - STIM PURO    10.952 10.65 1336  -19.432    41.34   1.029  0.9084
##  CTRL CTRL - CTRL PURO    2.432  9.98 1336  -26.063    30.93   0.244  0.9999
##  CTRL CTRL - STIM CHX    10.823  9.42 1336  -16.072    37.72   1.148  0.8609
##  CTRL CTRL - STIM CTRL  -13.304  9.22 1336  -39.609    13.00  -1.443  0.7003
##  CTRL CTRL - STIM PURO   15.067 10.54 1336  -15.016    45.15   1.429  0.7091
##  CTRL PURO - STIM CHX     8.392  9.86 1336  -19.739    36.52   0.851  0.9576
##  CTRL PURO - STIM CTRL  -15.736  9.69 1336  -43.390    11.92  -1.624  0.5828
##  CTRL PURO - STIM PURO   12.635 10.89 1336  -18.435    43.71   1.161  0.8554
```

```
##  STIM CHX - STIM CTRL   -24.128  9.11 1336  -50.113     1.86  -2.650  0.0864
##  STIM CHX - STIM PURO     4.243 10.42 1336  -25.500    33.99   0.407  0.9986
##  STIM CTRL - STIM PURO   28.371 10.26 1336   -0.922    57.66   2.764  0.0640
##
## treatment = d:
##  contrast              estimate    SE   df lower.CL upper.CL t.ratio p.value
##  CTRL CHX - CTRL CTRL   -21.240  9.86 1336  -49.369     6.89  -2.155  0.2599
##  CTRL CHX - CTRL PURO     2.067 10.27 1336  -27.240    31.37   0.201  1.0000
##  CTRL CHX - STIM CHX     -9.612  9.74 1336  -37.396    18.17  -0.987  0.9221
##  CTRL CHX - STIM CTRL   -24.226  9.55 1336  -51.488     3.04  -2.536  0.1145
##  CTRL CHX - STIM PURO    -8.689 10.82 1336  -39.556    22.18  -0.803  0.9669
##  CTRL CTRL - CTRL PURO   23.307  9.98 1336   -5.188    51.80   2.334  0.1810
##  CTRL CTRL - STIM CHX    11.628  9.42 1336  -15.267    38.52   1.234  0.8202
##  CTRL CTRL - STIM CTRL   -2.986  9.22 1336  -29.290    23.32  -0.324  0.9995
##  CTRL CTRL - STIM PURO   12.551 10.54 1336  -17.531    42.63   1.191  0.8415
##  CTRL PURO - STIM CHX   -11.679  9.86 1336  -39.810    16.45  -1.185  0.8442
##  CTRL PURO - STIM CTRL  -26.293  9.69 1336  -53.947     1.36  -2.713  0.0732
##  CTRL PURO - STIM PURO  -10.756 10.89 1336  -41.826    20.31  -0.988  0.9219
##  STIM CHX - STIM CTRL   -14.614  9.11 1336  -40.600    11.37  -1.605  0.5953
##  STIM CHX - STIM PURO     0.923 10.42 1336  -28.821    30.67   0.089  1.0000
##  STIM CTRL - STIM PURO   15.537 10.26 1336  -13.757    44.83   1.514  0.6555
##
## Degrees-of-freedom method: kenward-roger
## Confidence level used: 0.95
## Conf-level adjustment: tukey method for comparing a family of 6 estimates
## P value adjustment: tukey method for comparing a family of 6 estimates
```

```
emmip(m8, condition ~ treatment) +
  theme_bw()+
  xlab('Treatment') +
  ylab('Value')
```

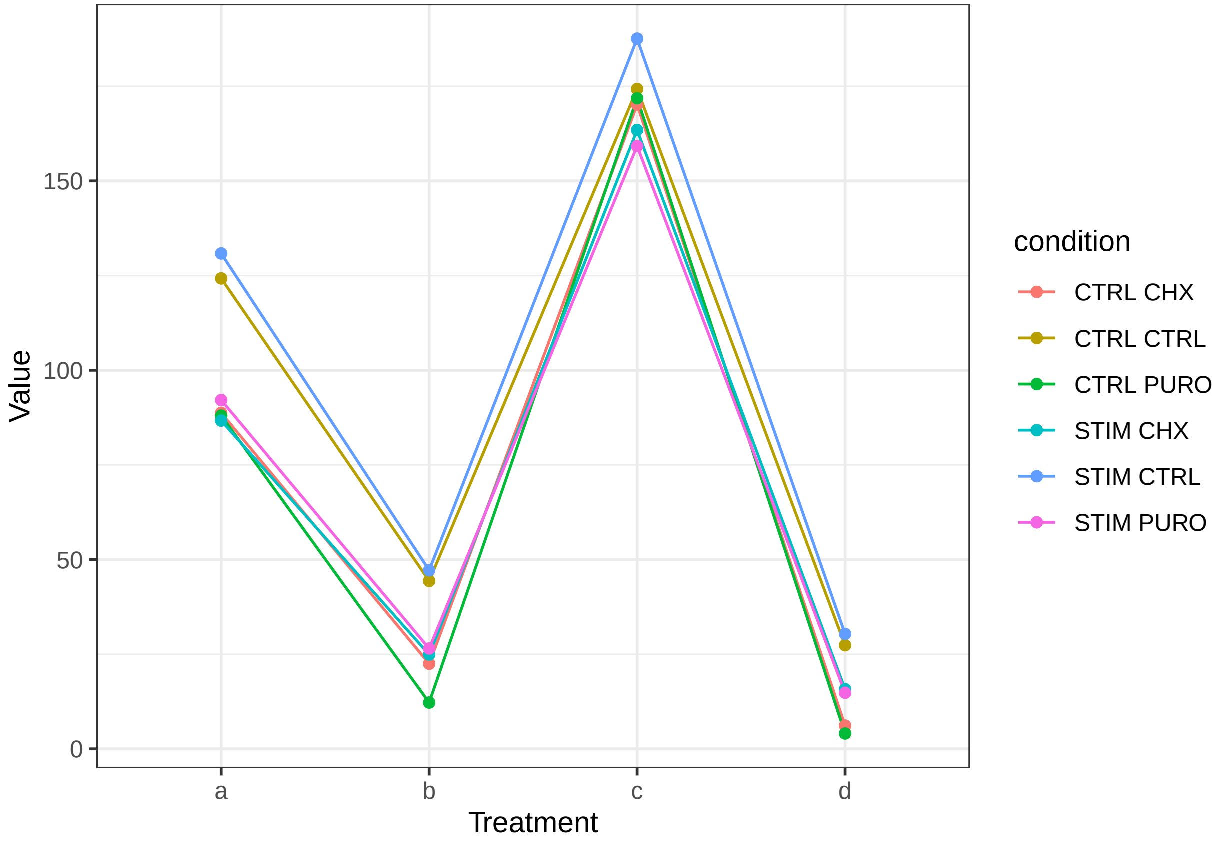


```
summary(pairs(emmeans(m8, "condition",by = "treatment",level = 0.95, infer = TRUE)))
```

```
## treatment = a:
##  contrast               estimate    SE   df t.ratio p.value
##  CTRL CHX - CTRL CTRL    -35.504  9.67 1336  -3.673  0.0034
##  CTRL CHX - CTRL PURO      0.714 10.09 1336   0.071  1.0000
##  CTRL CHX - STIM CHX       2.043  9.55 1336   0.214  0.9999
##  CTRL CHX - STIM CTRL    -42.089  9.35 1336  -4.499  0.0001
##  CTRL CHX - STIM PURO     -3.367 10.65 1336  -0.316  0.9996
##  CTRL CTRL - CTRL PURO    36.218  9.98 1336   3.627  0.0040
##  CTRL CTRL - STIM CHX     37.547  9.42 1336   3.984  0.0010
##  CTRL CTRL - STIM CTRL    -6.585  9.22 1336  -0.714  0.9802
##  CTRL CTRL - STIM PURO    32.137 10.54 1336   3.049  0.0283
##  CTRL PURO - STIM CHX      1.329  9.86 1336   0.135  1.0000
##  CTRL PURO - STIM CTRL   -42.803  9.69 1336  -4.417  0.0002
##  CTRL PURO - STIM PURO    -4.082 10.89 1336  -0.375  0.9990
##  STIM CHX - STIM CTRL    -44.132  9.11 1336  -4.847  <.0001
##  STIM CHX - STIM PURO     -5.410 10.42 1336  -0.519  0.9954
##  STIM CTRL - STIM PURO    38.722 10.26 1336   3.772  0.0023
##
## treatment = b:
##  contrast               estimate    SE   df t.ratio p.value
##  CTRL CHX - CTRL CTRL    -21.872  9.67 1336  -2.263  0.2102
##  CTRL CHX - CTRL PURO     10.257 10.09 1336   1.017  0.9125
##  CTRL CHX - STIM CHX      -2.406  9.55 1336  -0.252  0.9999
```

```
##  CTRL CHX - STIM CTRL   -24.680  9.35 1336  -2.638  0.0889
##  CTRL CHX - STIM PURO    -4.005 10.65 1336  -0.376  0.9990
##  CTRL CTRL - CTRL PURO   32.129  9.98 1336   3.218  0.0166
##  CTRL CTRL - STIM CHX    19.467  9.42 1336   2.066  0.3061
##  CTRL CTRL - STIM CTRL   -2.808  9.22 1336  -0.305  0.9997
##  CTRL CTRL - STIM PURO   17.868 10.54 1336   1.695  0.5353
##  CTRL PURO - STIM CHX   -12.662  9.86 1336  -1.285  0.7936
##  CTRL PURO - STIM CTRL  -34.937  9.69 1336  -3.605  0.0044
##  CTRL PURO - STIM PURO  -14.261 10.89 1336  -1.310  0.7796
##  STIM CHX - STIM CTRL   -22.275  9.11 1336  -2.446  0.1413
##  STIM CHX - STIM PURO    -1.599 10.42 1336  -0.153  1.0000
##  STIM CTRL - STIM PURO   20.676 10.26 1336   2.014  0.3346
##
## treatment = c:
##  contrast              estimate    SE   df t.ratio p.value
##  CTRL CHX - CTRL CTRL    -4.115  9.67 1336  -0.426  0.9982
##  CTRL CHX - CTRL PURO    -1.683 10.09 1336  -0.167  1.0000
##  CTRL CHX - STIM CHX      6.709  9.55 1336   0.703  0.9816
##  CTRL CHX - STIM CTRL   -17.419  9.35 1336  -1.862  0.4262
##  CTRL CHX - STIM PURO    10.952 10.65 1336   1.029  0.9084
##  CTRL CTRL - CTRL PURO    2.432  9.98 1336   0.244  0.9999
##  CTRL CTRL - STIM CHX    10.823  9.42 1336   1.148  0.8609
##  CTRL CTRL - STIM CTRL  -13.304  9.22 1336  -1.443  0.7003
##  CTRL CTRL - STIM PURO   15.067 10.54 1336   1.429  0.7091
##  CTRL PURO - STIM CHX     8.392  9.86 1336   0.851  0.9576
##  CTRL PURO - STIM CTRL  -15.736  9.69 1336  -1.624  0.5828
##  CTRL PURO - STIM PURO   12.635 10.89 1336   1.161  0.8554
##  STIM CHX - STIM CTRL   -24.128  9.11 1336  -2.650  0.0864
##  STIM CHX - STIM PURO     4.243 10.42 1336   0.407  0.9986
##  STIM CTRL - STIM PURO   28.371 10.26 1336   2.764  0.0640
##
## treatment = d:
##  contrast              estimate    SE   df t.ratio p.value
##  CTRL CHX - CTRL CTRL   -21.240  9.86 1336  -2.155  0.2599
##  CTRL CHX - CTRL PURO     2.067 10.27 1336   0.201  1.0000
##  CTRL CHX - STIM CHX     -9.612  9.74 1336  -0.987  0.9221
##  CTRL CHX - STIM CTRL   -24.226  9.55 1336  -2.536  0.1145
##  CTRL CHX - STIM PURO    -8.689 10.82 1336  -0.803  0.9669
##  CTRL CTRL - CTRL PURO   23.307  9.98 1336   2.334  0.1810
##  CTRL CTRL - STIM CHX    11.628  9.42 1336   1.234  0.8202
##  CTRL CTRL - STIM CTRL   -2.986  9.22 1336  -0.324  0.9995
##  CTRL CTRL - STIM PURO   12.551 10.54 1336   1.191  0.8415
##  CTRL PURO - STIM CHX   -11.679  9.86 1336  -1.185  0.8442
##  CTRL PURO - STIM CTRL  -26.293  9.69 1336  -2.713  0.0732
##  CTRL PURO - STIM PURO  -10.756 10.89 1336  -0.988  0.9219
##  STIM CHX - STIM CTRL   -14.614  9.11 1336  -1.605  0.5953
##  STIM CHX - STIM PURO     0.923 10.42 1336   0.089  1.0000
##  STIM CTRL - STIM PURO   15.537 10.26 1336   1.514  0.6555
##
## Degrees-of-freedom method: kenward-roger
## P value adjustment: tukey method for comparing a family of 6 estimates
```
